# Unraveling the Photoisomerization Mechanism of Group‑8 Fulvalene-Bridged Bimetallic Complexes for Molecular Solar–Thermal Energy Storage

**DOI:** 10.1021/jacs.5c14186

**Published:** 2025-10-30

**Authors:** Gaurab Ganguly, Leticia González

**Affiliations:** Institute of Theoretical Chemistry, Faculty of Chemistry, 27258 University of Vienna, Währinger Str. 17, 1090 Vienna, Austria

## Abstract

The (fulvalene)­Ru_2_(CO)_4_ complex is the only known metal-based photoswitch capable of both harvesting solar energy upon irradiation and releasing it on demand, acting as a molecular solar–thermal fuel. Its photochemistry is unique and strongly wavelength-dependent: irradiation above 350 nm generates a “long-lived” (∼2 ns) triplet biradical intermediate that drives photoisomerization, whereas excitation below 300 nm induces only minor decarbonylation. Interestingly, replacing Ru with other Group-8 metals either hinders efficient solar-energy harvesting or thermal energy release: in the (fulvalene)­Fe_2_(CO)_4_ analogue, photoisomerization is suppressed, leading exclusively to photodecarbonylation; the heterobimetallic RuFe complex is photoinert, and the heavier Os_2_ congener photoisomerizes but cannot thermally release the stored energy. Here, we rationalize the different photoisomerization mechanisms using *ab initio* multiconfigurational methods and Marcus theory. We find that photoconversion is determined by the accessibility of the singlet–triplet crossing. While intersystem crossing (ISC) is barrierless in the Marcus crossover regime and thus ultrafast in the Ru_2_ and Os_2_ complexes, forming the triplet biradical, the Fe_2_ and RuFe analogues are trapped behind a high ISC barrier in the Marcus-inverted regime and therefore do not photoisomerize. When formed, triplet biradicals are stabilized due to negligible spin–orbit coupling with the singlet ground state, suppressing metal–metal bond reformation and favoring photoisomerization. A complementary analysis of the metal–carbonyl bonding rationalizes the wavelength-dependent decarbonylation in both the Ru_2_ and Fe_2_ complexes. This work provides a mechanistic understanding of the different excited-state behaviors of fulvalene-based bimetallic tetracarbonyls and a predictive framework for designing photoactive metal complexes for solar energy storage.

## Graphical abstract

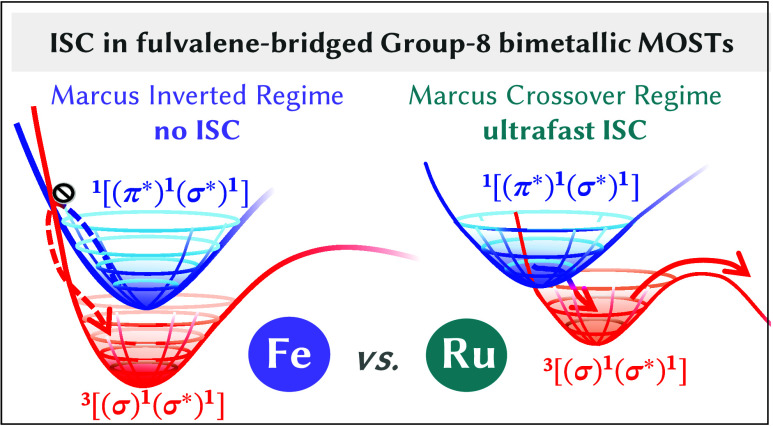

## Introduction

Intersystem crossing (ISC) plays a central role in the excited-state dynamics of transition-metal complexes, fundamentally shaping their photophysical and photochemical behavior.^1^ The contrasting relaxation dynamics following ISC in the widely studied photosensitizers [M­(bpy)_3_]^2+^ (M = Ru, Fe; bpy = bipyridine) clearly illustrate how differences in the metal orbital character between first- (3*d*) and second-row (4*d*) transition metals influence their photophysics: while the Ru-based complex exhibits long-lived excited states, the Fe analogue deactivates to the ground state much more rapidly.^2–5^ Similarly, the photochemistry of the bimetallic complexes (Fv)­M_2_(CO)_4_ (Fv = fulvalene, M = Ru, Fe) also differs dramatically with the metal center, yet the origin of the contrasting behavior remains poorly understood.

The (Fv)­Ru_2_(CO)_4_ complex functions as a photoswitch and represents the first—and to date, only—transition-metal-based molecular solar–thermal (MOST) energy storage system, distinguishing it from the few MOST systems reported in the literature.^6–11^ Upon excitation, it stores ∼0.85 eV of energy by converting into a higher-energy isomer with a long shelf life; when heated or treated with a catalyst (e.g. AgNO_3_−SiO_2_), it reverts to the original form, releasing heat and thus functioning as a rechargeable molecular heat battery (Scheme 1A).^6–11^ Time-resolved infrared and X-ray spectroscopies have shown that irradiation of (Fv)­Ru_2_(CO)_4_ with light above 350 nm induces cleavage of the Ru–Ru bond and triggers photoisomerization.^7^ Both the parent complex and its photoisomer lie on singlet potential energy surfaces (PES); however, the photoisomerization proceeds via sequential ISCs through a “long-lived” (∼2 ns) triplet biradical intermediate, involving reversible transitions between the singlet and the triplet manifolds (Scheme 1B).^7^ In contrast, excitation below 300 nm leads to a competing photodecarbonylation (CO loss) pathway.^7^

**Scheme 1:**
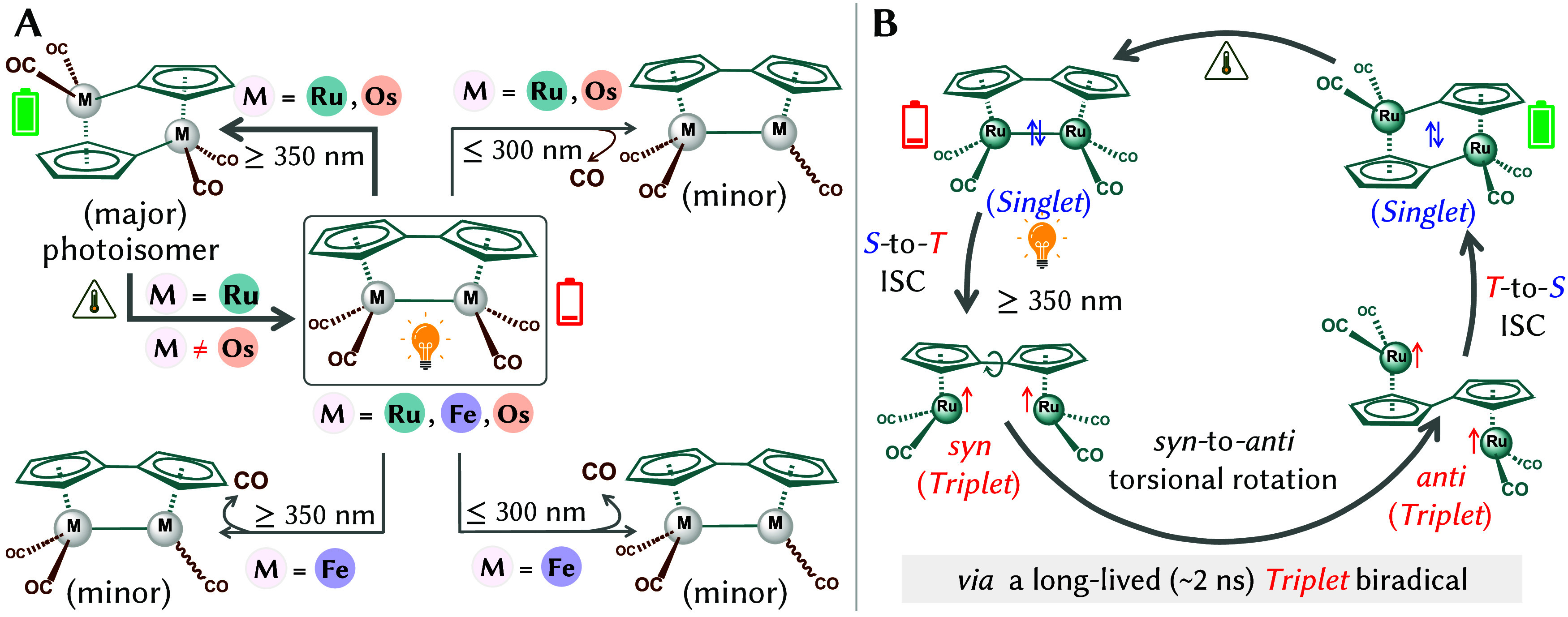
Photochemistry of Fulvalene-Bridged Group-8 Bimetallic Tetracarbonyls ((Fv)­M_2_(CO)_4_, M = Fe, Ru, Os)^a^

a (A) Schematic overview of wavelength-dependent reaction pathways reported experimentally for (Fv)­Ru_2_(CO)_4_,^7,10,14,17^ (Fv)­Fe_2_(CO)_4_,^12,17^ and (Fv)­Os_2_(CO)_4_.^10,14^ (B) Proposed photoisomerization cycle via the triplet biradical intermediate and thermal back-reaction in (Fv)­Ru_2_(CO)_4_.^6,7^ Scheme based on experimental data reported in refs 6,7,10,14,17 .

Intriguingly, similar experiments on (Fv)­Fe_2_(CO)_4_ ^12^—in which Ru is replaced with the cheaper and Earth-abundant Fe—did not detect the photoisomerization product or any triplet intermediate; instead, only the minor decarbonylation pathway was observed regardless of wavelength (Scheme 1A). Also the heterobimetallic (Fv)­RuFe­(CO)_4_,^13^ which combines Ru’s efficient ISC with Fe’s sustainability was found photoinert,^10^ whereas the heavier (Fv)­Os_2_(CO)_4_ analogue photoisomerizes efficiently but cannot thermally undergo the back-reaction, rendering it unsuitable for MOST applications.^10,14^ In passing, we note that fulvalene-bridged Mo_2_ and W_2_ complexes also do not undergo photoisomerization,^15^ further underscoring the unique—and yet-unexplained—nature of the (Fv)­Ru_2_(CO)_4_ framework. Spin–orbit coupling (SOC) mediating ISC^16^ increases systematically across the Group-8, 3*d*-4*d*-5*d* series (Fe, Ru, Os); however, it is not that in the Fe_2_ complex the triplet biradical intermediate forms more slowly—but it is entirely absent.^12^ While the weaker SOC of Fe_2_ could partially account for its photoinactivity, the complete absence of a triplet intermediate suggests that other factors must play a role. Furthermore, the long lifetime of the triplet biradical intermediate in the Ru_2_ complex^7^ is unusual. It has been speculated^17^ that this extended triplet lifetime results from reduced SOC during the photoisomerization, due to spin-density localization on the fulvalene ring that could inhibit Ru–Ru bond reformation via ground-state relaxation. However, this hypothesis remains unsubstantiated.

While research on organic MOST systems continues to advance,^18–31^ progress with transition-metal counterparts—especially fulvalene-bridged bimetallic tetracarbonyls—has stalled, due to limited understanding of how spin-state dynamics modulates excited-state reactivity.^17^ Here, we present a general mechanistic framework for these fulvalene-bridged Group-8 bimetallic complexes, based on high-level multiconfigurational *ab initio* quantum chemical calculations. By mapping the excited-state PES, quantifying SOC matrix elements, and modeling ISC dynamics within the Marcus theory framework, we elucidate why the Ru_2_ complex uniquely facilitates triplet biradical formation, whereas the Fe_2_ analogue does not. Our results show that ISC efficiency is not governed by the magnitude of SOC but is primarily controlled by the activation barrier determined by the position of the excited singlet-triplet crossing along the reaction coordinate. These mechanistic insights also extend to the Os_2_ and mixed-metal RuFe complexes, illustrating their broader applicability. Moreover, we elucidate the origin of the Ru_2_ complex’s exceptional triplet biradical stability, which promotes photoisomerization and inhibits Ru–Ru bond reformation. In addition, we provide a comprehensive mechanistic account of the competing decarbonylation pathways observed experimentally.

Thus, this work resolves a long-standing mechanistic challenge in organometallic photochemistry by uncovering fundamental differences in the excited-state landscapes and ISC kinetics of fulvalene-bridged Group-8 bimetallic complexes—across the first- (3*d*), second- (4*d*), and third-row (5*d*) transition series—as exemplified by the homo- (Fe_2_, Ru_2_, and Os_2_) and hetero- (RuFe) bimetallic systems.. More broadly, these insights establish a foundation for the rational design of efficient bimetallic molecular systems to address critical challenges in solar energy storage.

## Computational Details

### Density Functional Theory Calculations

The ground-state structures of (Fv)­M_2_(CO)_4_ (M_2_ = Fe_2_, Ru_2_, Os_2_, and mixed RuFe) were optimized in the gas phase using the B3LYP hybrid functional^32,33^ with the ZORA scalar relativistic Hamiltonian^34^ and ZORA-def2-TZVPP basis set.^35^ Dispersion effects were accounted for using Grimme’s D3 (D3BJ damping) method.^36^ Vibrational frequencies were obtained at the same level of theory. The Cartesian coordinates for the optimized critical intermediates,discussed throughout this paper, are provided in Supporting Section S1. All relevant Kohn–Sham molecular orbital (MO) isosurfaces and the qualitative MO correlation diagram are provided in Supporting Section S2. Excited-state energies and oscillator strengths were computed using time-dependent density functional theory (TDDFT) with the CAM-B3LYP functional^37^ and the Tamm–Dancoff approximation (TDA)^38^ that enhances computational efficiency and mitigates triplet instabilities. Because TDA can occasionally misorder excited states in organometallic complexes,^39^ we benchmarked our (TDA)-TDDFT calculations against accurate multiconfigurational, wavefunction-based calculations (*vide infra*), which confirm that TDDFT reliably predicts both state ordering and character of the excited states. For brevity, the (TDA)-TD-CAM-B3LYP/ZORA-def2-TZVP calculations are hereafter denoted as TDDFT. All the TDDFT calculations were performed using the ORCA software (v5.0.4).^40,41^ To characterize the nature of the electronic excited states, we employ the transition density analysis implemented in the TheoDORE program package.^42^ Besides providing natural transition orbitals (NTOs), this wave function analysis tool uses charge transfer numbers to quantitatively classify electronic excited states into metal-centered and charge-transfer components, see Supporting Section S3 for details.

### Wave Function Theory Calculations

Multiconfigurational wavefunction-based calculations using the complete active space self-consistent field (CASSCF) method with ANO-R type basis sets (R3 for metals (Ru/Fe/Os), R2 for C, O, and R1 for H) were employed for both systems, with scalar relativistic effects incorporated via the X2C Hamiltonian.^43^ All orbitals shown in Supporting Section S2, except for the lower-lying δ′ and δ′* orbitals, and the higher-lying δ and δ* orbitals (the latter pair being involved in decarbonylation, *vide infra*), were included in the active space for constructing the adiabatic potential energy curves. Due to strong interactions between metal *d* orbitals and CO_π*_ orbitals, four additional unoccupied CO_π*_ orbitals were incorporated. The natural orbital analysis indicated significant contributions from double *d*-shell orbitals^44^ (*d*_π_⊥__^′^, *d*_π_∥__^′^) mixed with CO_π*_ orbitals. Consequently, two additional unoccupied *d*_σ_^′^ orbitals were added to uniformly describe σ and π interactions between metals, resulting in a 10*e*/12o active space employed to construct the adiabatic potential energy profiles. In the study of the decarbonylation mechanism, two additional δ and δ* orbitals were included, resulting in a 10*e*/14o active space (Supporting Section S4). Dynamic electron correlation was included using extended multistate CAS second-order perturbation theory (XMS-CASPT2), providing accurate ground- and excited-state energies.^45^ Empirical shifts—an IPEA shift of 0.00 for the Ru_2_ complex and 0.15 for the Fe_2_ complex—were applied to improve the description of excitation energies (Section S5), while the default value of 0.25 was used for the Os_2_ and mixed RuFe complexes. In addition, an imaginary shift of 0.2 au was employed to avoid intruder states.^46^

To construct the adiabatic potential energy curves of the three lowest singlet and triplet states, XMS-CASPT2­(10*e*,12o) was used. Natural orbital analysis for various intermediates using XMS-CASPT2­(10*e*,12o) is shown in Supporting Section S6. SOC were calculated perturbatively using the state-interaction method with the same wave functions. The matrix elements of the complex spin–orbit Hamiltonian were computed across spin components of the spin-free eigenstates, reported in cm^–1^ and presented in Supporting Section S7. All wavefunction-based calculations were performed using the OpenMOLCAS software (v22.10).^47^

## Results and Discussion

Since the most contrasting behavior is shown by the Ru_2_ and Fe_2_ complexes, we begin by investigating the vertical excitation energies of (Fv)­M_2_(CO)_4_ (M = Ru, Fe) complexes to identify key excited states driving photoisomerization and decarbonylation, focusing on orbital contributions to metal–metal bonding. We then analyze and compare the ISC mechanisms responsible for triplet state formation in both complexes, mapping excited-state surfaces and identifying the relative contributions of SOC and ISC activation energy barriers. The observed trends are further extended to the (Fv)­Os_2_(CO)_4_ and (Fv)­RuFe­(CO)_4_ complexes, which demonstrates the broader applicability of the mechanistic framework. Next, we examine the relaxation pathways of the triplet state in the Ru_2_ complex, highlighting its prolonged lifetime, its role in facilitating photoisomerization, and its deactivation pathway back to the ground state. Finally, we elucidate the wavelength-dependent decarbonylation pathway in the Ru_2_ vs Fe_2_ complexes, completing a mechanistic framework that accounts for the contrasting photochemical behavior of these systems.

### Vertical Excitations Energies in (Fv)­M_2_(CO)_4_ (M = Ru, Fe)

For (Fv)­Ru_2_(CO)_4_, the absorption onset (λ_onset_) and maximum absorption (λ_max_) were recorded at 450 and 330 nm, respectively.^11^ In contrast, (Fv)­Fe_2_(CO)_4_ exhibits a λ_onset_ at 600 nm, a shoulder at 450 nm, and additional peaks at 370 and 310 nm.^12^ This red-shift observed in the Fe_2_ complex is consistent with the weaker ligand-field splitting arising from the more contracted and less diffuse nature of 3*d* orbitals compared to 4*d*, following the periodic trend 3*d* ≪ 4*d* < 5*d*. This leads to less effective metal–ligand orbital overlap and consequently lower-energy electronic transitions. Notably, the resulting absorption band (350–600 nm) covers the most intense region of the solar UV–vis spectrum. Both (Fv)­Fe_2_(CO)_4_ and (Fv)­Ru_2_(CO)_4_ absorb within this window, making them molecular photoswitches compatible with solar irradiation. Although the Fe_2_ complex does not undergo photoisomerization upon illumination above 350 nm (Scheme 1), its absorption profile is not the limiting factor for solar thermal storage. In fact, compared to (Fv)­Ru_2_(CO)_4_, the Fe_2_ analog shows even more favorable overlap with solar light (Figure 1). Many reported MOST systems operate within this spectral range (350–600 nm), as thoroughly reviewed by Moth-Poulsen and co-workers.^48,49^ The lack of photoisomerization above 350 nm is therefore likely due to differences in excited-state dynamics rather than insufficient absorption.

**Figure 1:**
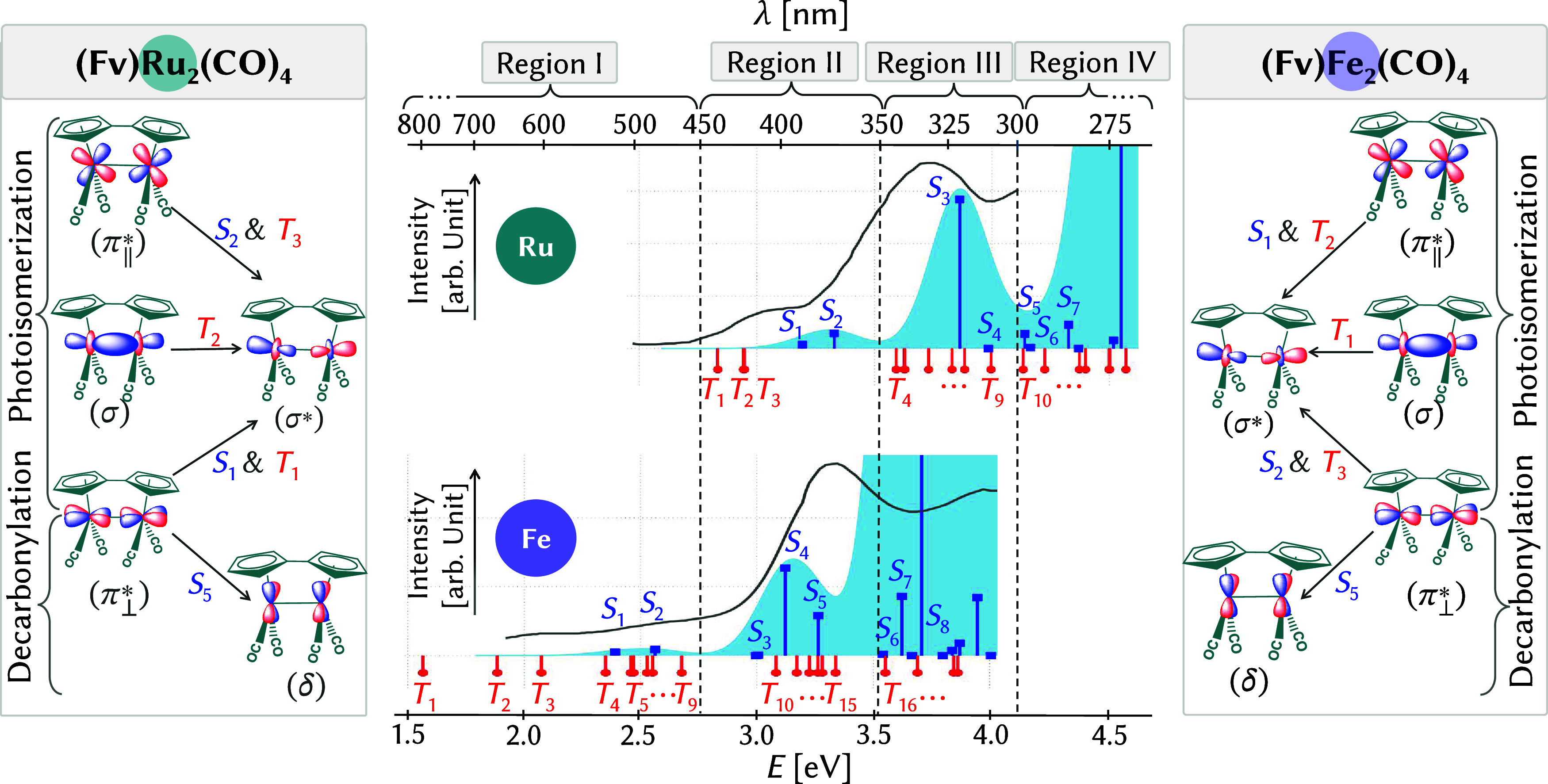
Calculated (TDDFT) vs measured UV–vis absorption spectra for (Fv)­M_2_(CO)_4_ (M = Ru, Fe). Blue and red vertical sticks represent the calculated singlet and triplet states, respectively. The sky-blue curves show Gaussian-broadened spectra, with a full-width at half-maximum of 0.15 eV, while the gray lines correspond to experimental spectra digitized from ref 11 (Ru) and ref 12 (Fe). Dashed vertical lines demarcate the spectral regions I–IV. Transitions between MOs for singlet and triplet excited states responsible for photoisomerization and decarbonylation are shown in the left (Ru) and right (Fe) panels.

To identify the excited states involved in photoisomerization and decarbonylation, we performed TDDFT calculations at the ground-state optimized structures of the parent complexes (see Supporting Section S4 for the XMS-CASPT2 spectra and analysis). The calculations indicate predominantly metal-centered states with minimal charge-transfer or double-excitation character, which accurately reproduce relative experimental peak intensities,^11,12^ particularly for the Fe_2_ complex.^12^ For ease of discussion, we divide the spectral range into four regions: > 450 nm (<2.75 eV, region I), 450–350 nm (2.75–3.50 eV, region II), 350–300 nm (3.50–4.10 eV, region III), and <300 nm (>4.10 eV, region IV), as indicated by the vertical gray dashed lines in Figure 1. Accordingly, the Ru_2_ complex absorbs predominantly in regions II, III, and IV, whereas regions I and II are most relevant for the Fe_2_ complex.

The (Fv)­Ru_2_(CO)_4_ complex exhibits initial absorption in region II, involving low-lying singlet (*S*_1_, *S*_2_) and triplet states (*T*_1_–*T*_3_), which may contribute to photoisomerization (Figure 1, middle panel). The most intense singlet transition below 300 nm (*S*_3_, region III) corresponds to the experimental λ_max_ at 330 nm,^6,7,11^ yet it does not lead to observable photochemical reactivity despite its high oscillator strength. Region III also includes a manifold of triplet states (*T*_4_–*T*_9_). At higher excitation energies, singlet states from region IV (*S*_5_ and above) are implicated in decarbonylation, indicating a distinct mechanistic pathway. In (Fv)­Fe_2_(CO)_4_, the experimental absorption below 450 nm^12^ correspond to the weak *S*_1_ and *S*_2_ singlet states and the *T*_1_–*T*_9_ triplet states (region I). Region II features brighter singlet states (*S*_3_–*S*_5_), matching the experimental peak at 370 nm,^12^ along with triplet states (*T*_10_–*T*_15_). Region III encompasses higher singlet and triplet states, starting from *S*_6_ and *T*_16_ onward.

To rationalize the nature of these excitations, we examine the underlying MOs (Supporting Section S2). In bimetallic systems with ideal *D*_∞*h*_ symmetry, metal *d* orbitals can form σ, π, and δ bonding and antibonding MOs.^50^ These include one pair of σ bonding and antibonding MOs, formed by head-on overlap along the metal–metal axis; two pairs of π bonding and antibonding MOs arising from in-plane (π_∥_) and out-of-plane (π_⊥_) side-on interactions; and two pairs of δ bonding and antibonding MOs (labeled as δ and δ′) resulting from four-lobed metal *d* orbital overlaps (Supporting Figure S3). In the ground state, the π_∥_ and π_⊥_ bonding and antibonding orbitals, as well as the δ′ pair, are doubly occupied, while the δ bonding and antibonding orbitals remain unoccupied. The single metal–metal bond solely arises from the doubly occupied bonding σ MO and its unoccupied antibonding σ* counterpart (Supporting Figure S3).

The excited states of (Fv)­M_2_(CO)_4_ (M = Ru, Fe) exhibit over 50% of metal-centered character across the photoactive regions (Supporting Section S3). Additional contributions from metal-to-ligand and ligand-to-metal charge transfer are secondary. Notably, despite the extended π-conjugation of the fulvalene ligand, these excited states do not involve significant excitation localized on the fulvalene π-system. This observation is consistent with the underlying MO framework and NTO analyses, providing a comprehensive picture of the absorption features in these complexes. The slightly higher metal-center character observed in the Fe_2_ complex relative to Ru_2_ may arise from the more contracted nature of Fe 3*d* orbitals compared to Ru 4*d* orbitals, which could lead to modest differences in orbital overlap and state composition.

In short, the triplet states (*T*_1_ to *T*_3_) in region II of (Fv)­Ru_2_(CO)_4_ correspond to π_⊥_^*^ → σ*, σ → σ*, and π_∥_^*^ → σ* transitions, while the bright singlet states (*S*_1_ and *S*_2_)—those with significant oscillator strength contributing to light absorption—are primarily associated with π_⊥_^*^ → σ* and π_∥_^*^ → σ* transitions. More bright singlet states in region IV (*S*_5_, *S*_6_, *S*_7_) are mainly linked to π_∥_^*^/π_⊥_^*^ → δ/δ* transitions (Figure 1, left panel, and Supporting Figure S4).

In (Fv)­Fe_2_(CO)_4_, the lowest triplet (*T*_1_ to *T*_3_) and bright singlet (*S*_1_ and *S*_2_) states in region I are characterized by σ → σ*, π_⊥_^*^ → σ*, and π_∥_^*^ → σ* transitions, similar to region II of (Fv)­Ru_2_(CO)_4_. Although not experimentally observed, these states could potentially contribute to photoisomerization. The *T*_4_ and *T*_5_ states (region I), along with *S*_5_ (region II), correspond to π_∥_^*^/π_⊥_^*^ → δ/δ* transitions (Figure 1, right panel, and Supporting Figure S5). These transitions, similar to region IV of (Fv)­Ru_2_(CO)_4_, likely drive decarbonylation in (Fv)­Fe_2_(CO)_4_.^7,12^

We note that TDDFT calculations with an implicit solvent model (mimicking the experimentally used low-dielectric solvent, heptane)^11^ evidence minimal solvents effects on the energies, ordering and character of the key excited states, owing to their strong metal-centered character (Supporting Table S11), justifying the use of gas phase calculations.

Although electronically inactive in the excitation process, the fulvalene bridge is structurally essential for enabling the observed photochemical behavior. In contrast to η^5^-(Cp) metal tetracarbonyl dimers, where the two metal centers are effectively decoupled due to lack of a shared framework, the η^5^: η^5^-(Fv) ligand provides a rigid yet rotationally flexible backbone that maintains intramolecular proximity. This backbone enforces a C–C torsional degree of freedom, allowing for productive metal–metal realignment during photoisomerization. Without such structural support, as in the case of simple Cp–M systems, isomerization would be disfavored due to premature separation of the metal fragments.

### Intersystem Crossing Mechanisms Responsible for Triplet Intermediate Formation

As explained above, the singlet and triplet excited states responsible for the photoisomerization of both (Fv)­Ru_2_(CO)_4_ and (Fv)­Fe_2_(CO)_4_ are characterized by σ/π_∥_^*^/π_⊥_^*^ → σ* excitations. These transitions are typical of metal–metal bonded dimers, where promotion from bonding or weakly antibonding orbitals to strongly antibonding σ* orbital may weaken the metal–metal bond and perturbs molecular geometry. These changes play a critical role in enabling ISC to a reactive triplet biradical intermediate that drives the photoisomerization process. To evaluate the feasibility of this ISC pathway, we optimized the geometries of the *S*_1_ and *S*_2_ states using TDDFT and *T*_1_ state using unrestricted DFT (see Supporting Section S1).

The optimized bond lengths in the ground-state geometries of the complexes are *r*(Ru–Ru) = 2.84 Å and *r*(Fe–Fe) = 2.74 Å, with a planar torsional angle ϕ­(M–C–C–M) = 0.0° (M = Ru, Fe). TDDFT optimization of the *S*_1_ and *S*_2_ states yields only minor changes in these parameters, with RMSD < 0.1 Å for both (Fv)­Ru_2_(CO)_4_ and (Fv)­Fe_2_(CO)_4_ and the torsional angle ϕ remains near zero. This structural rigidity is consistent with π_⊥_^*^/π_∥_^*^ → σ* excitations, which impart minimal perturbation to the metal–metal bond order. In contrast, the optimized *T*_1_ states (*syn* Triplet, Scheme 1B for Ru), henceforth referred to as *syn*-*T*_1_, exhibit significant bond elongation [*r*(Ru–Ru) = 4.21 Å, *r*(Fe–Fe) = 3.87 Å] and increased dihedral angles ϕ (40.9° in Ru_2_ and 27.9° in Fe_2_). These changes arise from σ → σ* excitations, which considerably weaken the metal–metal bond and generate a dissociative *T*_1_ PES. The corresponding structures are illustrated in Supporting Section S1.

Reminiscent of recent studies that linked excited-state lifetimes with ligand-field strengths in first-row transition-metal complexes,^51,52^ the correlation between ISC rates and ligand-field strength in (Fv)­M_2_(CO)_4_ (M = Fe, Ru, Os) can be rationalized using semiclassical Marcus theory.^53,54^ Although originally developed for electron-transfer reactions, the principles of Marcus theory can be applied to a wide range of photophysical processes, including nonradiative ISC. As such, this process is influenced by the energy gap, nuclear reorganization, and the coupling between the involved spin states (i.e. SOC). The semiclassical form treats electronic transitions quantum mechanically via Fermi’s golden rule,^55^ while nuclear motion is treated classically. Accordingly, the ISC rate *k*_ISC_ within semiclassical Marcus theory^53,54^ is determined by two factors (Figure 2A): (i) the SOC matrix element (*H*_SO_) between the involved singlet and triplet states, evaluated at the crossing-point geometry, and (ii) and the activation energy barrier for ISC, Δ*E* ^‡^, calculated as Δ*E* ^‡^ = (Δ*E* + λ)^2^/4λ. Here, Δ*E* represents the energy difference between the excited singlet and triplet minima, while λ is the average of the singlet and triplet reorganization energies, expressed as λ = (^1^λ + ^3^λ)/2. In this work, only the inner-sphere component of the reorganization energy is considered. This approximation is justified by the fact that the excited states are metal-centered and that solvent stabilization effects are negligible (<0.1 eV, see Supporting Section S3 and Section S6).

**Figure 2:**
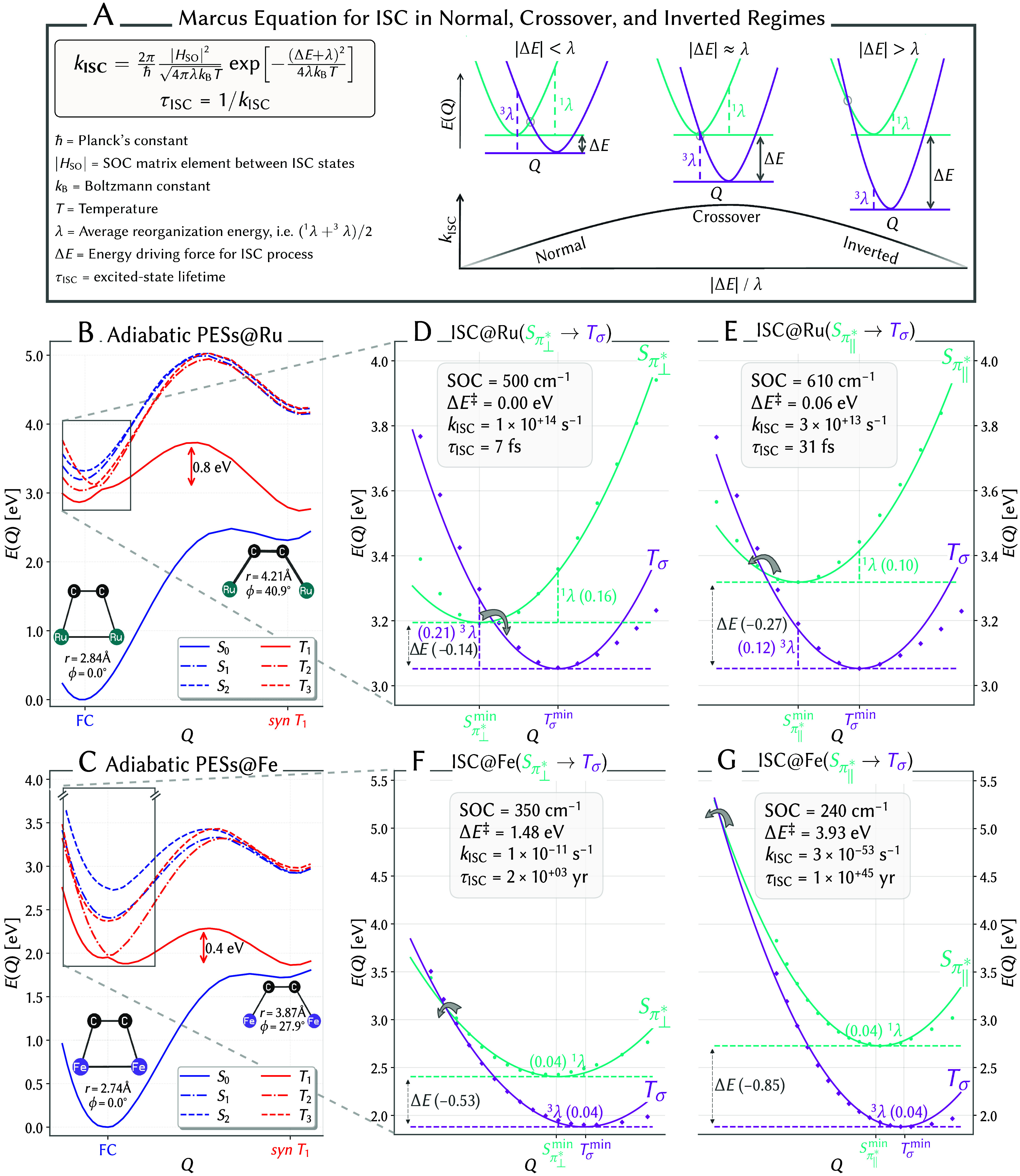
Intersystem crossing (ISC) rates for (Fv)­Ru_2_(CO)_4_ and (Fv)­Fe_2_(CO)_4_. (A) Marcus rate equation and schematic representation of the variation in ISC rate (*k*_ISC_) across different Marcus regimes. (B, C) Adiabatic energy profiles of (Fv)­Ru_2_(CO)_4_ (B) and (Fv)­Fe_2_(CO)_4_ (C) from the FC point to the *syn*-*T*_1_ minimum. (D–G) Insets of the quadratically fitted diabatic energy profiles for the *S*_π_⊥_^*^_ → *T*_σ_ (D) and *S*_π_∥_^*^_ → *T*_σ_ (E) potentials relevant for intersystem crossing in the Ru_2_ complex and the counterparts, *S*_π_⊥_^*^_ → *T*_σ_ (F) and *S*_π_∥_^*^_ → *T*_σ_ (G) in the Fe_2_ complex. Values in parentheses next to Δ*E*, ^1^λ, and ^3^λ are in eV. Singlet and triplet adiabatic surfaces are blue and red, respectively; diabatic surfaces are cyan and magenta.

Depending on the singlet–triplet energy gap and in analogy with the electron-transfer reactions described by Marcus theory, we define three state-crossing Marcus regimes: the normal regime (λ > |Δ*E*|), the crossover regime (λ ≈ |Δ*E*|), and the inverted regime (λ < |Δ*E*|) (Figure 2A). Within this framework, the rate constant for ISC, *k*_ISC_, is expected to increase gradually as the system moves from the normal to the crossover regime due to a decreasing activation barrier (Δ*E* ^‡^), reaching a maximum at the barrierless crossover regime. Conversely, in the inverted regime, *k*_ISC_ decreases progressively with increasing activation barrier despite the process becoming more exothermic.

The activation barrier Δ*E* ^‡^ and reorganization energy λ parameters were obtained by diabatizing the adiabatic (i.e., energy-ordered) PESs of the two lowest excited singlet states (*S*_1_, *S*_2_) and the three lowest triplet states (*T*_1_, *T*_2_, *T*_3_) along the reaction coordinate *Q*, defined as a linear interpolation between the Franck–Condon (FC) and the respective *syn*-*T*_1_ geometries. Since *Q* involves substantial bond elongation and bond cleavage, single-reference methods such as DFT and TDDFT are expected to be inadequate for an accurate description of the full PESs. Accordingly, extended multistate complete active space second-order perturbation theory (XMS-CASPT2) computations were performed to generate the full adiabatic PESs, see Figure 2B,C.

The diabatic (ordered by the nature of the electronic transition) PESs obtained from the five states (*S*_1_, *S*_2_, and *T*_1_, *T*_2_, *T*_3_) are provided in Supporting Section S8. For the Ru_2_ complex, Figure 2 panels D and E, and for the Fe_2_ complex, panels F and G, show a zoomed-in view of the pair of diabatic singlet and triplet states (cyan and magenta) that are central to the ISC process driving photoisomerization. The diabatic curves correspond to the ^3^[(σ)^1^(σ*)^1^] triplet state and the ^1^[(π_⊥_^*^)^1^(σ*)^1^] and ^1^[(π_∥_^*^)^1^(σ*)^1^] excited singlet states, which are bright at the equilibrium geometry for both complexes. For brevity, these states are referred to as *T*_σ_, *S*_π_⊥_^*^_, and *S*_π_∥_^*^_, respectively. This notation reflects the donor orbital in the excitation, as all states share a common σ* acceptor, enabling concise and chemically meaningful labeling. In the diabatic picture, both *S*_π_⊥_^*^_ and *S*_π_∥_^*^_ exhibit single-well potentials with minima near the FC geometry, denoted as *S*_π_⊥_^*^_^min^ and *S*_π_∥_^*^_^min^, respectively. In contrast, the *T*_σ_ triplet state displays a double-well potential along the interpolated reaction coordinate *Q*, featuring two distinct minima: one near the FC geometry (*T*_σ_^min^, labeled in Figure 2D–G), and the other at the relaxed *syn*-*T*_1_ geometry. These minima correspond to local and global conformers of the diabatic *T*_σ_ state, separated by an activation barrier of 0.8 eV for the Ru complex (Figure 2B) and 0.4 eV for the Fe complex (Figure 2C). The diabatic surfaces were fitted near the FC geometry using a one-dimensional harmonic oscillator model.

For (Fv)­Ru_2_(CO)_4_, the crossing between the *S*_π_⊥_^*^_ and *T*_σ_ diabatic PESs occurs near the *S*_π_⊥_^*^_^min^ geometry (Figure 2D), with a SOC matrix element of ⟨*S*_π_⊥_^*^_|*H*_SO_|*T*_σ_⟩ = 500 cm^–1^. The corresponding driving force (Δ*E*) and reorganization energy (λ) are −0.14 and 0.18 eV, respectively, resulting in a barrierless transition (Δ*E* ^‡^ = 0.00 eV). The ISC rate constant is *k*_ISC_ = 1 × 10^14^ s^–1^, corresponding to a lifetime of τ_ISC_ = 7 fs (Figure 2D). A similar crossing between *S*_π_∥_^*^_ and *T*_σ_ occurs near the *S*_π_∥_^*^_^min^ geometry (Figure 2E), with a SOC matrix element, ⟨*S*_π_∥_^*^_|*H*_SO_|*T*_σ_⟩ = 610 cm^–1^. Here, Δ*E* = −0.27 eV and λ = 0.11 eV, resulting in a nearly barrierless ISC (Δ*E* ^‡^ = 0.06 eV) with *k*_ISC_ = 3 × 10^13^ s^–1^ and τ_ISC_ = 31 fs (Figure 2E). Although the obtained time scales might be approximate due to the associated error of the XMS-CASPT2 calculations and the fitting, the ultrafast nature of this ISC is in line with previously calculated^56,57^ and measured time scales for other Ru complexes.^58–61^

The situation for (Fv)­Fe_2_(CO)_4_ is dramatically different. The crossing between the *S*_π_⊥_^*^_ and *T*_σ_ diabatic PESs occurs significantly to the left of the *S*_π_⊥_^*^_^min^ minimum (Figure 2F). Thus, despite the SOC matrix element ⟨*S*_π_⊥_^*^_|*H*_SO_|*T*_σ_⟩ is 350 cm^–1^—only slightly smaller than that of the Ru_2_ complex—the estimated Δ*E* is more exothermic (−0.53 eV), while λ is significantly smaller (0.04 eV), leading to a substantial activation energy barrier Δ*E* ^‡^ = 1.48 eV. This results in an extremely low *k*_ISC_ of 1 × 10^–11^ s^–1^ and a correspondingly long τ_ISC_ on the order of years (Figure 2F). Likewise, the crossing between *S*_π_∥_^*^_ and *T*_σ_ occurs further to the left of the *S*_π_∥_^*^_^min^ minimum (Figure 2G). The SOC matrix element ⟨*S*_π_∥_^*^_|*H*_SO_|*T*_σ_⟩ remains at 240 cm^–1^, while Δ*E* is −0.85 eV and λ stays at 0.04 eV. However, Δ*E* ^‡^ surges to 3.93 eV, resulting in an extraordinarily low *k*_ISC_ of 3 × 10^–53^ s^–1^ and an effectively eternal τ_ISC_ lifetime (Figure 2G).

Our calculations reveal that the Ru_2_ complex undergoes ultrafast ISC (7–31 fs), enabling efficient triplet biradical formation, while the Fe_2_ analogue exhibits ISC on the order of thousands of years—consistent with the experimental absence of a triplet intermediate. A prior transient IR study on the Ru_2_ complex reported a 30 ps decay component, associated with species exhibiting IR bands at 1945, 1995, and 2010 cm^–1^.^7^ This slower time scale likely reflects structural or vibrational relaxation within the triplet manifold, not the ISC process itself. Our findings thus establish prompt ISC as a key mechanistic feature distinguishing the Ru_2_ system.

According to El-Sayed’s rule,^62^ SOC is enhanced when singlet and triplet states differ in orbital character, as a change in spin angular momentum necessitates a corresponding change in orbital angular momentum to conserve total angular momentum. Both the Ru_2_ and Fe_2_ complexes exhibit significant ⟨*S*_π_⊥_^*^_|*H*_SO_|*T*_σ_⟩ and ⟨*S*_π_∥_^*^_|*H*_SO_|*T*_σ_⟩ SOC matrix elements, due to changes in orbital character. However, it is the ISC activation energy barrier, Δ*E* ^‡^, which ultimately governs the ISC rates. In the Ru_2_ complex, ISC occurs within the crossover regime (λ ≈ |Δ*E*|), facilitating the rapid and barrierless formation of the triplet intermediate (Figure 2C,D). In contrast, the weak ligand field in the Fe_2_ complex stabilizes the triplet-state energies, satisfying λ ≪ |Δ*E*| for ISC from both bright singlet states (Figure 2F,G), shifting ISC deep into the inverted regime and suppressing ISC. This result underscores that SOC magnitude alone is insufficient to rationalize ISC kinetics. Particularly in the first-row transition- metal complexes, the ISC efficiency is controlled by activation barriers, making it essential to move beyond SOC-based arguments toward frameworks that account for surface-crossing energetics.

To complete trends in the Group-8 of the periodic table, we have calculated the *k*_ISC_ in the (Fv)­Os_2_(CO)_4_ complex (Supporting Section S9). As in the Ru_2_ complex, the favorable excited-state energetics and near-barrierless crossings in the Marcus boundary regime lead to ultrafast ISC rates up to 9 × 10^14^ s^–1^ (τ_ISC_ = 1 fs). Although strong SOC (>1400 cm^–1^) aids ISC, it is the low activation energy that dominates the kinetics. However, its higher thermal back-reaction barrier (1.76 eV vs 1.11 eV for (Fv)­Ru_2_(CO)_4_) prevents reversible photoisomerization, limiting MOST applicability.^8,10,14^

We note that even if (Fv)­Fe_2_(CO)_4_ were hypothetically endowed with the large SOC of (Fv)­Os_2_(CO)_4_ (1423 cm^–1^), *k*_ISC_ would increase only from 1 × 10^–11^ to 2 × 10^–10^ s^–1^, since SOC enters the rate solely as a prefactor. The hybrid (Fv)­RuFe­(CO)_4_ complex (Supporting Section S10), despite SOC strengths comparable to (Fv)­Ru_2_(CO)_4_ (>400 cm^–1^) is also photoinert due to a Marcus-inverted singlet–triplet landscape with a large energy gap (>0.35 eV), akin to (Fv)­Fe_2_(CO)_4_.^10,12^ This example shows again that ISC kinetics are predominantly governed by Marcus surface-crossing topologies.

Finally, we note that the ISC process have been analyzed using semiclassical Marcus theory,^53,54^ which is claimed to underestimate rates in the inverted regime.^63^ To account for vibronic effects relevant at room temperature, one can use the Marcus–Levich–Jortner (MLJ) theory, which incorporates quantized nuclear vibrations,^64^ capturing nuclear quantum effects that may be significant in the inverted regime.^63,65^ Applying MLJ confirms distinct ISC mechanisms in the (Fv)­M_2_(CO)_4_ (M = Ru, Fe, Os) series (Supporting Section S11). Ru_2_ and Os_2_ complexes lie near the Marcus boundary regime (|Δ*E*| ≈ λ), with reorganization dominated by strong coupling to low-frequency metal–metal stretching modes (<600 cm^–1^), which are partially thermally populated at room temperature and can be treated as semiclassical vibrations. In this regime, semiclassical Marcus theory^53,54^ accurately captures femtosecond-scale ISC rates, with MLJ providing only minor corrections. In contrast, the Fe_2_ complex lies deep in the inverted regime (|Δ*E*| ≫ λ), where reorganization involves weakly coupled, anharmonic low-frequency modes, with negligible contributions from medium- or high-frequency modes. As a result, quantum vibrational activation is ineffective, and the MLJ model fails due to broken assumptions of harmonicity and mode separability.

### Relaxation Dynamics from the Triplet Intermediate in (Fv)­Ru_2_(CO)_4_

Once the *syn*-*T*_1_ biradical intermediate is formed in (Fv)­Ru_2_(CO)_4_, it can nonradiatively decay back to the ground-state via reverse ISC (rISC: *T*_1_ → *S*_0_), reforming the Ru–Ru bond, before undergoing the *syn*–to–*anti* torsional rotation to drive photoisomerization. A small *k*_rISC_ is beneficial to prolong the triplet biradical lifetime, favoring photoisomerization over Ru–Ru bond reformation. This requires both the pre-exponential SOC term, ⟨*T*_1_|*H*_SO_|*S*_0_⟩, and the exponential factor, exp­[−Δ*E*_rISC_^‡^/(*k*_B_ *T*)], to be as small as possible. The latter necessitates a high rISC activation barrier, Δ*E*_rISC_^‡^, on the *T*_1_ surface. At the *syn*-*T*_1_ geometry (ϕ ≈ 41°), the *T*_1_ state is 0.33 eV above *S*_0_ (Figure 3). As ϕ increases toward the *anti*-*T*_1_ conformer (*anti* Triplet, Scheme 1), the *T*_1_ and *S*_0_ states converge and cross at ϕ ≈ 95° (^1,3^MECP*_syn‑anti_*), where the energy gap vanishes. The associated activation barrier on the *T*_1_ surface, Δ*E*_rISC_^‡^, is minimal (0.06 eV), which should facilitate efficient *T*_1_ → *S*_0_ rISC. However, rISC does not occur because, despite a large SOC matrix element between the *T*_1_ and *S*_0_ states (⟨*T*_1_|*H*_SO_|*S*_0_⟩ ∼ 350 cm^–1^) at the FC geometry, the SOC rapidly diminishes along the interpolated *Q* coordinate, dropping to 50 cm^–1^ at the *syn*-*T*_1_ geometry (Supporting Section S12). Further, as the two states converge along the ϕ coordinate from *syn*-*T*_1_ to *anti*-*T*_1_, the ⟨*T*_1_|*H*_SO_|*S*_0_⟩ matrix element vanishes at the energetically degenerate ^1,3^MECP*_syn‑anti_* geometry (Figure 3). Notably, at the *anti*-*T*_1_ geometry (ϕ ≈ 178°), the SOC remains close to zero, effectively preventing rISC (*T*_1_ → *S*_0_), and thus promoting *syn*–to–*anti* isomerization.

**Figure 3:**
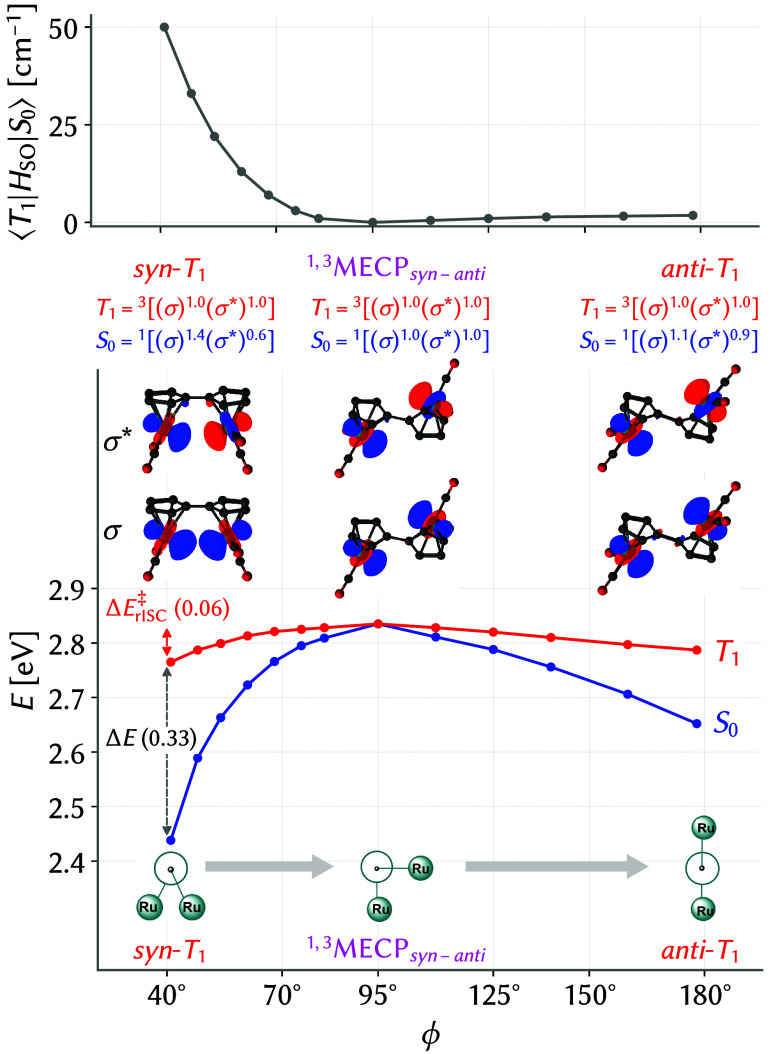
Stability of the triplet biradical intermediate in (Fv)­Ru_2_(CO)_4_ along the *syn*–to–*anti* dihedral (ϕ) coordinate. Adiabatic PESs for the ground-state singlet (*S*_0_, blue) and lowest triplet state (*T*_1_, red), linking *syn*-*T*_1_ and *anti*-*T*_1_ geometries via the minimum-energy crossing point (^1,3^MECP*_syn‑anti_*) (bottom). Molecular orbitals (σ and σ*) and their occupations are shown for these conformations, emphasizing electronic rearrangements along the ϕ coordinate. The figure also depicts the variation in SOC matrix elements along the dihedral (ϕ) coordinate (top).

The dramatic decrease in SOC can be explained with the El-Sayed’s rule,^62^ which depends on the orbital character of the states involved. The *S*_0_ state evolves from the ^1^[(σ)^2^(σ*)^0^] configuration at the FC geometry to the ^1^[(σ)^1.4^(σ*)^0.6^] configuration at the *syn*-*T*_1_ geometry (Supporting Section S12) followed by a transition to the ^1^[(σ)^1^(σ*)^1^] configuration at the ^1,3^MECP*_syn‑anti_* geometry, closely resembling the *T*_1_ state, ^3^[(σ)^1^(σ*)^1^] (Figure 3). This change in orbital character of *S*_0_ leads to a vanishing ⟨*T*_1_|*H*_SO_|*S*_0_⟩. Consequently, although the activation barrier for rISC (*T*_1_ → *S*_0_), Δ*E*_rISC_^‡^, is small (0.06 eV), the zero SOC at ^1,3^MECP*_syn‑anti_* inhibits rISC to *S*_0_, extending the *T*_1_ lifetime, preventing Ru–Ru bond reformation, and thereby facilitating *syn*–to–*anti* isomerization. Once in the *anti*-*T*_1_ configuration, the formation of the photoproduct involves C–C bond cleavage, disrupting both σ and π bonds while forming two Ru–C σ-bonds. According to Kanai et al.^8^ the barrier for this concerted step is about 0.19 eV. This step alters the geometry and electronic structure, converting the *S*_0_ state from open-shell back to closed-shell. A second minimum-energy crossing point (^1,3^MECP_final_) is expected during this rearrangement, where different orbital occupations of *S*_0_ and *T*_1_ states enhance SOC according to El-Sayed’s rule.^62^ This finally leads to nonradiative rISC from *T*_1_ to *S*_0_ with a significant rate constant, yielding the singlet photoproduct (see the orbital correlation diagram in Supporting Section S13).

In (Fv)­Fe_2_(CO)_4_, the first ISC is kinetically hindered—both experimentally and computationally due to the presence of Marcus-inverted behavior. Nonetheless, we explored the hypothetical scenario in which the triplet manifold were to be accessed, beginning from the *syn*-*T*_1_ biradical state (Supporting Section S14). On this surface, the system would undergo a low-barrier torsional rotation followed by rearrangement, closely paralleling the behavior of its Ru_2_ analogue. Critically, SOC diminishes near MECP, effectively suppressing rISC and enabling efficient photoisomerization along the triplet pathway. These features collectively indicate that, although the first ISC remains the principal bottleneck in the Fe_2_ system, if it were overcome through suitable ligand substitution, downstream reactivity on the triplet surface would be both energetically feasible and mechanistically robust.

### Wavelength-Dependent Decarbonylation Mechanisms

To conclude our mechanistic investigation, we present a rationale for the observed wavelength-dependent decarbonylation reactivity in the Ru_2_ and Fe_2_ complexes. TDDFT (Supporting Section S3) and XMS-CASPT2 (Supporting Section S4) calculations consistently assign the higher-energy excitations—region IV in Ru_2_ and region II in Fe_2_—to transitions involving metal-centered δ or δ* orbitals.

The metal *d*_δ_ orbitals align along the M–CO σ bonds, allowing for symmetry-allowed interaction with the σ-donor lone pairs primarily centered on the carbon atoms of the CO ligands. As described by ligand field theory, at each metal center, the σ orbitals of the two CO ligands first combine to form ligand group orbitals: the in-phase (+) bonding combination, denoted CO­(σ*), and the out-of-phase (−) antibonding combination, denoted CO­(σ*). Due to symmetry constraints, only the CO­(σ*) can effectively mix with the metal *d*_δ_ orbitals.

This interaction yields two new molecular orbitals at each metal center: a bonding combination, (M–CO­(σ*))_+_, with dominant CO­(σ*) character, and an antibonding combination, (M–CO­(σ*))_−_, with dominant metal *d*_δ_ character. Quantitative molecular orbital decomposition for (Fv)­M_2_(CO)_4_ (M = Ru, Fe), presented in Figure 4, further elucidates the nature of these orbitals. For (Fv)­Ru_2_(CO)_4_, the bonding orbital (Ru­(d_δ_) - CO­(σ*))_+_ comprises approximately 20% Ru­(4*d*_δ_) and 58% CO, indicating a primarily CO ligand-centered character. The antibonding orbital (Ru­(*d*_δ_) - CO­(σ*))_−_ features about 54% Ru­(4*d*_δ_), 15% CO, and 12% fulvalene ligand contributions. Similarly, for (Fv)­Fe_2_(CO)_4_, the bonding orbital is composed of roughly 6% Fe­(3*d*_δ_) and 82% CO ligand contributions, while the antibonding orbital has about 73% Fe­(3*d*_δ_) and 8% CO character.

**Figure 4:**
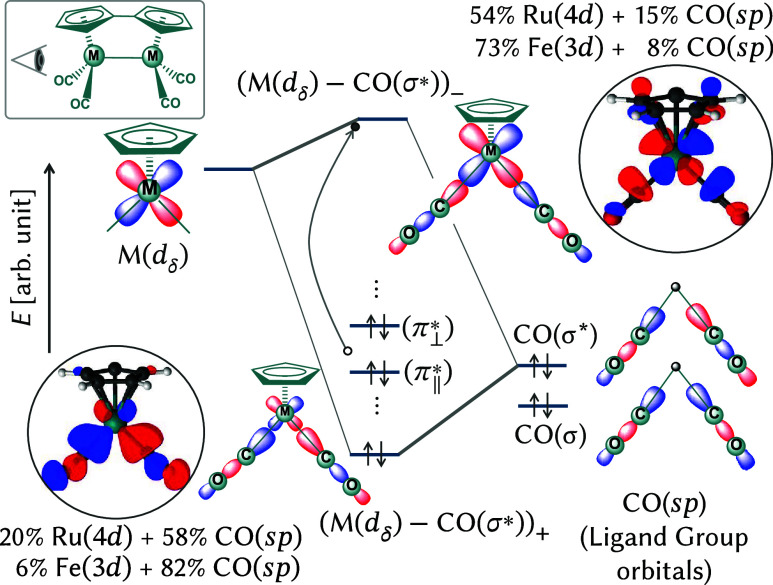
Orbital diagram illustrating M–CO interactions in decarbonylation. A qualitative orbital energy diagram illustrates interactions between M (*d*_δ_) orbitals (M = Ru, Fe) and CO (σ) ligand group orbitals, showing in-phase (“+’’, bonding) and out-of-phase (“*–*’’, antibonding) interactions. Ground-state orbital isosurfaces (±0.4, inset) highlight relevant orbitals and fragment contributions from the (XMS)–CASPT2­(10*e*,14o) calculation. The inset provides the molecular view perspective to clarify the orbital interactions.

These decompositions underscore the ligand-assisted nature of the δ/δ* states and the pronounced metal vs ligand orbital character difference between bonding and antibonding orbitals. As illustrated in Figure 4, the antibonding orbital is energetically destabilized, particularly in Ru_2_, where it becomes accessible under UV irradiation (<300 nm). In Fe_2_, which experiences a weaker ligand field, the corresponding excitations occur at lower energies (450–500 nm), consistent with visible light absorption.

Notably, these excitations originate from metal–metal antibonding π* orbitals, which serve as the antibonding counterparts of the strongly overlapping π bonding orbitals present in the metal dimers. Therefore, π* → δ/δ* excitations not only weaken the M–CO bonds by populating antibonding orbitals but also strengthen the metal–metal bond by depopulating the metal–metal antibonding π* levels. This dual effect facilitates decarbonylation under light irradiation at appropriate wavelengths. These results support a ligand- and symmetry-assisted orbital mechanism for photodecarbonylation that is sensitive to both excitation wavelength and metal identity.

## Conclusions

In summary, we report a mechanistic account of the different excited-state photochemistry in the fulvalene-bridged Group-8 bimetallic (Fv)­M_2_(CO)_4_ (M_2_ = Fe_2_, Ru_2_, Os_2_ and mixed RuFe) molecular solar–thermal (MOST) systems. Using multiconfigurational quantum chemistry, we map the excited-state potential energy surfaces, quantify spin–orbit coupling (SOC) elements, and model intersystem crossing (ISC) pathways to explain the contrasting relaxation mechanisms of (Fv)­Ru_2_CO_4_ and (Fv)­Fe_2_CO_4_. These are illustrated in Figure 5, which summarizes both energetically accessible (solid arrows) and hypothetical (dashed arrows) reaction pathways. In the Fe_2_ complex (Figure 5A), all excited states are red-shifted, lowering the threshold for metal-centered π* → δ/δ* transitions that promote decarbonylation above 350 nm. In contrast, the Ru_2_ complex (Figure 5B) loses CO only below 300 nm. Photoisomerization requires excitation to metal-centered π*−σ* states, which can undergo ISC to a triplet state with σ–σ* character. These states become accessible above 350 nm in the Ru_2_ complex, but shift into the visible region, above 450 nm, in the Fe_2_ one.

**Figure 5:**
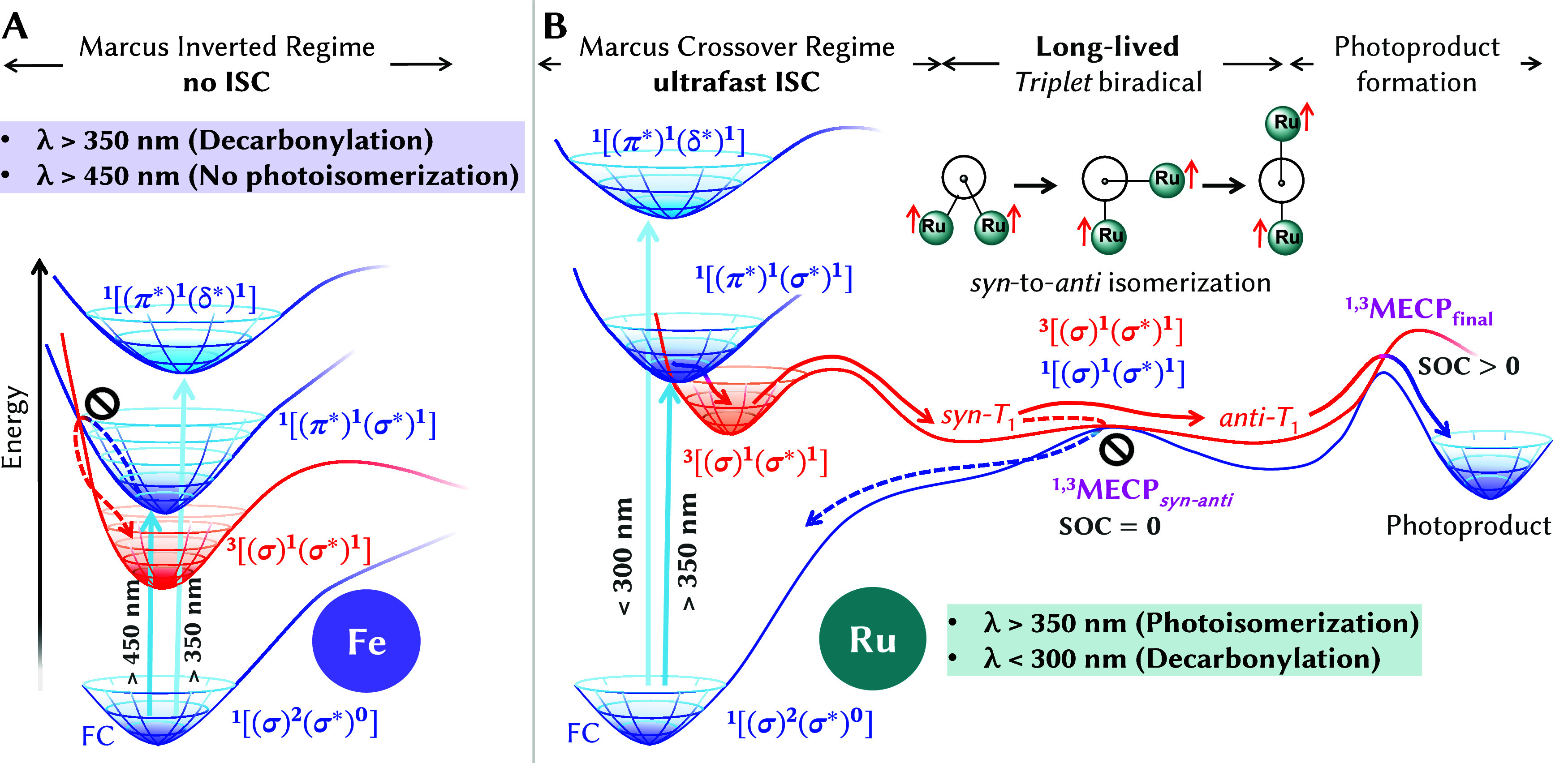
Photochemical Pathways for (Fv)­M_2_(CO)_4_, M = Ru, Fe complexes. (A) In the Fe_2_ complex, intersystem crossing (ISC) occurs in the Marcus-inverted regime, leading to decarbonylation. (B) In the Ru_2_ complex, barrierless ISC in the Marcus crossover regime leads to a ^3^[(σ)^1^(σ*)^1^] triplet biradical formation; this exhibits a long lifetime due to zero SOC with the singlet ground state at the crossing point ^1,3^MECP*_syn‑anti_*, ultimately enabling *syn*-to-*anti* photoisomerization. Enhanced SOC at ^1,3^MECP_final_ facilitates photoproduct formation.

The efficiency of ISC is in general determined by the SOC and the singlet–triplet energy. We find that the Ru_2_-complex undergoes nearly barrierless ISC in the Marcus crossover regime (Figure 5B), whereas the Fe_2_ analogue encounters a substantial activation barrier in the Marcus-inverted regime (Figure 5A), suppressing triplet biradical formation. Importantly, it is not SOC alone, but the position of the singlet/triplet crossing within the Marcus regime what determines ISC feasibility and thus the onset of photoisomerization. Notably, this mechanism can be extended to the entire group 8 of the periodic table. (Fv)­Os_2_(CO)_4_ also undergoes ultrafast ISC as (Fv)­Ru_2_(CO)_4_ because the crossing lies in the Marcus boundary regime. In contrast, the (Fv)­RuFe­(CO)_4_ mixed compound remains photoinactive despite its intermediate SOC, mirroring the behavior of (Fv)­Fe_2_(CO)_4_ in the Marcus-inverted regime. Even an artificial enhancement of the SOC’s magnitude of (Fv)­Fe_2_(CO)_4_ to that of (Fv)­Os_2_(CO)_4_ fails to facilitate ISC, reinforcing the idea that it is not the SOC, but the position of the singlet–triplet crossing in the Marcus regime which ultimately governs ISC efficiency in these systems. The absence of photoswitching in (Fv)­Fe_2_(CO)_4_ originates solely from Marcus-inverted-regime ISC suppression, as subsequent biradical steps remain kinetically accessible. This distinction reframes prior assumptions in metal-based MOST systems. The further stabilizing factors in the Ru_2_ complex include negligible SOC with the singlet ground state and enhanced SOC between orbitals following C–C bond cleavage and Ru–C bond formation. This facilitates *syn-*to-*anti* isomerization and suppresses recombination (dashed arrow in Figure 5B), ultimately enabling photoproduct formation.

A vibronic coupling analysis within the Marcus theory further reveals that Franck–Condon overlaps and specific vibrational modes modulate ISC efficiency, explaining how similar electronic configurations lead to distinct photoreactivity across the series.

From these insights, three key design principles emerge for engineering efficient bimetallic MOST systems: (i) avoid Marcus-inverted regimes (|Δ*E*| > λ), where excessive driving force suppresses ISC irrespective of SOC strength; (ii) tune ligand fields—via targeted ligand substitutions or tailored metal combinations—to shift systems from the Marcus-inverted toward the crossover (boundary) regime (|Δ*E*| ≈ λ), thereby maximizing ISC efficiency; and (iii) balance SOC and energetic alignment in heterobimetallic architectures, where SOC enhancement from heavy atoms must be weighed against possible energetic mismatch introduced by lighter partners. These principles provide experimentally accessible chemical handles for tuning ligand fields and metal centers, offering a concise and general roadmap for designing complexes with efficient photoisomerization and optimized ISC dynamics.

This study thus advances the mechanistic understanding of photoinduced processes in bimetallic complexes by elucidating how the Ru-based triplet intermediate forms and how spin-density localization on the metal centers contributes to its stabilization. The validated mechanistic framework—spanning Group-8 homo- (Fe_2_, Ru_2_, and Os_2_) and heterobimetallic (RuFe) systems—extends beyond traditional El-Sayed rules by providing a predictive model for the design of photoactive complexes. It further clarifies the electronic origins of competing photodecarbonylation pathways and establishes a theoretical foundation for rationally controlling photoreactivity in related systems.

## Supplementary Material



## Data Availability

Additional data are available from the corresponding authors upon reasonable request.

## Notes

Computational details for (Fv)­M_2_(CO)_4_ complexes (M = Fe, Ru, Os), including DFT-optimized structures and Cartesian coordinates; molecular orbital and excited-state analyses using natural transition orbitals; XMS-CASPT2 spectral and state character analyses; benchmarking of ionization potential-electron affinity values; spin–orbit coupling calculations and diabatic potential energy surfaces; and mechanistic studies of intersystem crossing, reverse intersystem crossing, and relaxation (PDF)

## References

[1] Forster L. S. (2006). Intersystem
Crossing in Transition Metal Complexes. Coord.
Chem. Rev..

[2] McCusker J. K. (2019). Electronic
Structure in the Transition Metal Block and Its Implications for Light
Harvesting. Science.

[3] Hernández-Castillo D., Eder I., González L. (2024). Guidelines to Calculate Non-Radiative
Deactivation Mechanisms of Ruthenium Tris­(bipyridine) Derivatives. Coord. Chem. Rev..

[4] Zhang W., Alonso-Mori R., Bergmann U., Bressler C., Chollet M., Galler A., Gawelda W., Hadt R. G., Hartsock R. W., Kroll T. (2014). Tracking Excited-State
Charge and Spin Dynamics in
Iron Coordination Complexes. Nature.

[5] Liu Y., Persson P., Sundstrom V., Warnmark K. (2016). Fe N-Heterocyclic Carbene
Complexes as Promising Photosensitizers. Acc.
Chem. Res..

[6] Boese R., Cammack J. K., Matzger A. J., Pflug K., Tolman W. B., Vollhardt K. P. C., Weidman T. W. (1997). Photochemistry of (Fulvalene)­tetracarbonyldiruthenium
and Its Derivatives: Efficient Light Energy Storage Devices. J. Am. Chem. Soc..

[7] Harpham M. R., Nguyen S. C., Hou Z. (2012). X-ray Transient Absorption
and Picosecond IR Spectroscopy of Fulvalene­(tetracarbonyl)­diruthenium
on Photoexcitation. Angew. Chem., Int. Ed..

[8] Kanai Y., Srinivasan V., Meier S. K., Vollhardt K. P. C., Grossman J. C. (2010). Mechanism of Thermal
Reversal of the (Fulvalene)­tetracarbonyldiruthenium
Photoisomerization: Toward Molecular Solar-Thermal Energy Storage. Angew. Chem., Int. Ed..

[9] Moth-Poulsen K., Ćoso D., Börjesson K., Vinokurov N., Meier S. K., Majumdar A., Vollhardt K. P. C., Segalman R. A. (2012). Molecular Solar Thermal (MOST) Energy Storage and Release
System. Energy Environ. Sci..

[10] Börjesson K., Ćoso D., Gray V., Grossman J. C., Guan J., Harris C. B., Hertkorn N., Hou Z., Kanai Y., Lee D. (2014). Exploring the Potential of Fulvalene Dimetals as Platforms
for Molecular Solar Thermal Energy Storage: Computations, Syntheses,
Structures, Kinetics, and Catalysis. Chem.-Eur.
J..

[11] Lennartson A., Lundin A., Börjesson K., Gray V., Moth-Poulsen K. (2016). Tuning the
Photochemical Properties of the Fulvalene-tetracarbonyl-diruthenium
System. Dalton Trans..

[12] Hou Z., Nguyen S. C., Lomont J. P., Harris C. B., Vinokurov N., Vollhardt K. P. C. (2013). Switching
from Ru to Fe: Picosecond IR Spectroscopic
Investigation of the Potential of the (Fulvalene) Tetracarbonyldiiron
Frame for Molecular Solar-Thermal Storage. Phys.
Chem. Chem. Phys..

[13] Kahn A. P., Boese R., Blümel J., Vollhardt K. P. C. (1994). Synthesis
and chemistry of heterobimetallic fulvalene complexes containing W,
MO, and Rh. J. Organomet. Chem..

[14] Zhu B., Miljanić O. Š., Vollhardt K. P. C., West M. J. (2005). Synthesis of 2,2’,3,3′-tetramethyl-
and
2,2’,3,3′-tetra-tert-butylfulvalene: Attractive platforms
for dinuclear transition metal fragments, as exemplified by (*η*
^5^: *η*
^5^-2,2’,3,3′-t-Bu_4_C_10_H_4_)­M_2_(CO) _
*n*
_ (M = Fe, Ru, Os,
Mo) and first X-ray crystal structures of fulvalene diiron and diosmium
complexes. Synthesis.

[15] Drage J. S., Vollhardt K. P. C. (1986). The
Chemistry of (Fulvalene)­dimolybdenum Hexacarbonyl:
A Rigidly Held Dinuclear Transition-Metal Complex. Organometallics.

[16] Marian C. M. (2012). Spin–Orbit
Coupling and Intersystem Crossing in Molecules. WIREs Comput. Mol. Sci..

[17] Lomont J. P., Nguyen S. C., Harris C. B. (2014). Ultrafast Infrared Studies of the
Role of Spin States in Organometallic Reaction Dynamics. Acc. Chem. Res..

[18] Kucharski T. J., Ferralis N., Kolpak A. M., Zheng J. O., Nocera D. G., Grossman J. C. (2014). Templated Assembly of Photoswitches Significantly Increases
the Energy-Storage Capacity of Solar Thermal Fuels. Nat. Chem..

[19] Liu Y., Grossman J. C. (2014). Accelerating the Design of Solar Thermal Fuel Materials
Through High Throughput Simulations. Nano Lett..

[20] Wang Z., Udmark J., Börjesson K., Rodrigues R., Roffey A., Abrahamsson M., Nielsen M. B., Moth-Poulsen K. (2017). Evaluating
Dihydroazulene/Vinylheptafulvene Photoswitches for Solar Energy Storage
Applications. ChemSusChem.

[21] Dreos A., Börjesson K., Wang Z., Roffey A., Norwood Z., Kushnir D., Moth-Poulsen K. (2017). Exploring the Potential of a Hybrid
Device Combining Solar Water Heating and Molecular Solar Thermal Energy
Storage. Energy Environ. Sci..

[22] Dong L., Feng Y., Wang L., Feng W. (2018). Azobenzene-Based Solar
Thermal Fuels: Design, Properties, and Applications. Chem. Soc. Rev..

[23] Mansø M., Petersen A. U., Wang Z., Erhart P., Nielsen M. B., Moth-Poulsen K. (2018). Molecular Solar Thermal Energy Storage in Photoswitch
Oligomers Increases Energy Densities and Storage Times. Nat. Commun..

[24] Ganguly G., Sultana M., Paul A. (2018). Designing
Efficient Solar-Thermal
Fuels with [*n.n*]­(9,10)­Anthracene Cyclophanes: A Theoretical
Perspective. J. Phys. Chem. Lett..

[25] Wang Z., Roffey A., Losantos R., Lennartson A., Jevric M., Petersen A. U., Quant M., Dreos A., Wen X., Sampedro D., Börjesson K., Moth-Poulsen K. (2019). Macroscopic
Heat Release in a Molecular Solar Thermal Energy Storage System. Energy Environ. Sci..

[26] Orrego-Hernández J., Dreos A., Moth-Poulsen K. (2020). Engineering of Norbornadiene/Quadricyclane
Photoswitches for Molecular Solar Thermal Energy Storage Applications. Acc. Chem. Res..

[27] Wang Z., Erhart P., Li T., Zhang Z.-Y., Sampedro D., Hu Z., Wegner H. A., Brummel O., Libuda J., Nielsen M. B., Kasper M.-P. (2021). Storing Energy with
Molecular Photoisomers. Joule.

[28] Meng F.-Y., Chen I.-H., Shen J.-Y., Chang K.-H., Chou T.-C., Chen Y.-A., Chen Y.-T., Chen C.-L., Chou P.-T. (2022). A New Approach
Exploiting Thermally Activated Delayed Fluorescence Molecules to Optimize
Solar Thermal Energy Storage. Nat. Commun..

[29] Hillers-Bendtsen A. E., Elholm J. L., Obel O. B., Hölzel H., Moth-Poulsen K., Mikkelsen K. V. (2023). Searching
the Chemical Space of Bicyclic
Dienes for Molecular Solar Thermal Energy Storage Candidates. Angew. Chem., Int. Ed..

[30] Borne K. D., Cooper J. C., Ashfold M. N., Bachmann J., Bhattacharyya S., Boll R., Bonanomi M., Bosch M., Callegari C., Centurion M. (2024). Ultrafast Electronic Relaxation Pathways of
the Molecular Photoswitch Quadricyclane. Nat.
Chem..

[31] Zähringer T. J., Lopez N. P., Schulte R., Schmitz M., Ihmels H., Kerzig C. (2025). Triplet-Sensitized
Switching of High-Energy-Density
Norbornadienes for Molecular Solar Thermal Energy Storage with Visible
Light. Angew. Chem., Int. Ed..

[32] Lee C., Yang W., Parr R. G. (1988). Development of the Colle-Salvetti
Correlation-Energy Formula into a Functional of the Electron Density. Phys. Rev. B.

[33] Becke A. D. (1993). Density-Functional Thermochemistry. III. The Role of
Exact Exchange. J. Chem. Phys..

[34] Lenthe E. v., Baerends E.-J., Snijders J. G. (1993). Relativistic
Regular Two-Component
Hamiltonians. J. Chem. Phys..

[35] Weigend F., Ahlrichs R. (2005). Balanced Basis Sets
of Split Valence, Triple Zeta Valence
and Quadruple Zeta Valence Quality for H to Rn: Design and Assessment
of Accuracy. Phys. Chem. Chem. Phys..

[36] Grimme S., Hansen A., Brandenburg J. G., Bannwarth C. (2016). Dispersion-Corrected
Mean-Field Electronic Structure Methods. Chem.
Rev..

[37] Yanai T., Tew D. P., Handy N. C. (2004). A New Hybrid Exchange-Correlation
Functional Using the Coulomb-Attenuating Method (CAM-B3LYP). Chem. Phys. Lett..

[38] Hirata S., Head-Gordon M. (1999). Time-Dependent Density Functional
Theory within the
Tamm-Dancoff Approximation. Chem. Phys. Lett..

[39] Acharya A., Chaudhuri S., Batista V. S. (2018). Can TDDFT describe excited electronic
states of naphthol photoacids? A closer look with EOM-CCSD. J. Chem. Theory Comput..

[40] Neese F. (2012). The ORCA Program
System. WIREs Comput. Mol. Sci..

[41] Neese F. (2022). Software Update:
The ORCA Program System–Version 5.0. WIREs Comput. Mol. Sci..

[42] Plasser F. (2020). TheoDORE:
A Toolbox for a Detailed and Automated Analysis of Electronic Excited
State Computations. J. Chem. Phys..

[43] Zobel J. P., Widmark P.-O., Veryazov V. (2020). The ANO-R Basis Set. J. Chem. Theory Comput..

[44] Pierloot K., Persson B. J., Roos B. O. (1995). Theoretical Study
of the Chemical
Bonding in Ni­(C_2_H_4_) and Ferrocene. J. Phys. Chem. A.

[45] Granovsky A. A. (2011). Extended
Multi-Configuration Quasi-Degenerate Perturbation Theory: The New
Approach to Multi-State Multi-Reference Perturbation Theory. J. Chem. Phys..

[46] Zobel J. P., Nogueira J. J., González L. (2017). The IPEA Dilemma
in CASPT2. Chem. Sci..

[47] Manni G. L., Galván I. F., Alavi A., Aleotti F., Aquilante F., Autschbach J., Avagliano D., Baiardi A., Bao J. J., Battaglia S. (2023). The OpenMolcas Web: A Community-Driven Approach
to Advancing Computational Chemistry. J. Chem.
Theory Comput..

[48] Salthouse R. J., Moth-Poulsen K. (2024). Multichromophoric
Photoswitches for Solar Energy Storage:
From Azobenzene to Norbornadiene, and MOST Things in Between. J. Mater. Chem. A.

[49] Wang Z., Hölzel H., Moth-Poulsen K. (2022). Status and Challenges for Molecular
Solar Thermal Energy Storage System Based Devices. Chem. Soc. Rev..

[50] Cotton F. A., Curtis N., Harris C., Johnson B., Lippard S., Mague J., Robinson W., Wood J. (1964). Mononuclear and Polynuclear
Chemistry of Rhenium (III): Its Pronounced Homophilicity. Science.

[51] Chan A. Y., Ghosh A., Yarranton J. T., Twilton J., Jin J., Arias-Rotondo D. M., Sakai H. A., McCusker J. K., MacMillan D. W. C. (2023). Exploiting
the Marcus Inverted Region for First-Row Transition Metal-Based Photoredox
Catalysis. Science.

[52] Ghosh A., Yarranton J. T., McCusker J. K. (2024). Establishing the Origin of Marcus-Inverted-Region
Behaviour in the Excited-State Dynamics of Cobalt (III) Polypyridyl
Complexes. Nat. Chem..

[53] Marcus R. A. (1956). On the
Theory of Oxidation-Reduction Reactions Involving Electron Transfer.
I. J. Chem. Phys..

[54] Marcus R. A. (1964). Chemical
and Electrochemical Electron-Transfer Theory. Annu. Rev. Phys. Chem..

[55] Dirac P. A. M. (1927). The
quantum theory of the emission and absorption of radiation. Proc. R. Soc. London, Ser. A.

[56] Atkins A. J., González L. (2017). Trajectory Surface-Hopping Dynamics Including Intersystem
Crossing in [Ru­(bpy)_3_]^2+^. J. Phys. Chem. Lett..

[57] Farkhutdinova D., Polonius S., Karrer P., Mai S., González L. (2025). Parametrization
of Linear Vibronic Coupling Models for Degenerate Electronic States. J. Phys. Chem. A.

[58] Dongare P., Myron B. D., Wang L., Thompson D. W., Meyer T. J. (2017). [Ru­(bpy)_3_]^2+*^ Revisited. Is It
Localized or Delocalized?
How Does It Decay?. Coord. Chem. Rev..

[59] Cannizzo A., van Mourik F., Gawelda W., Zgrablic G., Bressler C., Chergui M. (2006). Broadband
Femtosecond Fluorescence Spectroscopy of
[Ru­(bpy)_3_]^2+^. Angew. Chem.,
Int. Ed..

[60] Bhasikuttan A. C., Suzuki M., Nakashima S., Okada T. (2002). Ultrafast Fluorescence
Detection in Tris­(2,2’-bipyridine)­ruthenium­(II) Complex in
Solution: Relaxation Dynamics Involving Higher Excited States. J. Am. Chem. Soc..

[61] Bräm O., Messina F., El-Zohry A. M., Cannizzo A., Chergui M. (2012). Polychromatic
Femtosecond Fluorescence Studies of Metal–Polypyridine Complexes
in Solution. Chem. Phys..

[62] El-Sayed M. A. (1968). Triplet
State. Its Radiative and Nonradiative Properties. Acc. Chem. Res..

[63] Chaudhuri S., Hedström S., Méndez-Hernández D. D., Hendrickson H. P., Jung K. A., Ho J., Batista V. S. (2017). Electron
Transfer Assisted by Vibronic Coupling from Multiple Modes. J. Chem. Theory Comput..

[64] Ulstrup J., Jortner J. (1975). The effect of intramolecular quantum
modes on free
energy relationships for electron transfer reactions. J. Chem. Phys..

[65] Yang X., Keane T., Delor M., Meijer A. J., Weinstein J., Bittner E. R. (2017). Identifying electron transfer coordinates in donor-bridge-acceptor
systems using mode projection analysis. Nat.
Commun..

