# Supplementary material for: Unraveling the Photoisomerization Mechanism of Group‑8 Fulvalene-Bridged Bimetallic Complexes for Molecular Solar–Thermal Energy Storage
Source: J Am Chem Soc. 2025 Oct 30;147(45):41855–66. doi: 10.1021/jacs.5c14186 (PMC12616694; doi:10.1021/jacs.5c14186)
Supplement: Supplementary file 1 [file ja5c14186_si_001.pdf]

Supporting Information:

Unraveling the Photoisomerization Mechanism of Group-8 Fulvalene-Bridged Bimetallic Complexes for Molecular Solar–Thermal Energy Storage

Gaurab Ganguly * and Leticia González *

Institute of Theoretical Chemistry, Faculty of Chemistry, University of Vienna, Währinger Str. 17, 1090 Vienna, Austria

E-mail: gaurab.ganguly@univie.ac.at; leticia.gonzalez@univie.ac.at

Dedicated to the fond memory of Prof. Josef Michl (1939–2024), whose contributions continue to inspire us.

Table of Contents of the Supporting Information:

S1 DFT-optimized structures and Cartesian coordinates of the key intermediates in $(Fv)M_2(CO)_4$ (M = Ru, Fe) S-3

S2 Molecular orbital diagram for $(Fv)M_2(CO)_4$ (M = Ru, Fe) S-15

S3 Excited-state characterization of $(Fv)M_2(CO)_4$ (M = Ru, Fe) *via* fragmentation, natural transition orbital, and solvent-phase analysis S-17

S4 (XMS)-CASPT2(10e,14o) Spectral and State Character Analysis of $(Fv)M_2(CO)_4$ (M = Ru, Fe) S-21

S5 Ionization potential-electron affinity value calibration in XMS-CASPT2: Benchmarking against experimental data for $(Fv)M_2(CO)_4$ (M = Ru, Fe) S-24

S6 Active space natural orbital analysis in XMS-CASPT2(10e,12o): Decomposition into atomic orbital contributions S-25

S7 Spin-orbit coupling matrix elements for $(Fv)M_2(CO)_4$ (M = Ru, Fe) for the three lowest singlet and triplet states at the key geometries S-33

S8 Diabatic potential energy surfaces near the Frank-Condon (FC) region for $(Fv)M_2(CO)_4$ (M = Ru, Fe) S-39

S9 Intersystem crossing mechanism of the $(Fv)Os_2(CO)_4$ complex S-41

S10 Intersystem crossing mechanism of the $(Fv)FeRu(CO)_4$ complex S-42

S11 Assessment of semiclassical Marcus vs. Marcus–Levich–Jortner rates and vibronic-coupling in $(Fv)M_2(CO)_4$ complexes (M = Fe, Ru, Os) S-43

S12 Evolution of spin-orbit coupling between T_1 and S_0 states from Franck-Condon (FC) to *syn*-T_1 geometry in $(Fv)Ru_2(CO)_4$ S-51

S13 Final rearrangement in the Ru complex: High spin-orbit coupling drives triplet-to-singlet reverse intersystem crossing (rISC) and singlet photoproduct formation S-52

S14 Triplet-state relaxation in $(Fv)Fe_2(CO)_4$: *syn*-to-*anti* isomerization S-53

S1 DFT-optimized structures and Cartesian coordinates of the key intermediates in (Fv)$M_2(CO)_4$ (M = Ru, Fe)

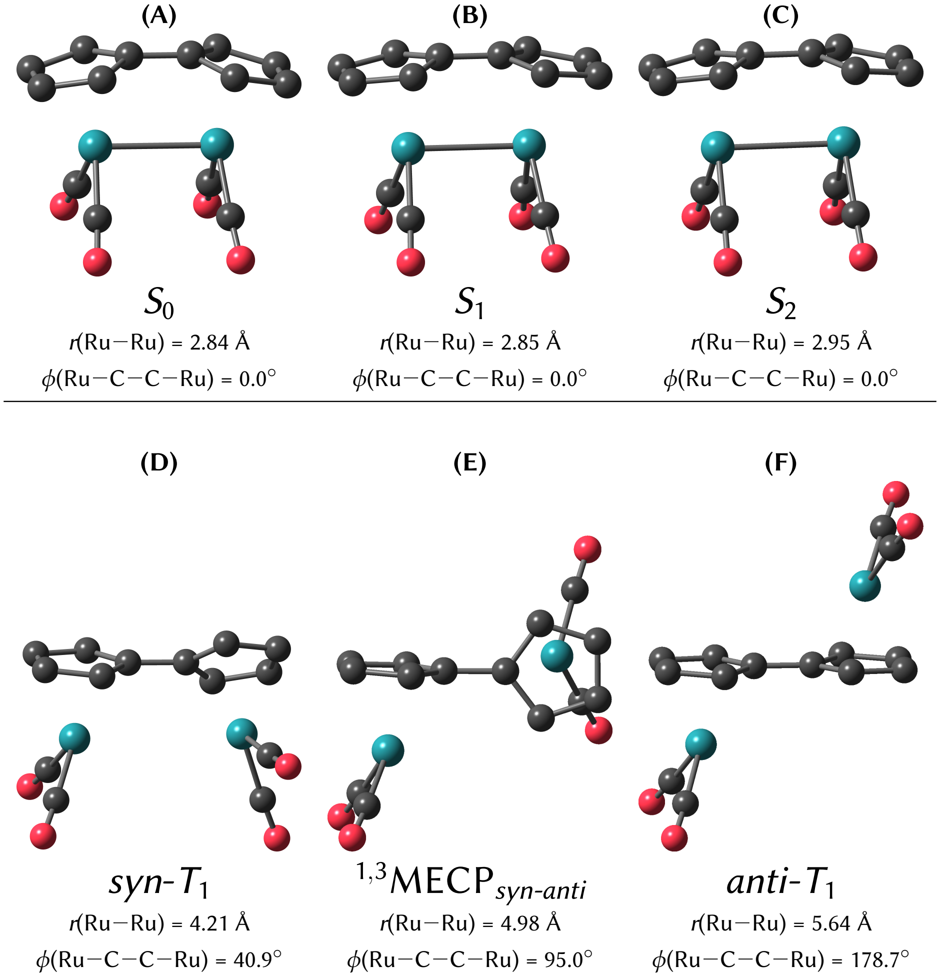

Figure S1: Geometries and key structural parameters for (Fv)$Ru_2(CO)_4$ in various electronic states. (**A**) DFT–optimized ground state (S_0) geometry. (**B**) TDDFT–optimized geometry in the first excited singlet state (S_1). (**C**) TDDFT–optimized geometry in the second excited singlet state (S_2). (**D**) DFT–optimized *syn* triplet state (*syn*-T_1) geometry. (**E**) DFT–optimized geometry of the minimum energy crossing point (^{1,3}MECP$_{syn-anti}$) between the singlet ground state (S_0) and the triplet state (T_1) along the *syn*–to–*anti* torsional angle coordinate. (**F**) DFT–optimized *anti* triplet state (*anti*-T_1) geometry. The key parameters include the Ru−Ru bond distance and the torsional angle between the ϕ(Ru−C−C−Ru) atoms, where the carbon atoms are part of the C−C bond connecting two Cp rings of the fulvalene ligand. These parameters are critical for understanding the photoisomerization process and structural changes upon excitation.

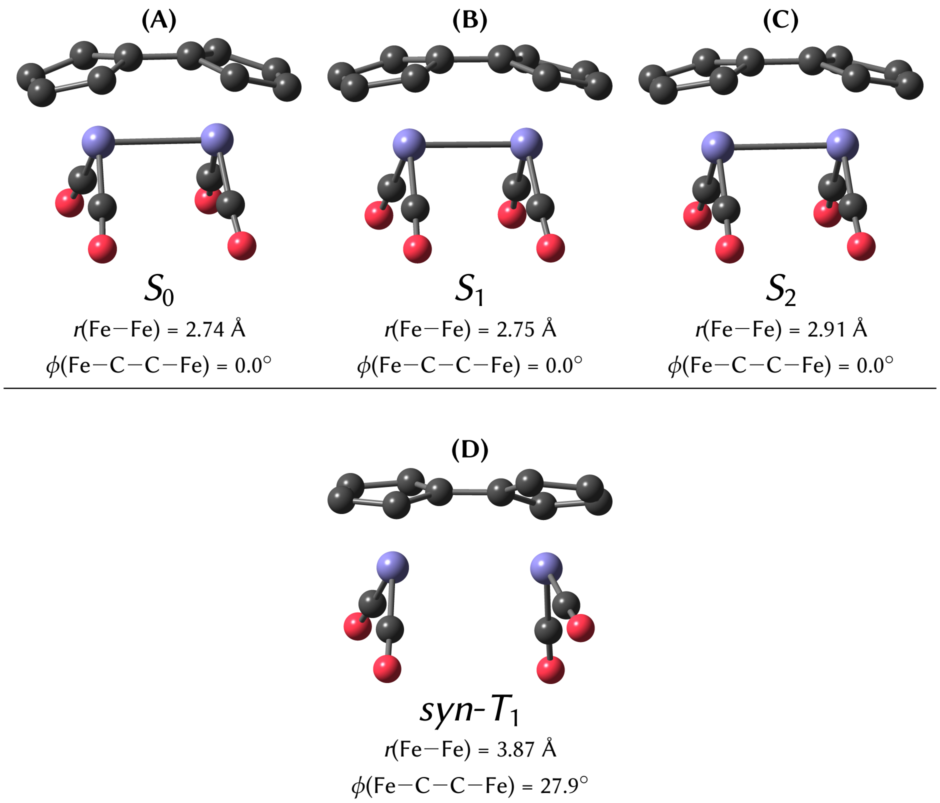

Figure S2: Geometries and key structural parameters for (Fv)$Fe_2(CO)_4$ in various electronic states. (**A**) DFT–optimized ground state (S_0) geometry. (**B**) TDDFT–optimized geometry in the first excited singlet state (S_1). (**C**) TDDFT–optimized geometry in the second excited singlet state (S_2). (**D**) DFT–optimized *syn* triplet state (*syn*-T_1) geometry. The key parameters include the distance of the Fe−Fe bond and the torsional angle between the ϕ(Fe−C−C−Fe) atoms, where the carbon atoms are part of the C−C bond that connects two Cp rings of the fulvalene ligand. These parameters are critical for understanding the photoisomerization process and structural changes upon excitation.

Table S1: Cartesian Coordinate for $(Fv)Ru_2(CO)_4$ at the FC Geometry

Atom	*x* (Å)	*y* (Å)	*z* (Å)
Ru	0.000000	1.419723	0.133707
Ru	-0.000000	-1.419723	0.133707
C	1.319055	1.550683	1.453176
C	-1.319055	-1.550683	1.453176
C	-1.319055	1.550683	1.453176
C	1.319055	-1.550683	1.453176
C	0.000000	0.722069	-2.037780
C	-0.000000	-0.722069	-2.037780
C	1.152038	1.548232	-1.835730
C	-1.152038	-1.548232	-1.835730
C	-1.152038	1.548232	-1.835730
C	1.152038	-1.548232	-1.835730
C	0.708735	2.861537	-1.525262
C	-0.708735	-2.861537	-1.525262
C	-0.708735	2.861537	-1.525262
C	0.708735	-2.861537	-1.525262
O	2.152198	1.671857	2.232870
O	-2.152198	-1.671857	2.232870
O	-2.152198	1.671857	2.232870
O	2.152198	-1.671857	2.232870
H	1.340562	3.712540	-1.336615
H	-1.340562	-3.712540	-1.336615
H	-1.340562	3.712540	-1.336615
H	1.340562	-3.712540	-1.336615
H	2.177218	1.229612	-1.907949
H	-2.177218	-1.229612	-1.907949
H	-2.177218	1.229612	-1.907949
H	2.177218	-1.229612	-1.907949

Table S2: Cartesian Coordinate for (Fv)$Ru_2(CO)_4$ at the *syn*-T_1 Geometry

Atom	x (Å)	y (Å)	z (Å)
Ru	0.030964	2.103380	0.200327
Ru	-0.030964	-2.103380	0.200327
C	1.000387	2.428613	1.782241
C	-1.000387	-2.428613	1.782241
C	-1.457580	3.050081	0.855173
C	1.457580	-3.050081	0.855173
C	0.248060	0.687033	-1.636777
C	-0.248060	-0.687033	-1.636777
C	1.541605	1.122309	-1.233234
C	-1.541605	-1.122309	-1.233234
C	-0.492832	1.842094	-2.015198
C	0.492832	-1.842094	-2.015198
C	1.615096	2.538930	-1.422355
C	-1.615096	-2.538930	-1.422355
C	0.366477	2.982959	-1.907404
C	-0.366477	-2.982959	-1.907404
O	1.632883	2.661885	2.710032
O	-1.632883	-2.661885	2.710032
O	-2.359969	3.667717	1.202619
O	2.359969	-3.667717	1.202619
H	2.473824	3.156825	-1.222077
H	-2.473824	-3.156825	-1.222077
H	0.102669	3.997891	-2.149795
H	-0.102669	-3.997891	-2.149795
H	2.333911	0.482815	-0.882643
H	-2.333911	-0.482815	-0.882643
H	-1.500027	1.840850	-2.395765
H	1.500027	-1.840850	-2.395765

Table S3: Cartesian Coordinate for (Fv)$Ru_2(CO)_4$ at the ^{1,3}MECP$_{syn\text{-}anti}$ Geometry.

Atom	*x* (Å)	*y* (Å)	*z* (Å)
Ru	0.01808404	-2.48784149	0.15297595
Ru	-0.01068381	2.49107604	0.15327187
C	0.44050990	-0.60472379	-1.14682990
C	-0.40383098	0.59802870	-1.14656902
C	0.26128421	-1.73265341	-1.99609209
C	-0.23386681	1.72721690	-1.99673857
C	1.56863443	-0.86763961	-0.32042271
C	-1.53729679	0.84947614	-0.32455840
C	1.31459715	-2.66639730	-1.73336274
C	-1.29922801	2.64818943	-1.73838379
C	2.11805969	-2.13368563	-0.70138197
C	-2.10104915	2.10811278	-0.70889894
C	-0.40622788	2.95412060	1.93673582
C	0.41164550	-2.99800680	1.92461075
C	1.14558642	3.96546966	-0.03468558
C	-1.20882634	-3.90191006	-0.05069605
O	0.71835939	-3.31820846	2.98224991
O	-1.91638220	-4.78799995	-0.22483694
O	1.80972322	4.88598057	-0.20130138
O	-0.71499309	3.25281426	3.00009663
H	-0.50489176	-1.82986466	-2.74651882
H	0.53224216	1.83192000	-2.74617725
H	1.96570320	-0.19005858	0.41660167
H	-1.92857014	0.16753834	0.41161817
H	1.46511107	-3.60678481	-2.23537249
H	-1.45835604	3.58658835	-2.24156935
H	2.99078610	-2.59697196	-0.27374358
H	-2.98124143	2.56103621	-0.28576917

Table S4: Cartesian Coordinate for (Fv)$Ru_2(CO)_4$ at the *anti*-T_1 Geometry.

Atom	x (Å)	y (Å)	z (Å)
Ru	2.822257	-0.018843	0.006933
Ru	-2.822257	0.018843	0.006933
C	4.064074	-0.470556	1.346724
C	-4.064074	0.470556	1.346724
C	4.038408	-0.540093	-1.331765
C	-4.038408	0.540093	-1.331765
C	0.526613	0.501561	-0.015129
C	-0.526613	-0.501561	-0.015129
C	1.143409	1.080670	1.131494
C	-1.143409	-1.080670	1.131494
C	1.160918	1.060836	-1.161353
C	-1.160918	-1.060836	-1.161353
C	2.093647	2.053596	0.688897
C	-2.093647	-2.053596	0.688897
C	2.104488	2.042050	-0.722391
C	-2.104488	-2.042050	-0.722391
O	4.822247	-0.684250	2.180882
O	-4.822247	0.684250	2.180882
O	4.784463	-0.801142	-2.163227
O	-4.784463	0.801142	-2.163227
H	2.692770	2.683576	1.323736
H	-2.692770	-2.683576	1.323736
H	2.713200	2.661090	-1.358715
H	-2.713200	-2.661090	-1.358715
H	0.880605	0.881275	2.156025
H	-0.880605	-0.881275	2.156025
H	0.918736	0.838082	-2.186207
H	-0.918736	-0.838082	-2.186207

Table S5: Cartesian Coordinate for $(Fv)Fe_2(CO)_4$ at the FC Geometry.

Atom	*x* (Å)	*y* (Å)	*z* (Å)
Fe	0.000000	1.371541	0.111007
Fe	0.000000	-1.371541	0.111007
C	1.280093	1.482806	1.311495
C	-1.280093	-1.482806	1.311495
C	-1.280093	1.482806	1.311495
C	1.280093	-1.482806	1.311495
C	0.000000	0.722585	-1.873524
C	-0.000000	-0.722585	-1.873524
C	1.150061	1.537572	-1.643483
C	-1.150061	-1.537572	-1.643483
C	-1.150061	1.537572	-1.643483
C	1.150061	-1.537572	-1.643483
C	0.705799	2.835065	-1.270480
C	-0.705799	-2.835065	-1.270480
C	-0.705799	2.835065	-1.270480
C	0.705799	-2.835065	-1.270480
O	2.134401	1.613060	2.064548
O	-2.134401	-1.613060	2.064548
O	-2.134401	1.613060	2.064548
O	2.134401	-1.613060	2.064548
H	1.338335	3.668512	-1.016499
H	-1.338335	-3.668512	-1.016499
H	-1.338335	3.668512	-1.016499
H	1.338335	-3.668512	-1.016499
H	2.175174	1.217721	-1.707595
H	-2.175174	-1.217721	-1.707595
H	-2.175174	1.217721	-1.707595
H	2.175174	-1.217721	-1.707595

Table S6: Cartesian Coordinate for (Fv)$Fe_2(CO)_4$ at the *syn*-T_1 Geometry.

Atom	*x* (Å)	*y* (Å)	*z* (Å)
Fe	0.10933686	1.93373125	0.17142685
Fe	-0.10933686	-1.93373125	0.17142685
C	1.05403626	1.55595316	1.62217557
C	-1.05403626	-1.55595316	1.62217557
C	-1.02803822	2.98144556	1.02911333
C	1.02803822	-2.98144556	1.02911333
C	0.20647284	0.70075838	-1.60368639
C	-0.20647284	-0.70075838	-1.60368639
C	1.50563389	1.19559766	-1.29109525
C	-1.50563389	-1.19559766	-1.29109525
C	-0.62652535	1.82701470	-1.82925297
C	0.62652535	-1.82701470	-1.82925297
C	1.49743553	2.61809408	-1.40339349
C	-1.49743553	-2.61809408	-1.40339349
C	0.18074845	3.00778677	-1.70489599
C	-0.18074845	-3.00778677	-1.70489599
O	1.69058661	1.32257148	2.54585978
O	-1.69058661	-1.32257148	2.54585978
O	-1.75020308	3.69635886	1.55894164
O	1.75020308	-3.69635886	1.55894164
H	2.33686018	3.27080949	-1.23669737
H	-2.33686018	-3.27080949	-1.23669737
H	-0.17117890	4.01776650	-1.83150773
H	0.17117890	-4.01776650	-1.83150773
H	2.35793046	0.58691340	-1.03931292
H	-2.35793046	-0.58691340	-1.03931292
H	-1.66775679	1.80147010	-2.10174470
H	1.66775679	-1.80147010	-2.10174470

Table S7: Cartesian Coordinate for (Fv)$Os_2(CO)_4$ at the FC Geometry.

Atom	x (Å)	y (Å)	z (Å)
Os	0.00000000	1.42646471	0.10414496
Os	0.00000000	-1.42646471	0.10414496
C	-1.31358083	1.57762276	1.42468418
C	-1.31358083	-1.57762276	1.42468418
C	1.31358083	1.57762276	1.42468418
C	1.31358083	-1.57762276	1.42468418
C	0.00000000	0.72629444	-2.08178920
C	0.00000000	-0.72629444	-2.08178920
C	-1.15489019	1.54812604	-1.88187988
C	-1.15489019	-1.54812604	-1.88187988
C	1.15489019	1.54812604	-1.88187988
C	1.15489019	-1.54812604	-1.88187988
C	-0.71104515	2.85902430	-1.54593777
C	-0.71104515	-2.85902430	-1.54593777
C	0.71104515	2.85902430	-1.54593777
C	0.71104515	-2.85902430	-1.54593777
O	-2.15248907	1.71060079	2.20232943
O	-2.15248907	-1.71060079	2.20232943
O	2.15248907	1.71060079	2.20232943
O	2.15248907	-1.71060079	2.20232943
H	-1.34265876	3.70884428	-1.35343527
H	-1.34265876	-3.70884428	-1.35343527
H	1.34265876	3.70884428	-1.35343527
H	1.34265876	-3.70884428	-1.35343527
H	-2.17910496	1.22883757	-1.95854006
H	-2.17910496	-1.22883757	-1.95854006
H	2.17910496	1.22883757	-1.95854006
H	2.17910496	-1.22883757	-1.95854006

Table S8: Cartesian Coordinate for $(Fv)Os_2(CO)_4$ at the *syn*-T_1 Geometry.

Atom	x (Å)	y (Å)	z (Å)
Os	-0.04114056	2.15609316	0.13066502
Os	0.04114056	-2.15609316	0.13066502
C	-0.89122319	2.05094525	1.80534143
C	-1.15080467	-3.44925335	0.78423262
C	1.15080467	3.44925335	0.78423262
C	0.89122319	-2.05094525	1.80534143
C	-0.26402432	0.68214622	-1.64613681
C	0.26402432	-0.68214622	-1.64613681
C	-1.56254919	1.08541950	-1.22989418
C	-0.43202935	-1.85158409	-2.09106100
C	0.43202935	1.85158409	-2.09106100
C	1.56254919	-1.08541950	-1.22989418
C	-1.68560133	2.49747581	-1.43204106
C	0.45642410	-2.96394125	-1.96921194
C	-0.45642410	2.96394125	-1.96921194
C	1.68560133	-2.49747581	-1.43204106
O	-1.45537774	1.98386235	2.80583601
O	-1.87020429	-4.27668051	1.13727765
O	1.87020429	4.27668051	1.13727765
O	1.45537774	-1.98386235	2.80583601
H	-2.55824025	3.09380260	-1.23178480
H	0.23129567	-3.98236691	-2.23551188
H	-0.23129567	3.98236691	-2.23551188
H	2.55824025	-3.09380260	-1.23178480
H	-2.31681355	0.42828292	-0.83146174
H	-1.42559220	-1.86910248	-2.50406617
H	1.42559220	1.86910248	-2.50406617
H	2.31681355	-0.42828292	-0.83146174

Table S9: Cartesian Coordinate for (Fv)RuFe$(CO)_4$ at the FC Geometry.

Atom	x (Å)	y (Å)	z (Å)
Ru	-0.10041896	1.26925904	0.00000000
Fe	-0.14789015	-1.51101008	0.00000000
C	-1.40732176	1.48101473	1.32161013
C	-1.36094000	-1.54047507	-1.27342804
C	-1.40732176	1.48101473	-1.32161013
C	-1.36094000	-1.54047507	1.27342804
C	2.01172093	0.44505414	0.00000000
C	1.88750863	-0.99485595	0.00000000
C	1.87189264	1.28341837	1.15205793
C	1.59651130	-1.79040003	-1.15022631
C	1.87189264	1.28341837	-1.15205793
C	1.59651130	-1.79040003	1.15022631
C	1.65558944	2.61608715	0.70806828
C	1.12990783	-3.05721511	-0.70618880
C	1.65558944	2.61608715	-0.70806828
C	1.12990783	-3.05721511	0.70618880
O	-2.17392615	1.65640782	2.15800829
O	-2.12956998	-1.61640901	-2.11944448
O	-2.17392615	1.65640782	-2.15800829
O	-2.12956998	-1.61640901	2.11944448
H	1.52678094	3.47827465	1.33976134
H	0.81643205	-3.86998334	-1.33882564
H	1.52678094	3.47827465	-1.33976134
H	0.81643205	-3.86998334	1.33882564
H	1.92262649	0.96088518	2.17712978
H	1.68259575	-1.47477685	-2.17510496
H	1.92262649	0.96088518	-2.17712978
H	1.68259575	-1.47477685	2.17510496

Table S10: Cartesian Coordinate for (Fv)RuFe(CO)$_4$ at the *syn*-T_1 Geometry.

Atom	*x* (Å)	*y* (Å)	*z* (Å)
Ru	-0.12119192	1.79784706	0.00000000
Fe	-0.30993195	-2.21059508	0.00000000
C	-1.26784350	2.44917913	1.34234444
C	-1.49328159	-2.40379999	-1.30165912
C	-1.26784350	2.44917913	-1.34234444
C	-1.49328159	-2.40379999	1.30165912
C	1.71489865	0.32112605	0.00000000
C	1.59913731	-1.13221111	0.00000000
C	1.77294553	1.16542839	1.14593090
C	1.50135897	-1.97212497	-1.14266513
C	1.77294553	1.16542839	-1.14593090
C	1.50135897	-1.97212497	1.14266513
C	1.94647186	2.51559892	0.70554366
C	1.42721785	-3.33291948	-0.70251773
C	1.94647186	2.51559892	-0.70554366
C	1.42721785	-3.33291948	0.70251773
O	-1.92481206	2.89198777	2.17199696
O	-2.23556619	-2.55209692	-2.16194042
O	-1.92481206	2.89198777	-2.17199696
O	-2.23556619	-2.55209692	2.16194042
H	2.05800208	3.37655548	1.34170284
H	1.36358412	-4.19537109	-1.34372189
H	2.05800208	3.37655548	-1.34170284
H	1.36358412	-4.19537109	1.34372189
H	1.76118609	0.83742538	2.17108654
H	1.53226875	-1.64755254	-2.16864371
H	1.76118609	0.83742538	-2.17108654
H	1.53226875	-1.64755254	2.16864371

S2 Molecular orbital diagram for $(Fv)M_2(CO)_4$ (M = Ru, Fe)

The electronic excitations in bimetallic systems can be described using a molecular orbital (MO) diagram, where the *d* atomic orbitals (AOs) of the metal centers are considered. Each metal contributes five *d* AOs, resulting in a total of ten *d* AOs for the two metals. These ten AOs combine to form the 10 MOs illustrated in Supporting Figure S3.

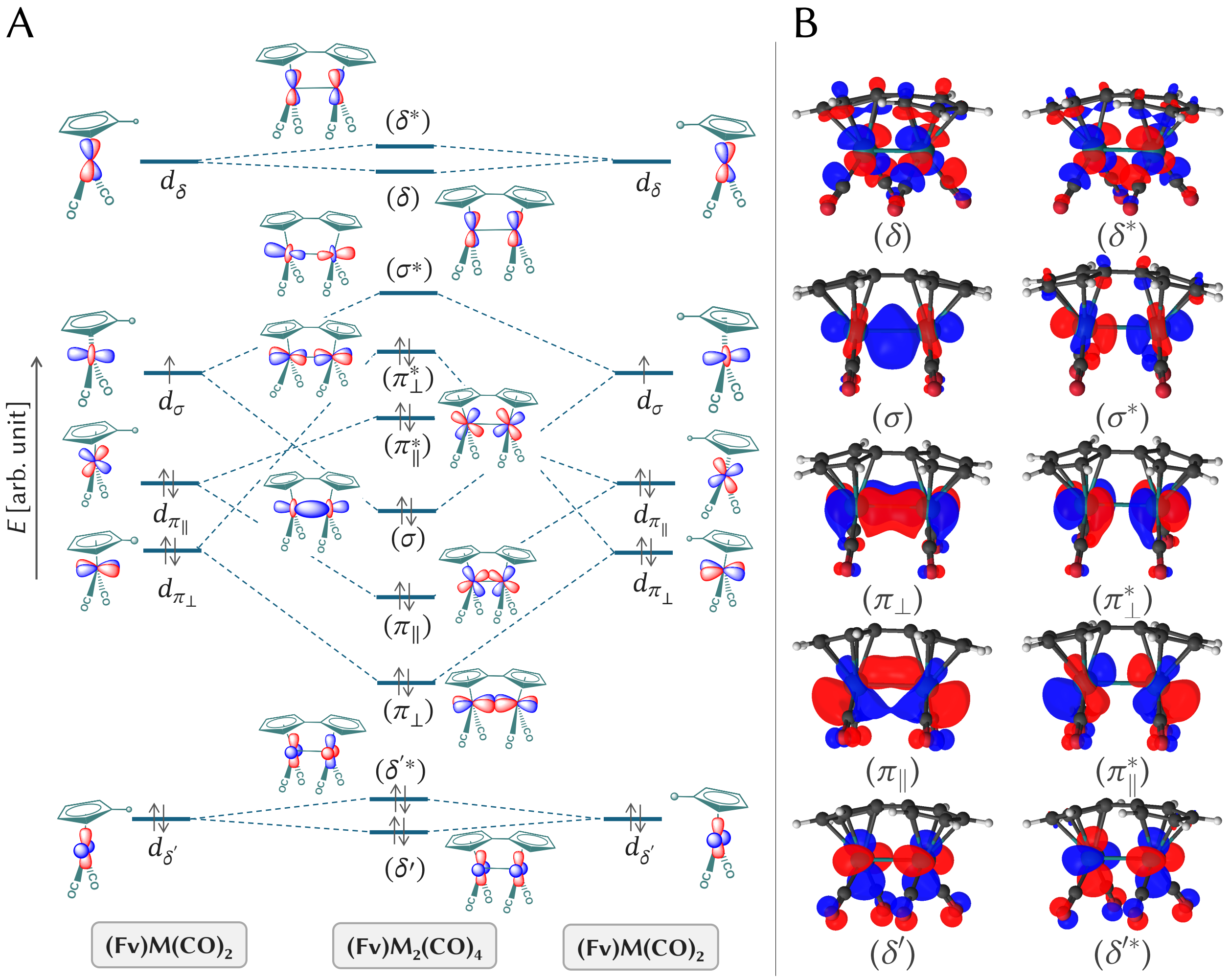

Figure S3: Molecular orbital (MO) diagram of the $(Fv)M_2(CO)_4$ (M=Ru,Fe) complex. (**A**) Qualitative representation of *d* atomic orbitals (AOs) interactions in $(Cp)M(CO)_2$ (Cp = cyclopentadienyl anion) fragments and formation of MOs in the $(Fv)M_2(CO)_4$ complex depicted as cartoon orbitals (M=Ru,Fe). (**B**) Corresponding MO isosurfaces (± 0.4) of the ground state of $(Fv)Ru_2(CO)_4$.

These orbitals can be classified as:

- **Bonding MOs (+ in-phase)**: Five bonding MOs arise from the in-phase combination of *d* AOs.

- **Anti-bonding MOs (− out-of-phase)**: Five anti-bonding MOs result from the out-of-phase combination of d AOs.

In an ideal $D_{\infty h}$ symmetry, the MOs are further classified as follows:

- σ **MOs**: The σ bonding and σ^* anti-bonding orbitals arise from head-on interactions between the metal d orbitals along the metal-metal axis.
- π **MOs**: The $\pi_{\|}$ and $\pi_{\perp}$ bonding and anti-bonding orbitals correspond to in-plane and out-of-plane side-on interactions between the metal d orbitals.
- δ **MOs**: The δ and δ' bonding and anti-bonding orbitals result from interactions between the metal d orbitals, involving four lobes.

In the ground state, the $\pi_{\|}$ (bonding and anti-bonding), $\pi_{\perp}$ (bonding and anti-bonding), and δ' (bonding and anti-bonding) MOs are doubly occupied, while the δ (bonding) and δ^* (anti-bonding) MOs remain unoccupied (Supporting Figure S3). The metal-metal σ-bond in the ground state is primarily formed by the doubly occupied σ (bonding) MO and the unoccupied σ^* (anti-bonding) MO.

S3 Excited-state characterization of $(Fv)M_2(CO)_4$ (M = Ru, Fe) *via* fragmentation, natural transition orbital, and solvent-phase analysis

Characterizing electronically excited states in transition metal complexes by identifying the largest coefficients of the time-dependent density functional theory (TDDFT) excitation vectors and visualizing the corresponding canonical Kohn-Sham MOs can be tedious. Particularly when distinguishing between localized and charge-transfer (CT) excitations can be subjective and imprecise. Alternatively, a molecular fragment-based analysis of excited states using charge transfer numbers as implemented in the TheoDORE program[S1,S2] is convenient. Charge transfer numbers are leveraged to classify excited states based only on the well-defined one-electron transition density matrix (1TDM) partitioned between different groups of atoms. In this study, the bimetallic complex is considered the sum of three fragments, as indicated in Supporting Figure S4A. By determining the charge transfer between electron and hole position, it is possible to classify the states into metal-centered (MC), metal-to-ligand CT (MLCT) to the CO or the fulvalene, ligand-to-metal CT (LMCT), ligand-to-ligand CT (LLCT), and intraligand (IL) states. Additionally, TheoDORE provides a range of plotting functionalities that facilitate effective visualization and analysis. Here, we employ stacked bar plots that display the decomposition of excited states into localized and charge-transfer components according to the chosen fragmentation scheme, together with excitation energies and oscillator strengths of each state (Figure S4A and Figure S5A).

TheoDORE can also generate natural transition orbitals (NTOs) for singlet and triplet excited states *via* singular value decomposition of the 1TDM[S1,S2], offering a concise, chemist-friendly description of the excited-state character (Supporting Figure S4B and Figure S5B).

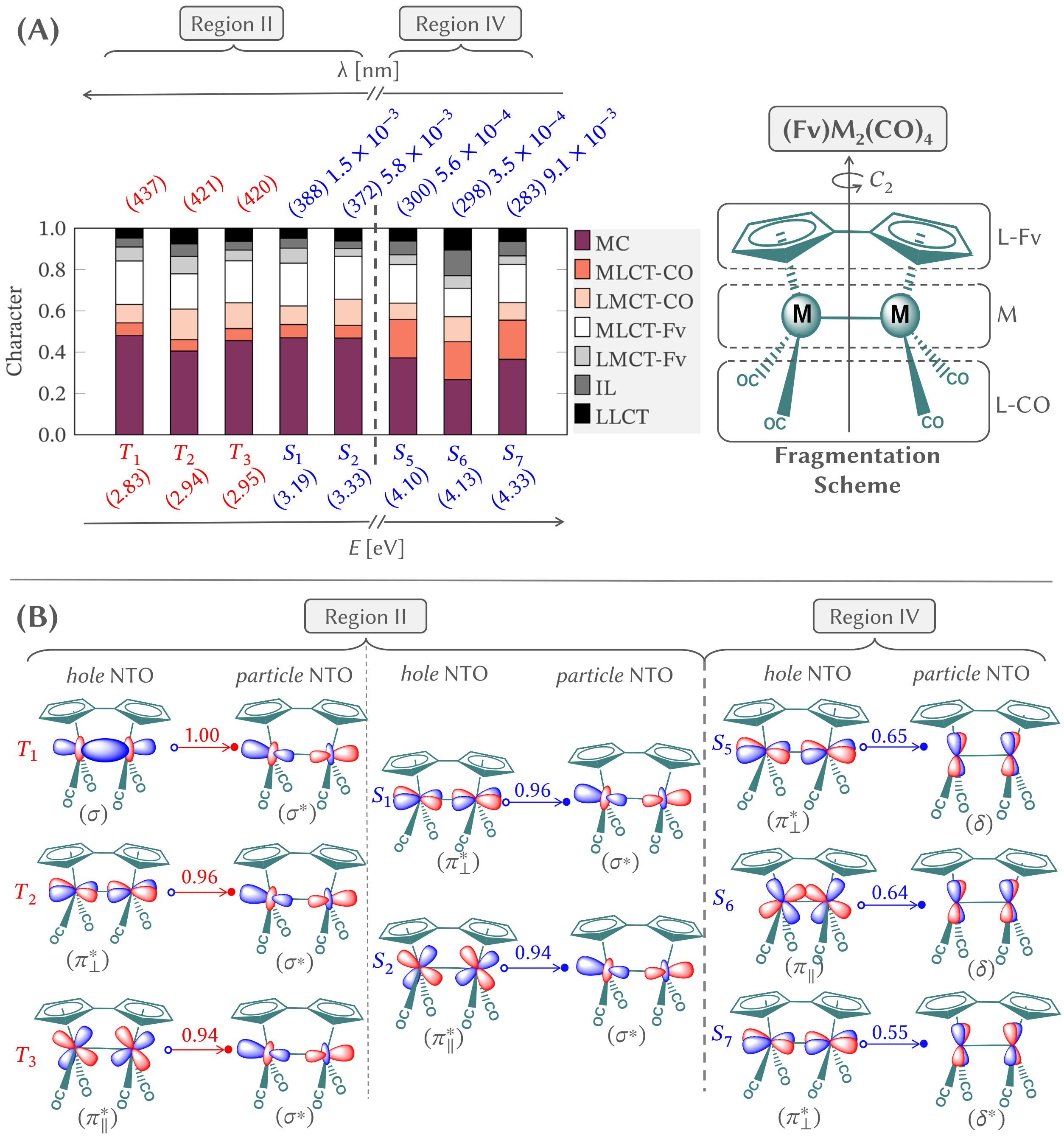

Figure S4: Analysis of TDDFT excited-states for (Fv)$Ru_2(CO)_4$. The first three triplet and two singlet states composing Region II, and first three bright singlet states of Region IV are shown. **(A)** The stacked bar plot illustrates the decomposition of excited–states into localized and charge–transfer character, with excitation energies and oscillator strengths provided for each bar. The molecule fragmentation scheme is displayed on the right side. **(B)** Leading NTO pairs (weights labeled above arrows) for states in Region II and Region IV depicted with cartoon orbitals for clarity.

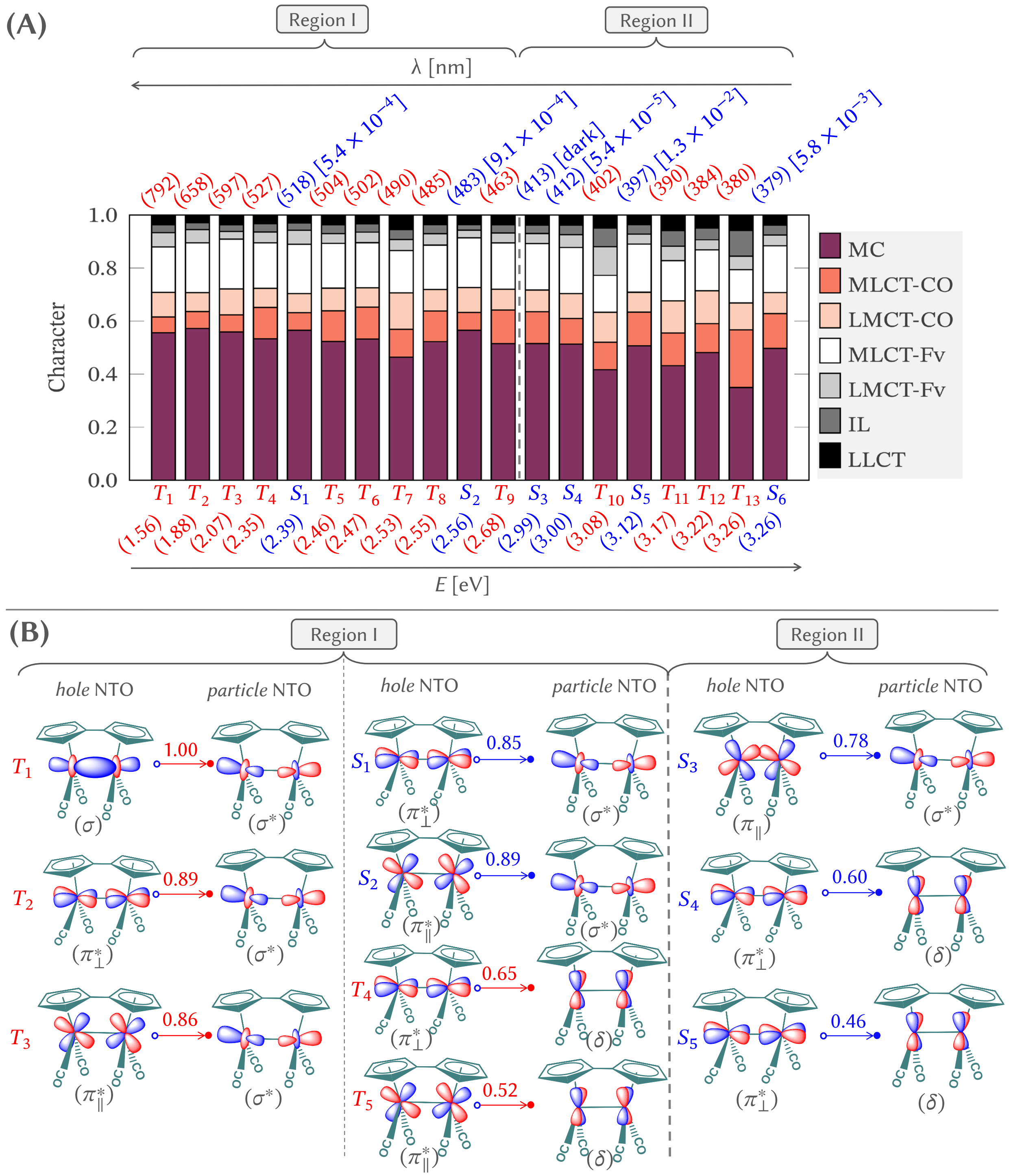

Figure S5: Analysis of TDDFT excited–states for (Fv)Fe$_2$(CO)$_4$. The first nine triplet and two singlet states of Region I, and first four singlet and four triplet states in Region II are shown. **(A)** Stacked bar plot illustrates the decomposition of excited–states into localized and charge–transfer character, with excitation energies and oscillator strengths provided for each bar. **(B)** Leading NTO pairs (weights labeled above arrows) for the first five triplet states and two singlet states in Region I and the first three bright singlet states in Region II depicted with cartoon orbitals for clarity.

Table S11: Excitation energies (in eV) for selected triplet and singlet states of (Fv)$Fe_2(CO)_4$ and (Fv)$Ru_2(CO)_4$ at the FC geometry, calculated using TDA-TDDFT in gas phase and in solvents of increasing dielectric constant.

Complex	State	Gas ($\varepsilon = 0.0$)	Heptane ($\varepsilon = 1.9$)	Acetonitrile ($\varepsilon = 35.9$)	Water ($\varepsilon = 78.4$)
(Fv)$Fe_2(CO)_4$	T_1	1.57	1.59	1.62	1.62
	T_2	1.88	1.90	1.92	1.92
	T_3	2.07	2.10	2.13	2.13
	T_4	2.35	2.36	2.38	2.38
	T_5	2.46	2.47	2.50	2.50
	S_1	2.39	2.40	2.42	2.42
	S_2	2.56	2.58	2.60	2.60
(Fv)$Ru_2(CO)_4$	T_1	2.83	2.86	2.89	2.89
	T_2	2.94	2.98	3.04	3.04
	T_3	2.95	2.99	3.04	3.05
	S_1	3.19	3.21	3.24	3.24
	S_2	3.33	3.36	3.41	3.42

The excitation energies of Table S11 show minimal variation across solvents with different dielectric constants. In particular, the changes between gas phase and heptane (the experimental solvent) are less than 0.1 eV for all states, indicating that solvent effects on the excitation energies are negligible in this system. This justifies the validity of comparing our gas-phase calculations directly with experimental data obtained in heptane.[S3,S4]

S4 (XMS)-CASPT2(10e,14o) Spectral and State Character Analysis of $(Fv)M_2(CO)_4$ (M = Ru, Fe)

To investigate the decarboxylation mechanism, we additionally performed (XMS)-CASPT2 calculations with a $10e/14o$ active space, including the δ and δ^* orbitals. The (XMS)-CASPT2(10e,14o) spectra for $(Fv)M_2(CO)_4$ (M = Ru, Fe) are presented in Figure S6 and analyzed in Table S12 and Table S13.

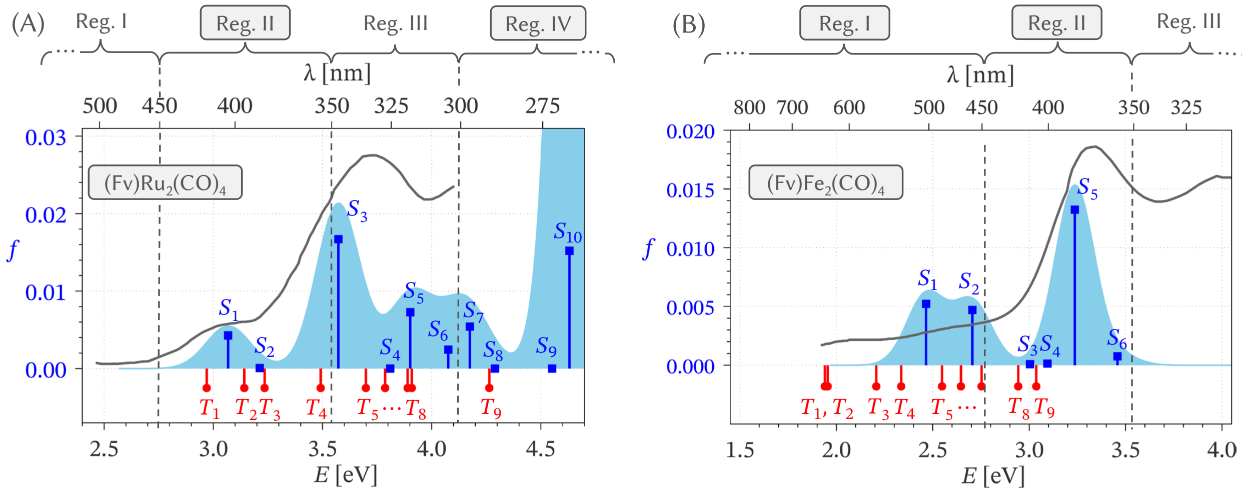

Figure S6: (SA)-(XMS)-CASPT2(10e,14o) *vs.* experimental UV-vis absorption spectra for $(Fv)Ru_2(CO)_4$ (left (A), SA(12S,9T)) and $(Fv)Fe_2(CO)_4$ (right (B), SA(12S,9T)). Vertical blue sticks denote singlet excited states, while vertical (inverted) red sticks indicate triplet states. Skyblue filled curves depict Gaussian-broadened calculated spectra ($\sigma = 0.15$ eV for individual transitions), and gray solid lines depict experimental spectra digitized from Ref. S5 (Ru) and Ref. S4 (Fe). Vertical gray dashed lines separate Regions (I-IV). Regions II and IV in $(Fv)Ru_2(CO)_4$ (left) and Regions I and II in $(Fv)Fe_2(CO)_4$ (right) are highlighted in gray shaded boxes, signifying the primary focus throughout the text.

Table S12: Excitation energies, oscillator strengths, and electronic wavefunctions for the singlet and triplet excited states spanning Regions II to IV, described in terms of fractionally occupied natural orbitals (NOs), for (Fv)$Ru_2(CO)_4$ based on (SA)-XMS-CASPT2(10e,14o) calculations, with the spectrum presented in Figure S6A.

	# St	E [eV]	λ [nm]	f [$\times 10^{-3}$]	**Wavefunction** [NO Occupation]
Reg. II	T_1	2.97	417	–	$\sim {}^3\left[(\pi^*_\perp)^{1.0}(\sigma^*)^{1.0}\right]$
	T_2	3.14	395	–	$\sim {}^3\left[(\pi^*_\parallel)^{1.0}(\sigma^*)^{1.0}\right]$
	T_3	3.23	383	–	$\sim {}^3\left[(\sigma)^{1.0}(\sigma^*)^{1.0}\right]$
	S_1	3.07	404	4.28	$\sim {}^1\left[(\pi^*_\perp)^{1.0}(\sigma^*)^{1.0}\right]$
	S_2	3.21	386	0.06	$\sim {}^1\left[(\pi^*_\parallel)^{1.0}(\sigma^*)^{1.0}\right]$
Reg. III	S_3	3.57	347	16.74	$\sim {}^1\left[(\pi^*_\perp)^{1.5}(\pi^*_\parallel)^{1.5}(\sigma^*)^{0.6}(\delta)^{0.4}\right]$
	S_4	3.81	347	–	$\sim {}^1\left[(\pi_\parallel)^{1.8}(\pi^*_\parallel)^{1.3}(\sigma^*)^{0.3}(\delta)^{0.7}\right]$
	S_5	3.90	318	7.28	$\sim {}^1\left[(\pi^*_\perp)^{1.5}(\pi^*_\parallel)^{1.5}(\sigma^*)^{0.7}(\delta)^{0.4}\right]$
	S_6	4.08	304	2.44	$\sim {}^1\left[(\pi_\parallel)^{1.3}(\pi^*_\parallel)^{1.6}(\delta)^{0.6}(\delta^*)^{0.4}\right]$
Reg. IV	S_7	4.18	297	5.42	$\sim {}^1\left[(\pi_\perp)^{1.7}(\pi^*_\perp)^{1.2}(\delta)^{0.2}(\delta^*)^{0.8}\right]$
	S_{10}	4.63	268	15.22	$\sim {}^1\left[(\sigma)^{1.3}(\pi^*_\parallel)^{1.7}(\delta)^{0.6}(\delta^*)^{0.4}\right]$

Table S13: Excitation energies, oscillator strengths, and electronic wavefunctions for the singlet and triplet excited states spanning Regions I to II, described in terms of fractionally occupied natural orbitals (NOs), for (Fv)$Fe_2(CO)_4$ based on (SA)-XMS-CASPT2(10e,14o) calculations, with the spectrum presented in Figure S6B.

	**# St**	E	λ	f	**Wavefunction**
		[eV]	[nm]	[$\times 10^{-3}$]	[NO Occupation]
Reg. I	T_1	1.94	639	–	$\sim {}^3\left[(\sigma)^{1.0}(\sigma^*)^{1.0}\right]$
	T_2	1.96	633	–	$\sim {}^3\left[(\pi^*_\perp)^{1.0}(\sigma^*)^{1.0}\right]$
	T_3	2.21	561	–	$\sim {}^3\left[(\sigma)^{1.6}(\pi_\perp)^{1.6}(\pi^*_\perp)^{1.3}(\sigma^*)^{0.3}(\delta)^{0.6}(\delta^*)^{0.4}\right]$
	T_4	2.34	530	–	$\sim {}^3\left[(\sigma)^{1.6}(\pi_\perp)^{1.6}(\pi^*_\perp)^{1.4}(\sigma^*)^{0.4}(\delta)^{0.4}(\delta^*)^{0.5}\right]$
	T_5	2.55	486	–	$\sim {}^3\left[(\pi^*_\parallel)^{1.0}(\sigma^*)^{1.0}\right]$
	S_1	2.47	503	5.23	$\sim {}^1\left[(\sigma)^{1.8}(\pi_\perp)^{1.8}(\pi^*_\perp)^{1.2}(\sigma^*)^{1.2}\right]$
	S_2	2.71	458	4.70	$\sim {}^1\left[(\sigma)^{1.8}(\pi_\parallel)^{1.8}(\pi^*_\parallel)^{1.2}(\sigma^*)^{1.2}\right]$
Reg. II	S_5	4.18	297	13.24	$\sim {}^1\left[(\sigma)^{1.6}(\pi_\perp)^{1.8}(\pi^*_\perp)^{1.2}(\sigma^*)^{0.4}(\delta)^{0.8}(\delta^*)^{0.2}\right]$

S5 Ionization potential-electron affinity value calibration in XMS-CASPT2: Benchmarking against experimental data for $(Fv)M_2(CO)_4$ (M = Ru, Fe)

The Ionization Potential-Electron Affinity (IPEA) shift is a parameter in CASPT2 that adjusts the zeroth-order Hamiltonian to correct systematic errors in open-shell states[S6]. Our benchmarking against experimental data for $(Fv)M_2(CO)_4$ (M = Ru, Fe) confirms the properly calibrated IPEA values for Ru and Fe, as presented in Supporting Table S14.

Table S14: Excitation Energies (in nm) for $(Fv)M_2(CO)_4$ (M = Ru, Fe) Obtained with the IPEA-Corrected Zeroth-Order Hamiltonian for Various IPEA Shift (ε) Values Using (XMS)-CASPT2/ANO-R[a], Compared to Experimental Peak Positions[b], with a Focus on the Most Intense Peak (λ_{max}) and the Onset (λ_{onset})

		$(Fv)Ru_2(CO)_4$			$(Fv)Fe_2(CO)_4$		
Expt.		~ 410 nm (λ_{onset})	–	~ 330 nm (λ_{max})	~ 500 nm (λ_{onset})	–	~ 370 nm (λ_{max})
CASPT2	ε	S_1	S_2	S_3	S_1	S_2	S_3
	0.00	**395**	**382**	**328**	531	488	420
	0.15	365	355	305	**503**	**458**	**379**
	0.25	351	341	293	488	444	360
	0.35	339	330	283	474	432	343
	0.45	329	320	274	462	422	329

[a]ANO-R2 basis set for Ru/Fe, ANO-R1 basis set for C and O, and ANO-R0 basis set for H.
[b]Experimental values are taken from ref. S5 and ref. S4 (Fe)

In passing, we note that since there are no experimental spectra to compare with for the Osd and RuFe complexes, the IPEA value in the corresponding calculations is the default value of 0.25 a.u.

S6 Active space natural orbital analysis in XMS-CASPT2(10e,12o): Decomposition into atomic orbital contributions

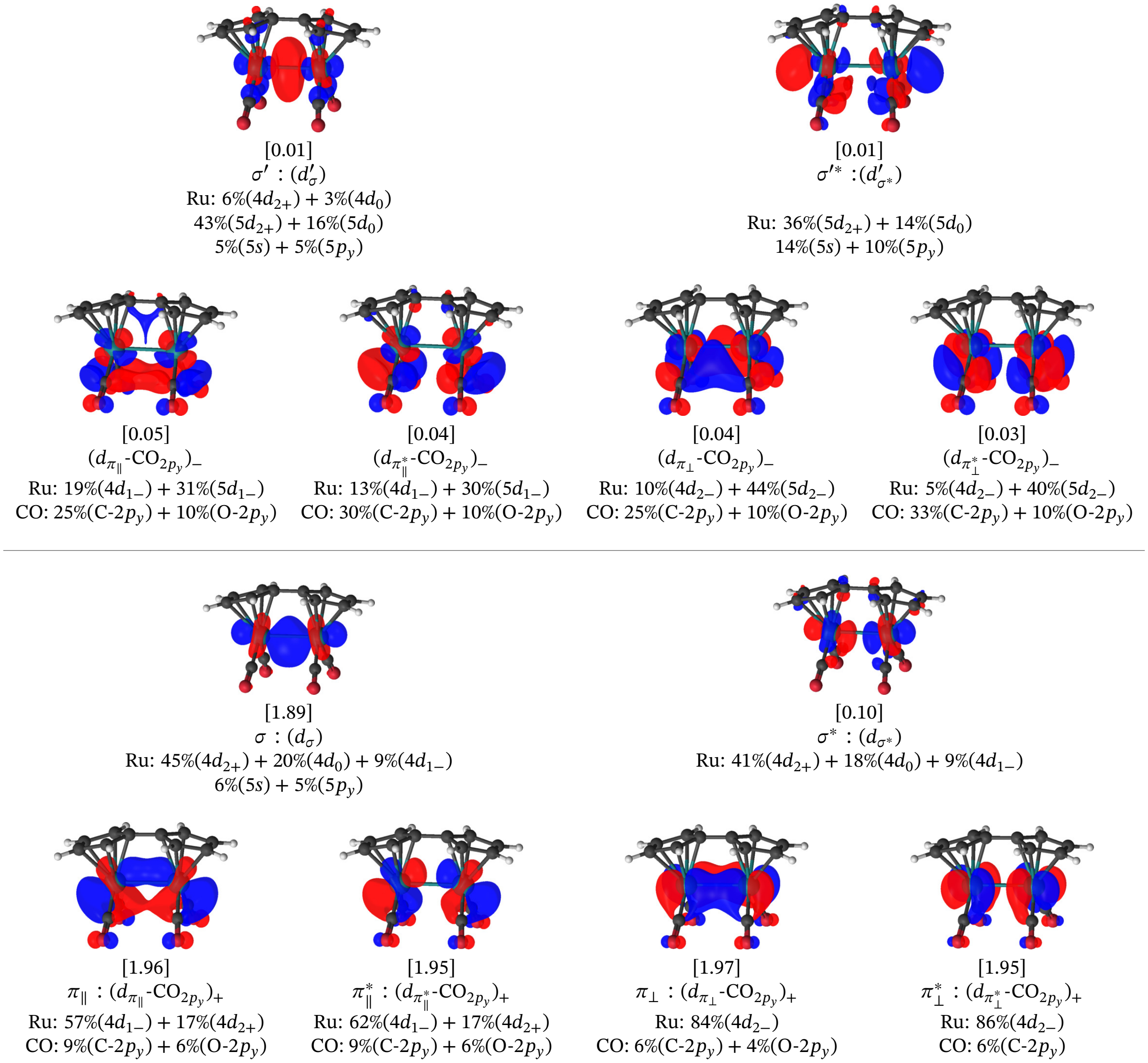

Figure S7: Isosurfaces ($\pm$ 0.04 au) of state-averaged state-specific natural orbitals (SA-SS-NOs) of the S_0 state at the DFT-optimized S_0 geometry of (Fv)$Ru_2(CO)_4$, derived from a SA(5S)-(XMS)-CAS(10e,12o)PT2 calculation. The NO occupations are given in square brackets below each isosurface plot. These NOs were analyzed for their decomposition in terms of atomic orbital (AO) wt% to facilitate bonding analysis. Lower panel: NOs describe one σ and two π bondings; Middle panel: The NOs with involvement of CO π orbitals and double d-shell; and Top panel: The δ/δ^* orbitals.

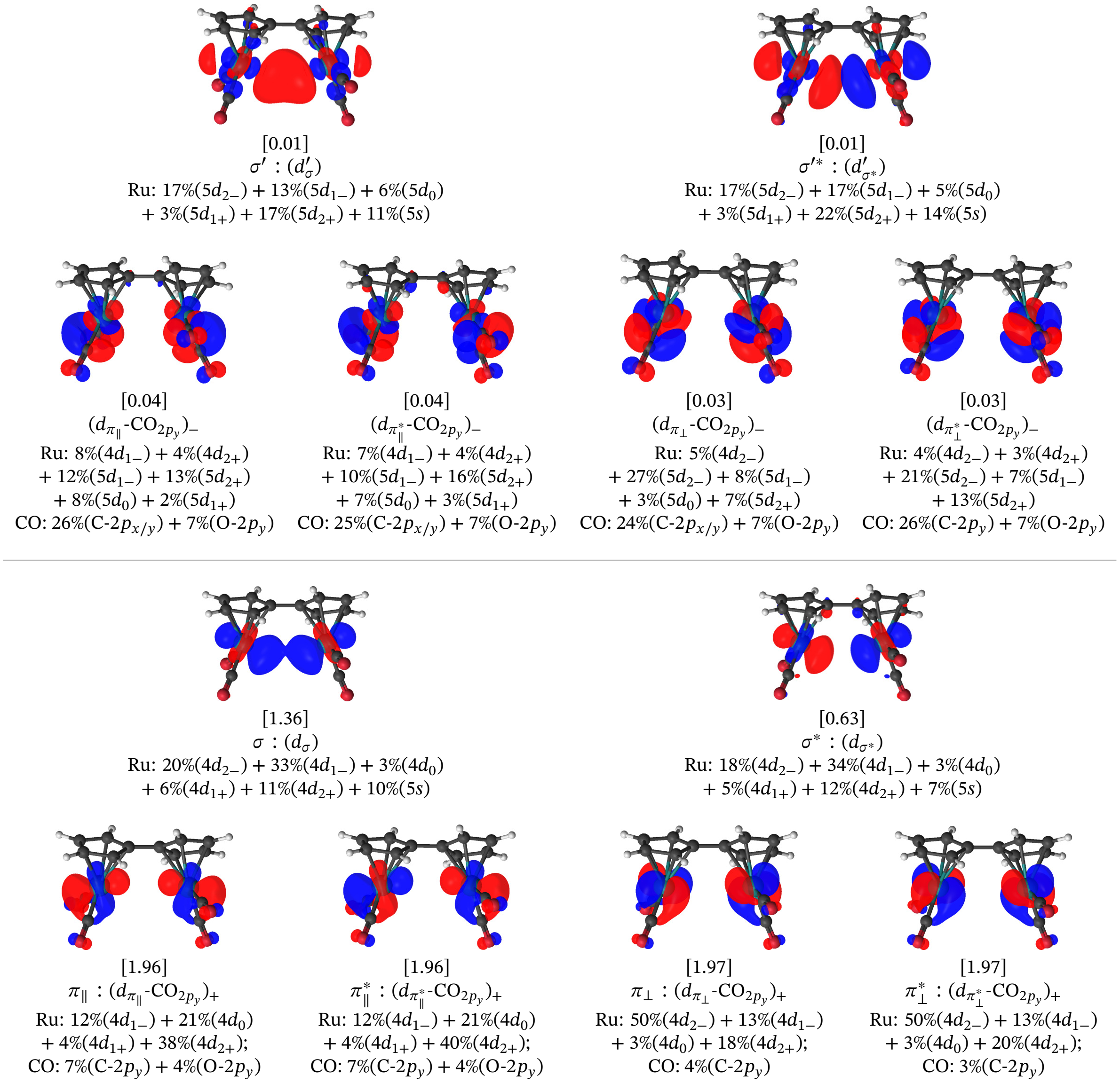

Figure S8: Isosurfaces ($\pm$ 0.04 au) of state-averaged state-specific natural orbitals (SA-SS-NOs) of the S_0 state at the DFT-optimized *syn*-T_1 geometry of (Fv)$Ru_2(CO)_4$, derived from a SA(5S)-(XMS)-CAS(10e,12o)PT2 calculation. The NO occupations are given in square brackets below each isosurface plot. These NOs were analyzed for their decomposition in terms of atomic orbital (AO) wt% to facilitate bonding analysis. Lower panel: NOs describe one σ and two π bondings; and Top panel: The NOs with involvement of CO π orbitals and double d-shell.

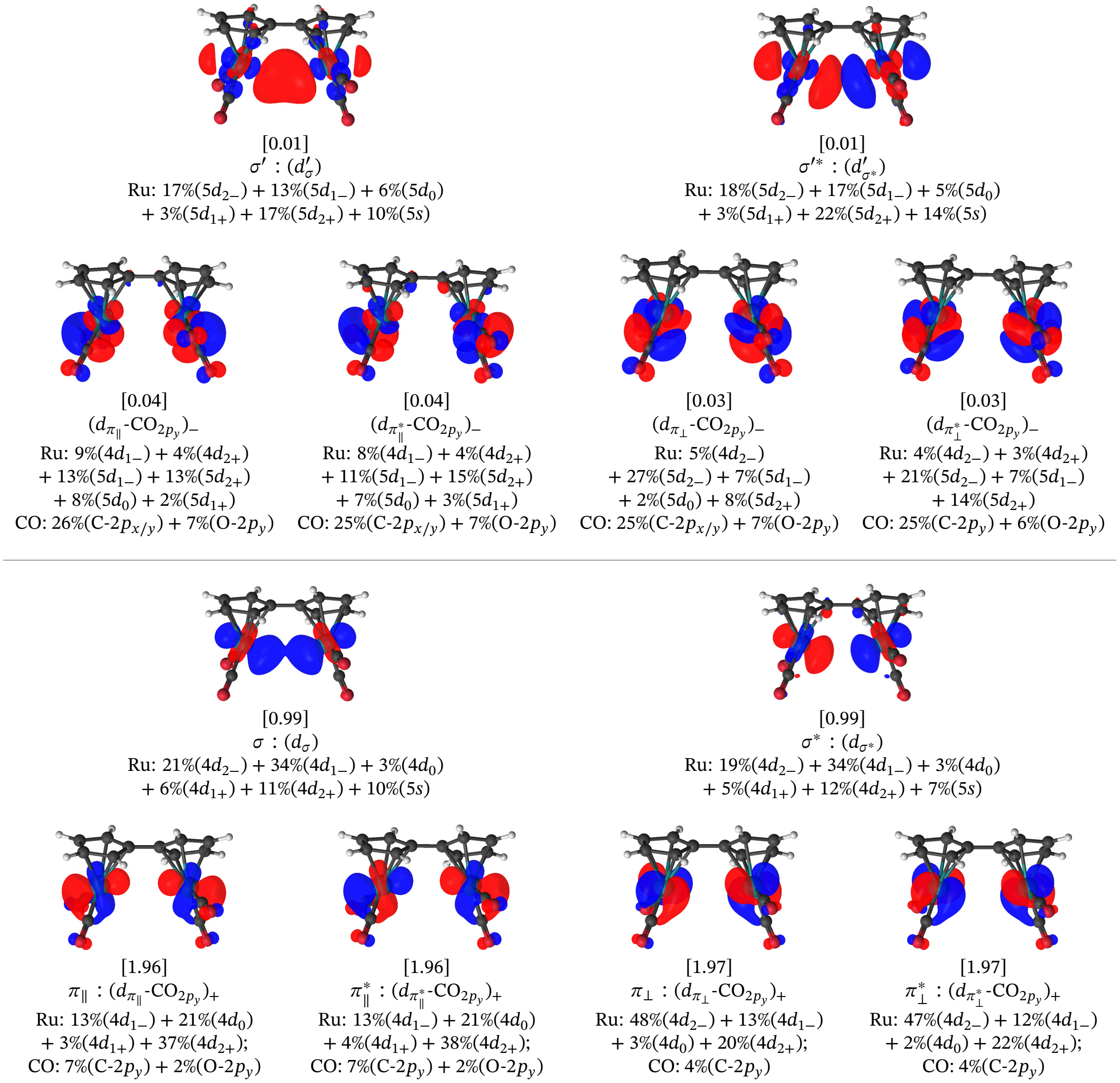

Figure S9: Isosurfaces ($\pm$ 0.04 au) of state-averaged state-specific natural orbitals (SA-SS-NOs) of the T_1 state at the DFT-optimized *syn*-T_1 geometry of (Fv)$Ru_2(CO)_4$, derived from a SA(5T)-(XMS)-CAS(10e,12o)PT2 calculation. The NO occupations are given in square brackets below each isosurface plot. These NOs were analyzed for their decomposition in terms of atomic orbital (AO) wt% to facilitate bonding analysis. Lower panel: NOs describe one σ and two π bondings; and Top panel: The NOs with involvement of CO π orbitals and double d-shell.

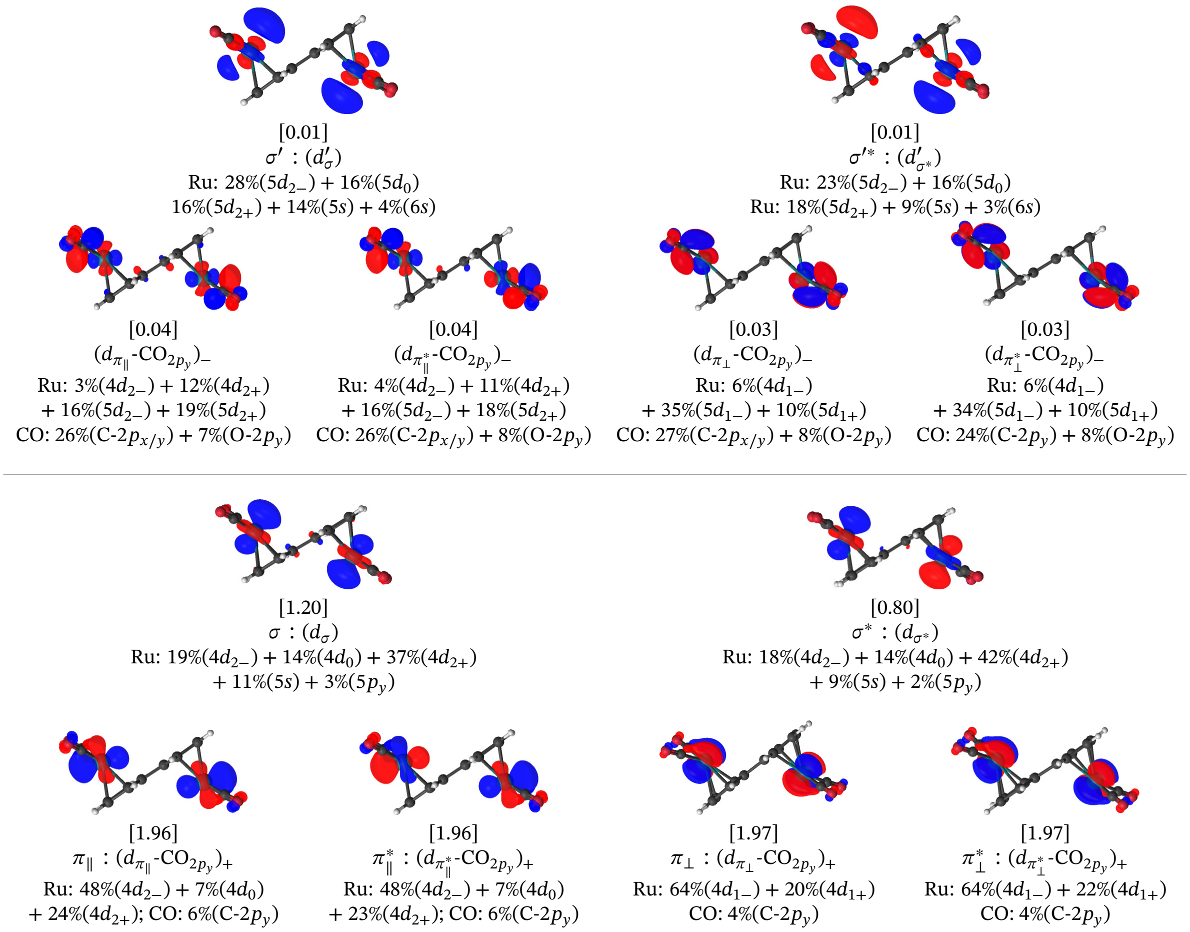

Figure S10: Isosurfaces (± 0.04 au) of state-averaged state-specific natural orbitals (SA-SS-NOs) of the S_0 state at the DFT-optimized *anti*-T_1 geometry of (Fv)$Ru_2(CO)_4$, derived from a SA(5*S*)-(XMS)-CAS(10e,12o)PT2 calculation. The NO occupations are given in square brackets below each isosurface plot. These NOs were analyzed for their decomposition in terms of atomic orbital (AO) wt% to facilitate bonding analysis. Lower panel: NOs describe one σ and two π bondings; and Top panel: The NOs with involvement of CO π orbitals and double d-shell.

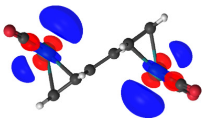

[0.01]

σ' : (d'_{σ})

Ru: 28%($5d_{2-}$) + 16%($5d_0$)

16%($5d_{2+}$) + 14%($5s$)

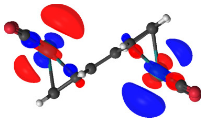

[0.01]

σ'^{*} : (d'_{σ^*})

Ru: 23%($5d_{2-}$) + 16%($5d_0$)

Ru: 18%($5d_{2+}$) + 10%($5s$)

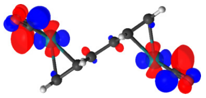

[0.04]

$(d_{\pi_{\parallel}}\text{-CO}_{2p_y})_{-}$

Ru: 3%($4d_{2-}$) + 12%($4d_{2+}$)

+ 15%($5d_{2-}$) + 20%($5d_{2+}$)

CO: 20%(C-$2p_y$) + 8%(O-$2p_y$)

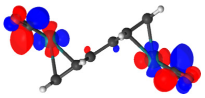

[0.04]

$(d_{\pi^*_{\parallel}}\text{-CO}_{2p_y})_{-}$

Ru: 4%($4d_{2-}$) + 11%($4d_{2+}$)

+ 16%($4d_{1-}$) + 18%($5d_{2+}$)

CO: 20%(C-$2p_y$) + 8%(O-$2p_y$)

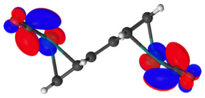

[0.03]

$(d_{\pi_{\perp}}\text{-CO}_{2p_y})_{-}$

Ru: 6%($4d_{1-}$)

+ 35%($5d_{1-}$) + 10%($5d_{1+}$)

CO: 22%(C-$2p_y$) + 8%(O-$2p_y$)

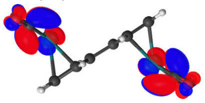

[0.03]

$(d_{\pi^*_{\perp}}\text{-CO}_{2p_y})_{-}$

Ru: 6%($4d_{1-}$)

+ 35%($5d_{1-}$) + 10%($5d_{1+}$)

CO: 22%(C-$2p_y$) + 8%(O-$2p_y$)

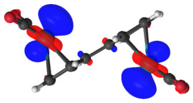

[0.99]

σ : (d_{σ})

Ru: 36%($4d_{2+}$) + 15%($4d_0$) + 20%($4d_{2-}$)

+ 11%($5s$) + 3%($5p_y$)

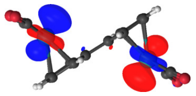

[0.99]

σ^* : (d_{σ^*})

Ru: 43%($4d_{2+}$) + 14%($4d_0$) + 18%($4d_{2-}$)

+ 9%($5s$) + 2%($5p_y$)

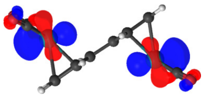

[1.96]

$\pi_{\parallel}$: $(d_{\pi_{\parallel}}\text{-CO}_{2p_y})_{+}$

Ru: 48%($4d_{2-}$) + 22%($4d_{2+}$)

+ 7%($4d_0$); CO: 6%(C-$2p_y$)

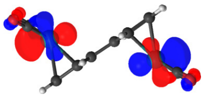

[1.96]

$\pi^*_{\parallel}$: $(d_{\pi^*_{\parallel}}\text{-CO}_{2p_y})_{+}$

Ru: 48%($4d_{2-}$) + 22%($4d_{2+}$)

+ 7%($4d_0$); CO: 6%(C-$2p_y$)

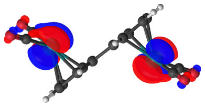

[1.97]

$\pi_{\perp}$: $(d_{\pi_{\perp}}\text{-CO}_{2p_y})_{+}$

Ru: 64%($4d_{1-}$) + 20%($4d_{1+}$)

CO: 4%(C-$2p_y$)

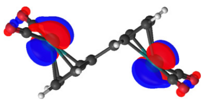

[1.97]

$\pi^*_{\perp}$: $(d_{\pi^*_{\perp}}\text{-CO}_{2p_y})_{+}$

Ru: 64%($4d_{1-}$) + 20%($4d_{1+}$)

CO: 4%(C-$2p_y$)

Figure S11: Isosurfaces (± 0.04 au) of state-averaged state-specific natural orbitals (SA-SS-NOs) of the T_1 state at the DFT-optimized *anti*-T_1 geometry of (Fv)$Ru_2(CO)_4$, derived from a SA(5T)-(XMS)-CAS(10e,12o)PT2 calculation. The NO occupations are given in square brackets below each isosurface plot. These NOs were analyzed for their decomposition in terms of atomic orbital (AO) wt% to facilitate bonding analysis. Lower panel: NOs describe one σ and two π bondings; and Top panel: The NOs with involvement of CO π orbitals and double d-shell.

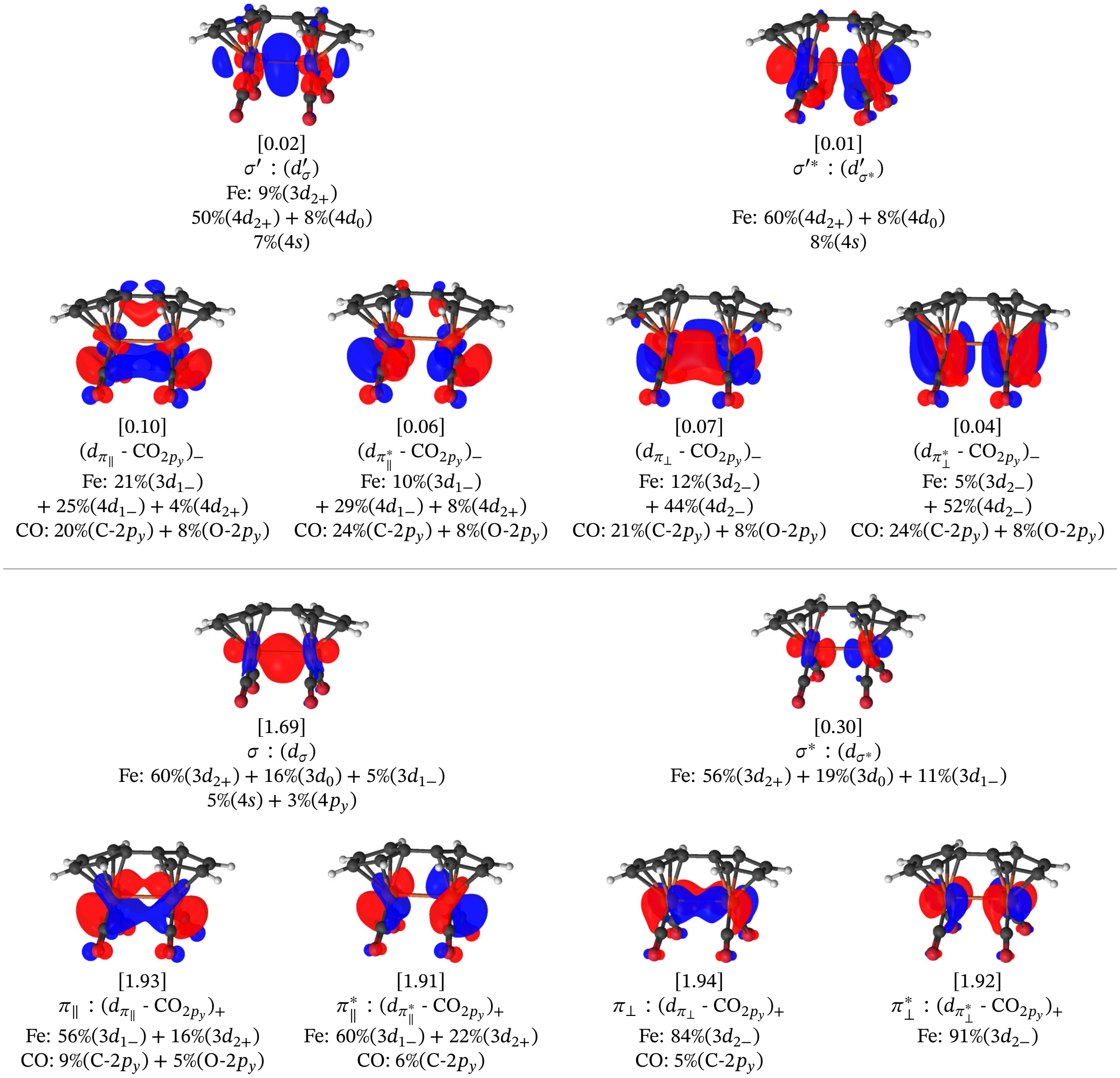

Figure S12: Isosurfaces (± 0.04 au) of state-averaged state-specific natural orbitals (SA-SS-NOs) of the S_0 state at the DFT-optimized S_0 geometry of (Fv)$Fe_2(CO)_4$, derived from a SA(5S)-(XMS)-CAS(10e,12o)PT2 calculation. The NO occupations are given in square brackets below each isosurface plot. These NOs were analyzed for their decomposition in terms of atomic orbital (AO) wt% to facilitate bonding analysis. Lower panel: NOs describe one σ and two π bondings; Middle panel: The NOs with involvement of CO π orbitals and double d-shell; and Top panel: The δ/δ^* orbitals.

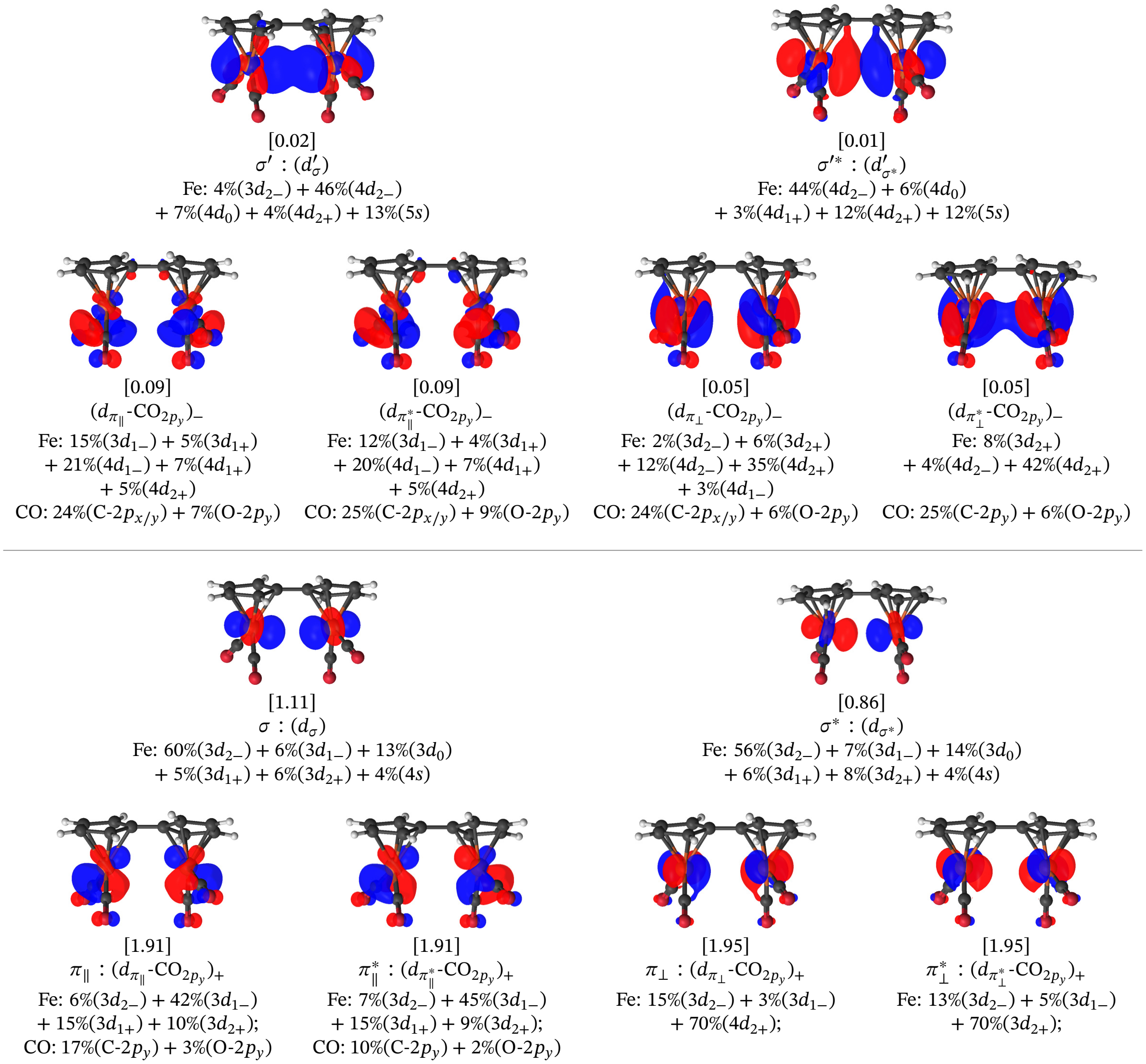

Figure S13: Isosurfaces (± 0.04 au) of state-averaged state-specific natural orbitals (SA-SS-NOs) of the S_0 state at the DFT-optimized *syn* T_1 geometry of (Fv)$Fe_2(CO)_4$, derived from a SA(5*S*)-(XMS)-CAS(10e,12o)PT2 calculation. The NO occupations are given in square brackets below each isosurface plot. These NOs were analyzed for their decomposition in terms of atomic orbital (AO) wt% to facilitate bonding analysis. Lower panel: NOs describe one σ and two π bondings; and Top panel: The NOs with involvement of CO π orbitals and double *d*-shell.

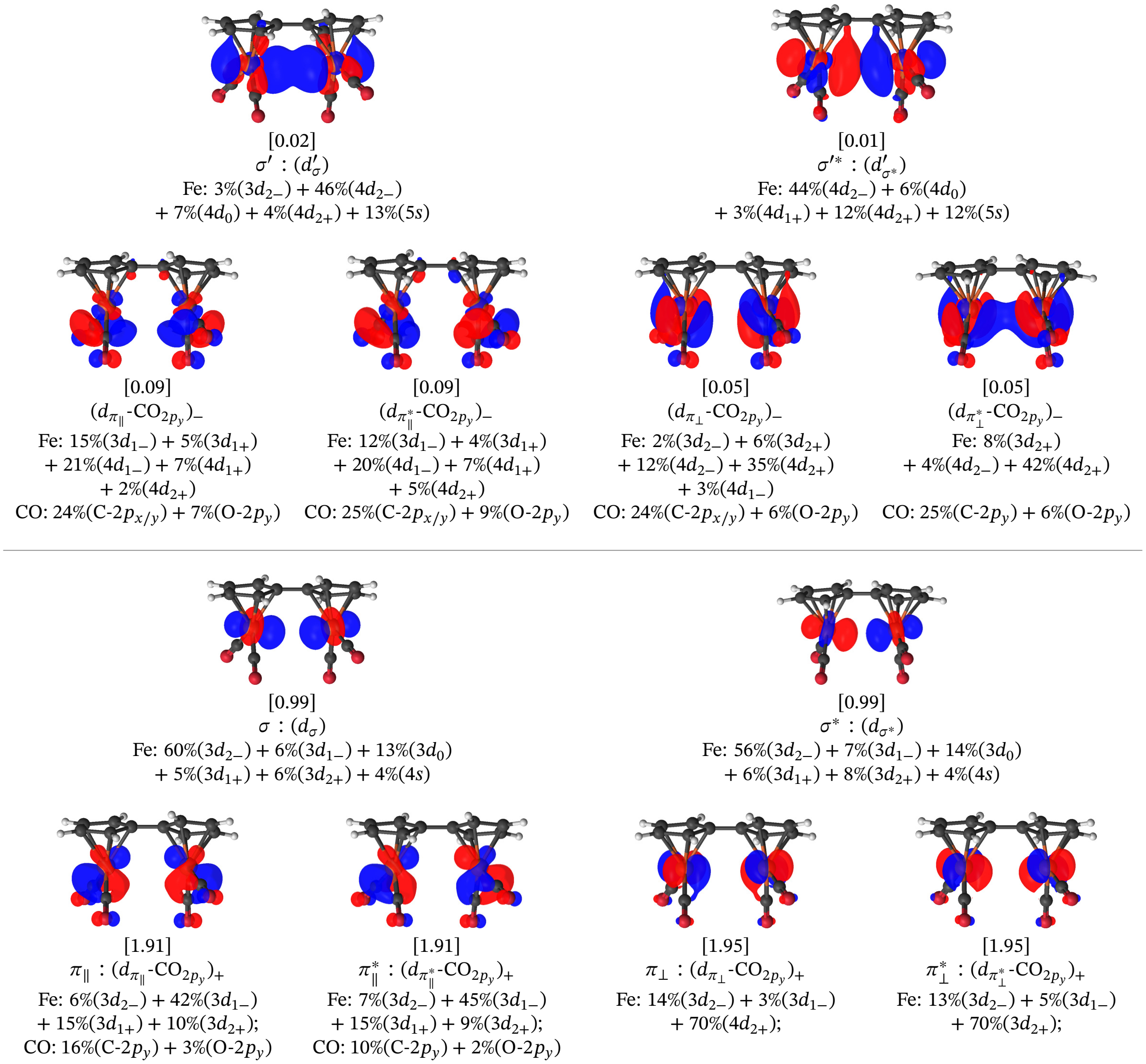

Figure S14: Isosurfaces (± 0.04 au) of state-averaged state-specific natural orbitals (SA-SS-NOs) of the T_1 state at the DFT-optimized *syn* T_1 geometry of (Fv)$Fe_2(CO)_4$, derived from a SA(5T)-(XMS)-CAS(10e,12o)PT2 calculation. The NO occupations are given in square brackets below each isosurface plot. These NOs were analyzed for their decomposition in terms of atomic orbital (AO) wt% to facilitate bonding analysis. Lower panel: NOs describe one σ and two π bondings; and Top panel: The NOs with involvement of CO π orbitals and double d-shell.

S7 Spin-orbit coupling matrix elements for (Fv)$M_2(CO)_4$ (M = Ru, Fe) for the three lowest singlet and triplet states at the key geometries

The spin-orbit coupling (SOC) was calculated for the (Fv)$M_2(CO)_4$ complexes (M = Ru, Fe) at the DFT-optimized geometries corresponding to various intermediate states. The SOC was treated perturbatively using the state-interaction (RASSI) method within the OpenMOLCAS suite[S7], using XMS-CASPT2(10e,12o) wavefunctions for the calculations, which was used to construct adiabatic potential energy curves for both complexes. The matrix elements of the complex SO Hamiltonian, denoted as $\langle \Psi_i | H_{\mathrm{SO}} | \Psi_j \rangle$, were computed across spin components (M_s) of the spin-free eigenstates (SFS) and are reported in cm^{-1}.

The magnitude of the SOC between two spin-free states, Ψ_i and Ψ_j, was evaluated using the expression: $\langle \Psi_i | H_{\mathrm{SO}} | \Psi_j \rangle = \sqrt{\sum_{M_s} \sum_{M_s'} |\langle \Psi_{i,M_s} | H_{\mathrm{SO}} | \Psi_{j,M_s'} \rangle|^2}$ where M_s and $M_s{}'$ represent the spin projections of the respective spin-free eigenstates, and the summation accounts for all spin projections, reflecting the total contribution of SOC. This methodology calculates the magnitude of the SOC between specific diabatic or adiabatic states, providing a comprehensive understanding of the role of spin-orbit interactions in excited-state dynamics and spectroscopic properties. H_{SO} matrices for Ru and Fe across different intermediates are presented in Supporting Table S15, Table S16, Table S17 Table S18, and Table S19.

Table S15: Complex Spin–orbit (SO) Hamiltonian Matrix Elements[a] Across Spin Components (M_s) of Spin-free Eigenstates (SFS)[b]: S_0, S_1, S_2, and T_1, T_2, T_3 — at the DFT-optimized S_0 Geometry of (Fv)$Ru_2(CO)_4$, Obtained from a SO-SA(5S,5T)-(XMS)-CAS(10e,12o)PT2 Calculation. Complex SO–Coupling Matrix Elements, represented as (a,b), are Expressed in cm^{-1}. The Color Shading for the Matrix Elements Indicates the Magnitude of the SO–Coupling: Light Gray: 0 cm^{-1}, and Black: >0 cm^{-1}.

SFS		S_0	S_1	S_2		T_1			T_2			T_3	
	M_s	0	0	0	−1	0	+1	−1	0	+1	−1	0	+1
S_0	0	(0,0)	(0,0)	(0,0)	(-217,0)	(0,0)	(-217,0)	(0,0)	(0,0)	(0,0)	(0,-239)	(0,0)	(0,239)
S_1	0	(0,0)	(0,0)	(0,0)	(0,0)	(0,0)	(0,0)	(-260,0)	(0,0)	(-260,0)	(0,0)	(0,-504)	(0,0)
S_2	0	(0,0)	(0,0)	(0,0)	(255,0)	(0,0)	(255,0)	(0,0)	(0,0)	(0,0)	(0,-452)	(0,0)	(0,452)
	−1	(-217,0)	(0,0)	(255,0)	(0,0)	(0,0)	(0,0)	(0,0)	(-254,0)	(0,0)	(0,-461)	(0,0)	(0,0)
T_1	0	(0,0)	(0,0)	(0,0)	(0,0)	(0,0)	(0,0)	(254,0)	(0,0)	(-254,0)	(0,0)	(0,0)	(0,0)
	+1	(-217,0)	(0,0)	(255,0)	(0,0)	(0,0)	(0,0)	(0,0)	(254,0)	(0,0)	(0,0)	(0,0)	(0,461)
	−1	(0,0)	(-260,0)	(0,0)	(0,0)	(254,0)	(0,0)	(0,0)	(0,0)	(0,0)	(0,0)	(0,422)	(0,0)
T_2	0	(0,0)	(0,0)	(0,0)	(-254,0)	(0,0)	(254,0)	(0,0)	(0,0)	(0,0)	(0,422)	(0,0)	(0,422)
	+1	(0,0)	(-260,0)	(0,0)	(0,0)	(-254,0)	(0,0)	(0,0)	(0,0)	(0,0)	(0,0)	(0,422)	(0,0)
	−1	(0,239)	(0,0)	(0,452)	(0,461)	(0,0)	(0,0)	(0,0)	(0,-422)	(0,0)	(0,0)	(0,0)	(0,0)
T_3	0	(0,0)	(504,0)	(0,0)	(0,0)	(0,0)	(0,0)	(0,-422)	(0,0)	(0,-422)	(0,0)	(0,0)	(0,0)
	+1	(0,-239)	(0,0)	(0,-452)	(0,0)	(0,0)	(0,-461)	(0,0)	(0,-422)	(0,0)	(0,0)	(0,0)	(0,0)

[a]Net SO coupling between two spin-free states (SFS), Ψ_i and Ψ_j is: $\langle\Psi_i|H_{\rm SO}|\Psi_j\rangle = \sqrt{\sum_{M_s}\sum_{M_s'}|\langle\Psi_{i,M_s}|H_{\rm SO}|\Psi_{j,M_s'}\rangle|^2}$, where M_s and M_s' are spin-projections for i-th and j-th SFS.

[b]Approximate wavefunctions of SFSs at the DFT-optimized S_0 geometry are:
S_0: $\sim|(\sigma)^{1.9}(\sigma^*)^{0.1}\rangle$
S_1: $\sim|(\pi^*_\perp)^{1.0}(\sigma^*)^{1.0}\rangle$
S_2: $\sim|(\pi^*_\parallel)^{1.0}(\sigma^*)^{1.0}\rangle$
T_1: $\sim|(\pi^*_\perp)^{1.0}(\sigma^*)^{1.0}\rangle$
T_2: $\sim|(\pi^*_\parallel)^{1.0}(\sigma^*)^{1.0}\rangle$
T_3: $\sim|(\sigma)^{1.0}(\sigma^*)^{1.0}\rangle$

Table S16: Complex Spin–orbit (SO) Hamiltonian Matrix Elements[a] Across Spin Components (M_s) of Spin-free Eigenstates (SFS)[b]: S_0, S_1, S_2, and T_1, T_2, T_3 — at the DFT-optimized *syn* T_1 Geometry of (Fv)$Ru_2(CO)_4$, Obtained from a SO-SA(5S,5T)-(XMS)-CAS(10e,12o)PT2 Calculation. Complex SO–Coupling Matrix Elements, represented as (a,b), are Expressed in cm^{-1}. The Color Shading for the Matrix Elements Indicates the Magnitude of the SO–Coupling: Light Gray: 0 cm^{-1}, Darker Gray: 0–50 cm^{-1}, and Black: >50 cm^{-1}.

SFS		S_0	S_1	S_2		T_1			T_2			T_3	
	M_s	0	0	0	−1	0	+1	−1	0	+1	−1	0	+1
S_0	0	(0,0)	(0,0)	(0,0)	(8,-37)	(0,0)	(8,37)	(0,0)	(0,-296)	(0,0)	(-210,-553)	(0,0)	(-210,553)
S_1	0	(0,0)	(0,0)	(0,0)	(0,0)	(0,264)	(0,0)	(7,-9)	(0,0)	(7,9)	(0,0)	(0,-120)	(0,0)
S_2	0	(0,0)	(0,0)	(0,0)	(128,538)	(0,0)	(128,-538)	(0,0)	(0,56)	(0,0)	(-18,14)	(0,0)	(-18,-14)
	−1	(8,37)	(0,0)	(128,-538)	(0,0)	(0,0)	(0,0)	(0,0)	(-122,513)	(0,0)	(0,-163)	(0,0)	(0,0)
T_1	0	(0,0)	(0,-264)	(0,0)	(0,0)	(0,0)	(0,0)	(122,513)	(0,0)	(-122,513)	(0,0)	(0,0)	(0,0)
	+1	(8,-37)	(0,0)	(128,538)	(0,0)	(0,0)	(0,0)	(0,0)	(122,513)	(0,0)	(0,0)	(0,0)	(0,163)
	−1	(0,0)	(7,9)	(0,0)	(0,0)	(122,-513)	(0,0)	(0,0)	(0,0)	(0,0)	(0,0)	(-57,89)	(0,0)
T_2	0	(0,296)	(0,0)	(0,-56)	(-122,-513)	(0,0)	(122,-513)	(0,0)	(0,0)	(0,0)	(57,89)	(0,0)	(57,89)
	+1	(0,0)	(7,-9)	(0,0)	(0,0)	(-122,-513)	(0,0)	(0,0)	(0,0)	(0,0)	(0,0)	(57,89)	(0,0)
	−1	(-210,553)	(0,0)	(-18,-14)	(0,163)	(0,0)	(0,0)	(0,0)	(57,-89)	(0,0)	(0,0)	(0,0)	(0,0)
T_3	0	(0,0)	(0,120)	(0,0)	(0,0)	(0,0)	(0,0)	(-57,-89)	(0,0)	(57,-89)	(0,0)	(0,0)	(0,0)
	+1	(-210,-553)	(0,0)	(-18,14)	(0,0)	(0,0)	(0,-163)	(0,0)	(-57,-89)	(0,0)	(0,0)	(0,0)	(0,0)

[a]Net SO coupling between two spin-free states (SFS), Ψ_i and Ψ_j is: $\langle \Psi_i | H_{\mathrm{SO}} | \Psi_j \rangle = \sqrt{\sum_{M_s} \sum_{M_s'} |\langle \Psi_{i,M_s} | H_{\mathrm{SO}} | \Psi_{j,M_s'} \rangle|^2}$, where M_s and M_s' are spin-projections for i-th and j-th SFS.

[b]Approximate wavefunctions of SFSs at the DFT-optimized *syn* T_1 geometry ($\phi = 40.9°$) are:
S_0: $\sim|(\sigma)^{1.4}(\sigma^*)^{0.6}\rangle$
S_1: $\sim|(\pi_{\parallel})^{1.4}(\pi_{\parallel}^*)^{1.6}(\sigma)^{1.6}(\sigma^*)^{1.4}\rangle$
S_2: $\sim|(\pi_{\perp})^{1.4}(\pi_{\perp}^*)^{1.6}(\sigma)^{1.6}(\sigma^*)^{1.4}\rangle$
T_1: $\sim|(\sigma)^{1.0}(\sigma^*)^{1.0}\rangle$
T_2: $\sim|(\pi_{\parallel})^{1.4}(\pi_{\parallel}^*)^{1.6}(\sigma)^{1.6}(\sigma^*)^{1.4}\rangle$
T_3: $\sim|(\pi_{\perp})^{1.4}(\pi_{\perp}^*)^{1.6}(\sigma)^{1.6}(\sigma^*)^{1.4}\rangle$

Table S17: Complex Spin–orbit (SO) Hamiltonian Matrix Elements[a] Across Spin Components (M_s) of Spin-free Eigenstates (SFS)[b]: S_0, S_1, S_2, and T_1, T_2, T_3 — at the DFT-optimized *syn*-to-*anti* Rotation Saddle Point T_1 Geometry of (Fv)$Ru_2(CO)_4$, Obtained from a SO-SA(5*S*,5*T*)-(XMS)-CAS(10e,12o)PT2 Calculation. Complex SO–Coupling Matrix Elements, represented as (a,b), are Expressed in cm^{-1}. The Color Shading for the Matrix Elements Indicates the Magnitude of the SO–Coupling: Light Gray: 0 cm^{-1}, Darker Gray: 0–50 cm^{-1}, and Black: >50 cm^{-1}.

SFS		S_0	S_1	S_2		T_1			T_2			T_3	
	M_s	0	0	0	−1	0	+1	−1	0	+1	−1	0	+1
S_0	0	(0,0)	(0,0)	(0,0)	(0,0)	(0,0)	(0,0)	(21,-55)	(0,567)	(21,55)	(235,-344)	(0,78)	(235,344)
S_1	0	(0,0)	(0,0)	(0,0)	(-20,56)	(0,571)	(-20,-56)	(0,-2)	(0,1)	(0,2)	(4,0)	(0,135)	(4,0)
S_2	0	(0,0)	(0,0)	(0,0)	(-223,342)	(0,-80)	(-223,-342)	(-3,-1)	(0,-126)	(-3,1)	(3,-7)	(0,-2)	(3,7)
	−1	(0,0)	(-20,-56)	(-223,-342)	(0,0)	(0,0)	(0,0)	(0,0)	(-122,513)	(0,0)	(0,-575)	(-43,-39)	(0,0)
T_1	0	(0,0)	(0,-571)	(0,80)	(0,0)	(0,0)	(0,0)	(122,513)	(0,0)	(-122,513)	(43,-39)	(0,0)	(-43,-39)
	+1	(0,0)	(-20,56)	(-223,342)	(0,0)	(0,0)	(0,0)	(0,0)	(122,513)	(0,0)	(0,0)	(43,-39)	(0,575)
	−1	(21,55)	(0,2)	(-3,1)	(0,0)	(122,-513)	(0,0)	(0,0)	(0,0)	(0,0)	(0,-2)	(-50,-71)	(0,0)
T_2	0	(0,567)	(0,-1)	(0,126)	(-122,-513)	(0,0)	(122,-513)	(0,0)	(0,0)	(0,0)	(50,-71)	(0,0)	(-50,-71)
	+1	(21,-55)	(0,-2)	(-3,1)	(0,0)	(-122,-513)	(0,0)	(0,0)	(0,0)	(0,0)	(0,0)	(50,-71)	(0,2)
	−1	(235,344)	(4,0)	(3,7)	(0,575)	(43,39)	(0,0)	(0,2)	(50,71)	(0,0)	(0,0)	(0,0)	(0,0)
T_3	0	(0,-78)	(0,-135)	(0,2)	(-43,39)	(0,0)	(43,39)	(-50,71)	(0,0)	(50,71)	(0,0)	(0,0)	(0,0)
	+1	(235,-344)	(4,0)	(3,-7)	(0,0)	(-43,39)	(0,-575)	(0,0)	(-50,71)	(0,-2)	(0,0)	(0,0)	(0,0)

[a]Net SO coupling between two spin-free states (SFS), Ψ_i and Ψ_j is: $\langle \Psi_i | H_{\mathrm{SO}} | \Psi_j \rangle = \sqrt{\sum_{M_s} \sum_{M_s'} |\langle \Psi_{i,M_s} | H_{\mathrm{SO}} | \Psi_{j,M_s'} \rangle|^2}$, where M_s and M_s' are spin-projections for i-th and j-th SFS.

[b]Approximate wavefunctions of SFSs at the DFT-optimized *syn*-to-*anti* rotation saddle point T_1 geometry ($\phi = 95.0°$) are:

S_0: $\sim|(\sigma)^{1.0}(\sigma^*)^{1.0}\rangle$
S_1: $\sim|(\pi_{\parallel})^{1.4}(\pi_{\parallel}^*)^{1.6}(\sigma)^{1.5}(\sigma^*)^{1.5}\rangle$
S_2: $\sim|(\pi_{\perp})^{1.4}(\pi_{\perp}^*)^{1.6}(\sigma)^{1.6}(\sigma^*)^{1.4}\rangle$
T_1: $\sim|(\sigma)^{1.0}(\sigma^*)^{1.0}\rangle$
T_2: $\sim|(\pi_{\parallel})^{1.4}(\pi_{\parallel}^*)^{1.6}(\sigma)^{1.5}(\sigma^*)^{1.5}\rangle$
T_3: $\sim|(\pi_{\perp})^{1.4}(\pi_{\perp}^*)^{1.6}(\sigma)^{1.6}(\sigma^*)^{1.4}\rangle$

Table S18: Complex Spin–orbit (SO) Hamiltonian Matrix Elements[a] Across Spin Components (M_s) of Spin-free Eigenstates (SFS)[b]: S_0, S_1, S_2, and T_1, T_2, T_3 — at the DFT-optimized *anti* T_1 Geometry of (Fv)$Ru_2(CO)_4$, Obtained from a SO-SA(5*S*,5*T*)-(XMS)-CAS(10e,12o)PT2 Calculation. Complex SO–Coupling Matrix Elements, represented as (*a*,*b*), are Expressed in cm^{-1}. The Color Shading for the Matrix Elements Indicates the Magnitude of the SO–Coupling: Light Gray: 0 cm^{-1}, Darker Gray: 0–50 cm^{-1}, and Black: >50 cm^{-1}.

SFS		S_0	S_1	S_2		T_1			T_2			T_3	
	M_s	0	0	0	−1	0	+1	−1	0	+1	−1	0	+1
S_0	0	(0,0)	(0,0)	(0,0)	(-1,1)	(0,0)	(-1,-1)	(0,0)	(0,-817)	(15,0)	(15,0)	(0,0)	(15,0)
S_1	0	(0,0)	(0,0)	(0,0)	(0,0)	(0,814)	(0,0)	(-1,0)	(0,0)	(0,0)	(0,0)	(0,178)	(0,0)
S_2	0	(0,0)	(0,0)	(0,0)	(-16,-3)	(0,0)	(-16,3)	(0,0)	(0,-158)	(-3,1)	(-2,1)	(0,0)	(-2,1)
	−1	(-1,1)	(0,0)	(-16,3)	(0,0)	(0,0)	(0,0)	(0,0)	(15,3)	(0,0)	(0,-834)	(0,0)	(0,0)
T_1	0	(0,0)	(0,-814)	(0,0)	(0,0)	(0,0)	(0,0)	(-15,3)	(0,0)	(15,3)	(0,0)	(0,0)	(0,0)
	+1	(-1,-1)	(0,0)	(-16,-3)	(0,0)	(0,0)	(0,0)	(0,0)	(-15,3)	(0,0)	(0,0)	(0,0)	(0,834)
	−1	(0,0)	(-1,0)	(0,0)	(0,0)	(-15,-3)	(0,0)	(0,0)	(0,0)	(0,0)	(0,0)	(-2,-1)	(0,0)
T_2	0	(0,817)	(0,0)	(0,-158)	(15,-3)	(0,0)	(-15,-3)	(0,0)	(0,0)	(0,0)	(2,-1)	(0,0)	(-2,-1)
	+1	(0,0)	(-1,0)	(0,0)	(0,0)	(15,-3)	(0,0)	(0,0)	(0,0)	(0,0)	(0,0)	(2,-1)	(0,0)
	−1	(15,0)	(0,0)	(-2,1)	(0,834)	(0,0)	(0,0)	(0,0)	(2,1)	(0,0)	(0,0)	(0,0)	(0,0)
T_3	0	(0,0)	(0,-178)	(0,0)	(0,0)	(0,0)	(0,0)	(-2,1)	(0,0)	(2,1)	(0,0)	(0,0)	(0,0)
	+1	(15,0)	(0,0)	(-2,-1)	(0,0)	(0,0)	(0,-834)	(0,0)	(-2,1)	(0,0)	(0,0)	(0,0)	(0,0)

[a]Net SO coupling between two spin-free states (SFS), Ψ_i and Ψ_j is: $\langle \Psi_i | H_{SO} | \Psi_j \rangle = \sqrt{\sum_{M_s} \sum_{M'_s} |\langle \Psi_{i,M_s} | H_{SO} | \Psi_{j,M'_s} \rangle|^2}$, where M_s and M'_s are spin-projections for *i*-th and *j*-th SFS.

[b]Approximate wavefunctions of SFSs at the DFT-optimized *anti* T_1 geometry ($\phi = 178.9°$) are:

S_0: $\sim|(\sigma)^{1.2}(\sigma^*)^{0.8}\rangle$
S_1: $\sim|(\pi_{\parallel})^{1.4}(\pi^*_{\parallel})^{1.6}(\sigma)^{1.4}(\sigma^*)^{1.6}\rangle$
S_2: $\sim|(\pi_{\perp})^{1.4}(\pi^*_{\perp})^{1.6}(\sigma)^{1.6}(\sigma^*)^{1.4}\rangle$
T_1: $\sim|(\sigma)^{1.0}(\sigma^*)^{1.0}\rangle$
T_2: $\sim|(\pi_{\parallel})^{1.4}(\pi^*_{\parallel})^{1.6}(\sigma)^{1.4}(\sigma^*)^{1.6}\rangle$
T_3: $\sim|(\pi_{\perp})^{1.4}(\pi^*_{\perp})^{1.6}(\sigma)^{1.6}(\sigma^*)^{1.4}\rangle$

Table S19: Complex Spin–orbit (SO) Hamiltonian Matrix Elements[a] Across Spin Components (M_s) of Spin-free Eigenstates (SFS)[b]: S_0, S_1, S_2, and T_1, T_2, T_3 — at the DFT-optimized S_0 Geometry of (Fv)$Fe_2(CO)_4$, Obtained from a SO-SA(5S,5T)-(XMS)-CAS(10e,12o)PT2 Calculation. Complex SO–Coupling Matrix Elements, represented as (a,b), are Expressed in cm^{-1}. The Color Shading for the Matrix Elements Indicates the Magnitude of the SO–Coupling: Light Gray: 0 cm^{-1}, and Black: >0 cm^{-1}.

SFS		S_0	S_1	S_2		T_1			T_2			T_3	
	M_s	0	0	0	−1	0	+1	−1	0	+1	−1	0	+1
S_0	0	(0,0)	(0,0)	(0,0)	(65,0)	(0,0)	(-65,0)	(0,51)	(0,0)	(0,-51)	(0,0)	(0,0)	(0,0)
S_1	0	(0,0)	(0,0)	(0,0)	(0,0)	(0,0)	(0,0)	(0,0)	(0,-367)	(0,0)	(-105,0)	(0,0)	(105,0)
S_2	0	(0,0)	(0,0)	(0,0)	(-106,0)	(0,0)	(-106,0)	(0,-191)	(0,0)	(0,-191)	(0,0)	(0,0)	(0,0)
	−1	(65,0)	(0,0)	(-106,0)	(0,0)	(0,0)	(0,0)	(0,341)	(0,0)	(0,0)	(0,0)	(102,0)	(0,0)
T_1	0	(0,0)	(0,0)	(0,0)	(0,0)	(0,0)	(0,0)	(0,0)	(0,0)	(0,0)	(-102,0)	(0,0)	(102,0)
	+1	(65,0)	(0,0)	(-106,0)	(0,0)	(0,0)	(0,0)	(0,0)	(0,0)	(0,-341)	(0,0)	(-102,0)	(0,0)
	−1	(0,-51)	(0,0)	(0,191)	(0,-341)	(0,0)	(0,0)	(0,0)	(0,0)	(0,0)	(0,0)	(0,-168)	(0,0)
T_2	0	(0,0)	(0,367)	(0,0)	(0,0)	(0,0)	(0,0)	(0,0)	(0,0)	(0,0)	(0,-168)	(0,0)	(0,-168)
	+1	(0,-51)	(0,0)	(0,-191)	(0,0)	(0,0)	(0,341)	(0,0)	(0,0)	(0,0)	(0,0)	(0,-168)	(0,0)
	−1	(0,0)	(-105,0)	(0,0)	(0,0)	(-102,0)	(0,0)	(0,0)	(0,168)	(0,0)	(0,0)	(0,0)	(0,0)
T_3	0	(0,0)	(0,0)	(0,0)	(102,0)	(0,0)	(-102,0)	(0,168)	(0,0)	(0,168)	(0,0)	(0,0)	(0,0)
	+1	(0,0)	(-105,0)	(0,0)	(0,0)	(102,0)	(0,0)	(0,0)	(0,168)	(0,0)	(0,0)	(0,0)	(0,0)

[a]Net SO coupling between two spin-free states (SFS), Ψ_i and Ψ_j is: $\langle\Psi_i|H_{SO}|\Psi_j\rangle = \sqrt{\sum_{M_s}\sum_{M_s'}|\langle\Psi_{i,M_s}|H_{SO}|\Psi_{j,M_s'}\rangle|^2}$, where M_s and M_s' are spin-projections for i-th and j-th SFS.

[b]Approximate wavefunctions of SFSs at the DFT-optimized S_0 geometry are:
S_0: $\sim|(\sigma)^{1.7}(\sigma^*)^{0.3}\rangle$
S_1: $\sim|(\pi^*_\perp)^{1.0}(\sigma^*)^{1.0}\rangle$
S_2: $\sim|(\pi^*_\parallel)^{1.0}(\sigma^*)^{1.0}\rangle$
T_1: $\sim|(\pi^*_\perp)^{1.0}(\sigma^*)^{1.0}\rangle$
T_2: $\sim|(\pi^*_\parallel)^{1.0}(\sigma^*)^{1.0}\rangle$
T_3: $\sim|(\sigma)^{1.0}(\sigma^*)^{1.0}\rangle$

S8 Diabatic potential energy surfaces near the Frank-Condon (FC) region for $(Fv)M_2(CO)_4$ (M = Ru, Fe)

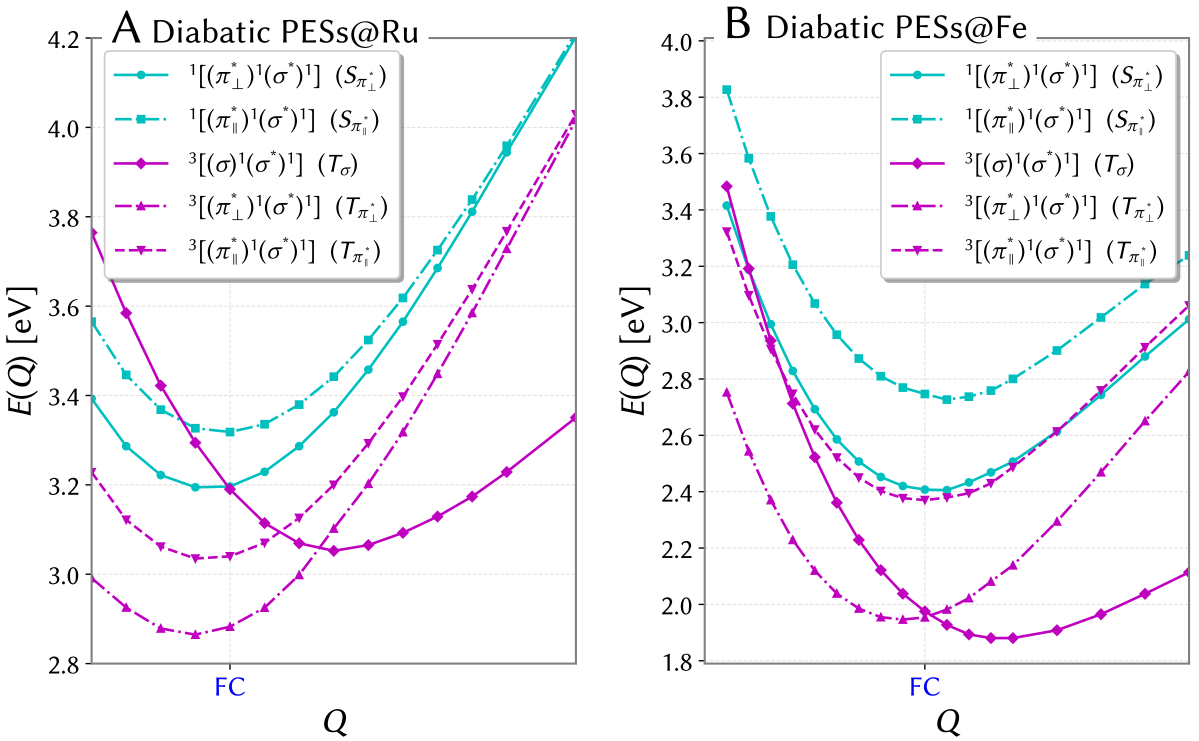

Figure S15: Diabatic PESs near the FC geometry are presented for singlet excited states ($S_{\pi_\perp^*}$, $S_{\pi_\parallel^*}$) and triplet excited states ($T_{\pi_\perp^*}$, $T_{\pi_\parallel^*}$, T_σ). These diabatic PESs are derived from the adiabatic PESs corresponding to the two lowest singlet excited states (S_1 and S_2) and triplet states (T_1, T_2, T_3) along the linearly interpolated coordinate (Q) connecting the FC geometry and the *syn* T_1 geometry. **(A)** $(Fv)Ru_2(CO)_4$, **(B)** $(Fv)Fe_2(CO)_4$

To compute the energy gap (ΔE) and reorganization energy (λ) for singlet-to-triplet transitions *via* intersystem crossing (ISC) in Marcus theory, we first generated the adiabatic potential energy surfaces (PESs) for the three lowest singlet states (S_0, S_1, and S_2) and triplet states (T_1, T_2, and T_3) along a reaction coordinate (Q) that connects the Franck-Condon (FC) geometry to the *syn*-T_1 geometry for both complexes. These PESs were computed using state-averaged extended-multistate perturbation theory (SA-XMS-CASPT2(10e,12o)). The reaction coordinate was sampled with 40 linearly interpolated points, supplemented by additional extrapolated points to capture the parabolic nature of the PESs.

Two singlet diabatic potential energy surfaces (PESs) were derived from the adiabatic excited singlet states (S_1 and S_2), while three diabatic triplet PESs were obtained from the lowest three adiabatic triplet states (T_1, T_2, and T_3). Adiabatic PESs are ordered by energy, whereas diabatic PESs are defined by the nature or character of the states involved and are constructed

by connecting states with similar electronic configurations along Q. The two resulting diabatic singlet states correspond to the configurations $^1[(\pi^*_\perp)^1(\sigma^*)^1]$ and $^1[(\pi^*_\parallel)^1(\sigma^*)^1]$, both of which are bright at the equilibrium geometry for both complexes (Figure S15, cyan). The corresponding three diabatic triplet states are $^3[(\pi^*_\perp)^1(\sigma^*)^1]$, $^3[(\pi^*_\parallel)^1(\sigma^*)^1]$, and $^3[(\sigma)^1(\sigma^*)^1]$ (Figure S15, magenta). Figure S15A (Ru) and Figure S15B (Fe) zoom into the region near the Franck-Condon (FC) geometry, highlighting the diabatic state behavior.

These diabatic surfaces were fitted using a harmonic oscillator model: $E(Q) = a(Q-b)^2+c$. SOC between the singlet and triplet diabatic states was also calculated at each interpolated and extrapolated point.

S9 Intersystem crossing mechanism of the $(Fv)Os_2(CO)_4$ complex

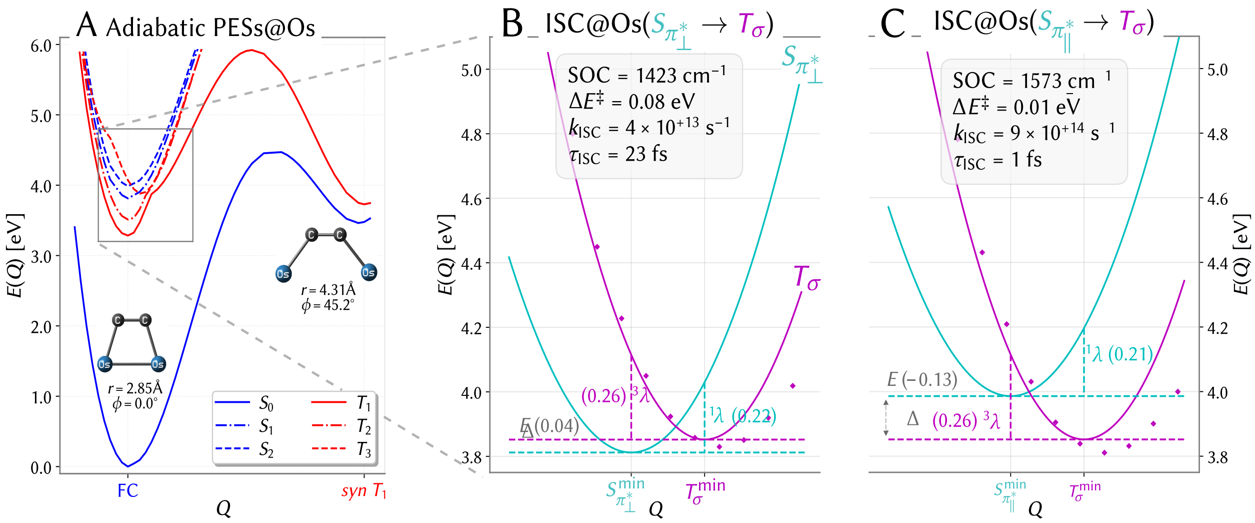

Figure S16: ISC rates in $(Fv)Os_2(CO)_4$. **A:** Adiabatic PESs along the isomerization coordinate from the Franck–Condon region to the *syn*-T_1 biradical minimum. **B,C:** Diabatic PESs for ISC from $S_{\pi^*_\perp}$ to T_σ and $S_{\pi^*_\parallel}$ to T_σ, respectively. Quadratic fits are shown in cyan and magenta; adiabatic surfaces are shown in blue (singlet) and red (triplet). Activation barriers ($\Delta E^\ddagger$) and reorganization energies (λ) are given in eV.

To complete the Group 8 series and deepen our understanding of ISC dynamics in transition metal fulvalene complexes, we studied the osmium analogue $(Fv)Os_2(CO)_4$. Despite efficient photoisomerization,[S8,S9] $(Fv)Os_2(CO)_4$ exhibits thermal irreversibility, failing to revert thermally to its parent form even at 275°C,[S9] which limits its potential for MOST applications that require both fast photoconversion and facile thermal back-reaction.

Our high-level XMS-CASPT2 calculations along the isomerization pathway—from the FC region to the *syn*-T_1 biradical intermediate— show that $(Fv)Os_2(CO)_4$ undergoes ultrafast ISC due to the osmium's strong SOC, with matrix elements reaching up to ∼1600 cm^{-1}. Applying Marcus theory to the diabatic potential energy surfaces yields near barrierless ISC transitions in the Marcus boundary regime, with rates of 4×10^{13} s^{-1} ($\tau = 23$ fs) for the $S_{\pi^*_\perp} \rightarrow T_\sigma$ channel and an even faster 9×10^{14} s^{-1} ($\tau = 1$ fs) for the $S_{\pi^*_\parallel} \rightarrow T_\sigma$ channel. These kinetics are similar to those computed for the ruthenium analogue, confirming that ISC in these systems is dominated by strong SOC and favorable energetic alignment near excited singlet minima.

However, and unlike $(Fv)Ru_2(CO)_4$, the thermal back-reaction barrier in the Os compound is significantly higher (1.76 eV vs. 1.01 eV in Ru), due to the concerted cleavage of Ru–C bonds and C–C bond formation. This large barrier precludes reversible photoisomerization. Thus, while $(Fv)Os_2(CO)_4$ confirms the trend of ultrafast ISC kinetics across Group 8 fulvalene complexes, its high thermal barrier limits practical utility in reversible solar thermal storage.

S10 Intersystem crossing mechanism of the $(Fv)FeRu(CO)_4$ complex

Heterobimetallic fulvalene complexes, such as $(Fv)RuFe(CO)_4$, have been proposed as cost-effective and environmentally benign alternatives to the well-studied $(Fv)Ru_2(CO)_4$ system, which could offer comparable energy storage capacities.[S9,S10] The incorporation of Ru was expected to enhance SOC, which is a key factor in enabling efficient ISC in these systems. Indeed, computed SOC matrix elements for $(Fv)RuFe(CO)_4CO_4$, with $\langle S_{\pi^*_\perp}|H_{SO}|T_\sigma\rangle = 446$ cm^{-1} and $\langle S_{\pi^*_\parallel}|H_{SO}|T_\sigma\rangle = 428$ cm^{-1}, are comparable to those in $(Fv)Ru_2(CO)_4$.

Still, $(Fv)RuFe(CO)_4CO_4$ is photoinert, with a photochemical behavior similar to the Fe analogue.[S4,S9] This observation indicates that SOC magnitude alone does not dictate ISC efficiency. To rationalize this discrepancy, we analyzed the singlet–triplet energy ($\Delta E = E_{S_1} - E_{T_1}$) at the FC point , which critically influences ISC rates within Marcus theory. The calculated ΔE= 0.31 eV at the FC geometry is substantially higher than the near-degenerate gaps in $(Fv)Ru_2(CO)_4$ (0.01 eV) and $(Fv)Os_2(CO)_4$ (–0.09 eV), but smaller than in $(Fv)Fe_2(CO)_4$ (0.45 eV). This places $(Fv)RuFe(CO)_4$ near the Marcus inverted regime, where ISC is kinetically disfavored despite appreciable SOC. By contrast, the minimal singlet–triplet gaps in $(Fv)Ru_2(CO)_4$ and $(Fv)Os_2(CO)_4$ allow rapid ISC *via* near barrierless transitions in the Marcus boundary regime.

These results highlight that the excited-state energy topology—particularly the FC singlet–triplet gap—is a more reliable predictor of ISC efficiency than SOC magnitude. The contrasting behaviors of $(Fv)RuFe(CO)_4$ and $(Fv)Ru_2(CO)_4$ demonstrate that a favorable energetic alignment is essential to enable ultrafast ISC and effective photoconversion, which are critical for MOST applications.

In summary, the pronounced mismatch between SOC strength ($(Fv)RuFe(CO)_4 \approx (Fv)Ru_2(CO)_4 \gg (Fv)Fe_2(CO)_4$) and ISC efficiency ($(Fv)Ru_2(CO)_4 \gg (Fv)RuFe(CO)_4 \approx (Fv)Fe_2(CO)_4$) confirms that Marcus energetics—not SOC magnitude—serve as the key determinant of photoreactivity in this family of transition metal fulvalene complexes.

S11 Assessment of semiclassical Marcus vs. Marcus–Levich–Jortner rates and vibronic-coupling in $(Fv)M_2(CO)_4$ complexes (M = Fe, Ru, Os)

ISC between singlet and triplet states in bimetallic complexes such as $(Fv)M_2(CO)_4$ (M=Fe,Ru,Os) is fundamentally governed by two primary factors: SOC, which facilitates electronic spin-flip transitions, and nuclear reorganization, which modulates transition rates through vibrational dynamics. Given that ISC is closely associated with metal–metal bond dissociation in the triplet state, low-frequency metal–metal (M–M) stretching modes—typically below 200 cm^{-1}—are expected to play a crucial role in mediating ISC. This section evaluates whether the semi-classical Marcus theory[S11,S12] captures ISC dynamics sufficiently well or whether quantum vibronic corrections, here provided by the Marcus-Levich-Jortner (MLJ) theory[S13–S15] are necessary. A detailed comparison of ISC rates from both models is presented to elucidate the mechanistic role of low-frequency vibrational modes.

Theoretical framework

In the semiclassical Marcus theory, ISC is modeled analogously to nonradiative electron transfer, governed by thermally activated nuclear reorganization. The rate constant is given by:

$$k_{\text{Marcus}} = \frac{2\pi}{\hbar}|H_{\text{SO}}|^2 \frac{1}{\sqrt{4\pi\lambda k_B T}} \exp\left(-\frac{(\Delta E + \lambda)^2}{4\lambda k_B T}\right), \tag{1}$$

where H_{SO} denotes the SOC matrix element at the crossing geometry, ΔE is the energy difference between the singlet and triplet diabatic states, and λ is the total reorganization energy. This model is generally valid in the *normal* ($|\Delta E| < \lambda$) and *boundary* ($|\Delta E| \approx \lambda$) regimes, but it tends to underestimate rates in the *inverted* regime ($|\Delta E| > \lambda$).

The MLJ theory extends the Marcus equation by incorporating quantized vibrational modes that influence ISC rates through vibronic coupling,

$$k_{\text{MLJ}} = \frac{2\pi}{\hbar}|H_{\text{SO}}|^2 \sum_{v_i}\left[\prod_i \left(\frac{S_i^{v_i}}{v_i!}e^{-S_i}\right)\right] \exp\left(-\frac{(\Delta E + \sum_i v_i \hbar\omega_i)^2}{4\sigma^2}\right), \tag{2}$$

where S_i are the Huang–Rhys (HR) factors, ω_i are the vibrational frequencies, and σ^2 is the thermal broadening factor:

$$\sigma^2 = \sum_i S_i(\hbar\omega_i)^2 \coth\left(\frac{\hbar\omega_i}{2k_B T}\right). \tag{3}$$

MLJ theory is important when high-frequency modes strongly couple to electronic states, as it captures essential quantum vibrational effects that are neglected in semiclassical models. In this work, the reorganization energy considered corresponds exclusively to the inner-sphere contribution (gas-phase potential energy surface calculations).

Vibrational analyses were carried out at the ground-state (S_0) optimized FC geometry because the structural differences between S_0 and the excited-state minimum ($S_{\pi_\perp^*}^{\min}$) are negligible (RMSD < 0.1 Å). A common set of normal modes and frequencies from the S_0 geometry was mapped onto the $S_{\pi_\perp^*}^{\min}$ geometry and used for all ISC rate calculations of the $S_{\pi_\perp^*} \rightarrow T_\sigma$ transition for FvM_2 (M = Fe, Ru, Os).

The vibrational modes were obtained by diagonalizing the mass-weighted Hessian at the FC geometry. Geometric displacements between singlet excited-state ($S_{\pi_\perp^*}^{\min}$) and triplet-state ($T_\sigma^{\min}$) minima were aligned *via* the Kabsch algorithm and projected onto S_0 normal modes to compute mode-specific displacements ΔQ_i. Mode-specific HR factors and reorganization energies were calculated as:

$$S_i = \frac{\omega_i}{2\hbar}(\Delta Q_i)^2, \tag{4}$$

$$\lambda_i = S_i \hbar \omega_i, \tag{5}$$

with the total inner-sphere reorganization energy given by $\lambda = \sum_i \lambda_i$.

For systems dominated by a single strongly coupled mode ($S_i > 1.0$), the following single-mode MLJ expression can be used:

$$k_{\mathrm{MLJ}}^{\mathrm{single}} = \frac{2\pi}{\hbar}|H_{\mathrm{SO}}|^2 e^{-S_{\max}} \sum_{v=0}^{v_{\max}} \frac{S_{\max}^v}{v!} \exp\left(-\frac{(\Delta E + v\hbar\omega_{\max})^2}{4\sigma_{\max}^2}\right). \tag{6}$$

ISC rates were computed using both single- and multimode MLJ expressions, as appropriate. The vibrational summation was truncated at $v_i = 12$ to ensure convergence; terms contributing less than 10^{-10} were neglected. FC factors were evaluated using the displaced harmonic oscillator approximation.

All the calculations employed data calculated at the XMS-CASPT2 level to ensure accurate treatment of electronic correlations. SOC matrix elements were computed at minimum-energy crossing points. All rate expressions were implemented in `Python`, using `Numba`-accelerated routines and parallelized over vibrational configurations. Modes with strong vibronic coupling ($S_i > 1.0$), as well as additional modes with $S_i > 0.1$ and frequencies between 20–600 cm^{-1}, were included. This window encompasses key low-frequency modes such as metal–metal stretches and reorganizational vibrations that facilitate ISC.

Numerical Results: HR factors, normal modes, and ISC rates

Huang–Rhys factors and normal modes: The vibronic analysis of the (Fv)$M_2(CO)_4$ series (M = Ru, Os, Fe) reveals distinct ISC regimes governed by metal–metal bond dynamics. For (Fv)$Ru_2(CO)_4$ and (Fv)$Os_2(CO)_4$, low-frequency metal–metal stretching modes (below approximately 600 cm^{-1}) dominate the reorganization energies (Table S20 and Table S22). Notably, the 92 cm^{-1} Ru–Ru mode ($S = 5.1$, $\lambda = 0.058$ eV) and the 92 cm^{-1} Os–Os mode ($S = 3.5$, $\lambda = 0.040$ eV) exemplify strong Marcus-type vibronic coupling. These soft modes account for over 90% of the total reorganization energy (λ), while high-frequency modes (above 600 cm^{-1}) exhibit very weak vibronic coupling ($S < 0.1$) and contribute negligibly to the nuclear reorganization, justifying their exclusion from the vibronic analysis. The less than 10% deviation between the harmonic sum and Marcus-fitted λ values indicates mild anharmonicity but supports the validity of a semiclassical Marcus description.

In contrast, (Fv)$Fe_2(CO)_4$ lies deep within the inverted Marcus regime, characterized by very weak vibronic coupling without any dominant vibrational mode (all $S < 0.5$). As a result, the total classical Marcus reorganization energy cannot be recovered by summing contributions from individual modes, since these are small and diffusely distributed (Table S21). Here, ISC proceeds predominantly *via* electronically dominated, vibration-suppressed mechanisms, rendering semiclassical Marcus theory sufficient without requiring explicit vibronic corrections.

Because the ISC-active modes in (Fv)$Ru_2(CO)_4$ and (Fv)$Os_2(CO)_4$ occur primarily at frequencies below 600 cm^{-1} and satisfy the semiclassical condition ($\hbar\omega \ll k_B T$) at room to slightly elevated temperatures, quantum vibrational effects captured by MLJ theory do not significantly affect ISC rates. For (Fv)$Fe_2(CO)_4$, the generally weak and diffuse vibronic coupling further reduces the impact of these quantum corrections. Consequently, semiclassical Marcus theory adequately captures the ISC mechanisms across the entire (Fv)$M_2(CO)_4$ (M = Ru, Fe, Os) series, with MLJ calculations included for completeness to confirm the minimal role of quantum vibrational effects.

Table S20: Selected low-frequency vibrational modes ($20 < \omega < 600$ cm^{-1}) with significant vibronic coupling ($S_i > 0.05$) for (Fv)$Ru_2(CO)_4$. Listed are the mode indices, vibrational frequencies (ω_i), Huang–Rhys factors (S_i), Franck–Condon (FC) factors for $v = 0$, and mode-specific reorganization energies (λ_i).

Mode (i)	ω_i (cm^{-1})	S_i	FC($v = 0$)	λ_i (eV)
1	24.7	0.694	0.500	0.002
2	52.8	0.071	0.931	0.001
**6**	**92.0**	**5.115**	**0.006**	**0.058**
8	95.3	0.114	0.892	0.001
9	109.3	0.089	0.915	0.001
**12**	**172.3**	**2.088**	**0.124**	**0.045**
13	272.8	0.408	0.665	0.013
15	296.4	0.066	0.936	0.002
18	417.3	0.332	0.717	0.017
**24**	**496.1**	**0.771**	**0.463**	**0.047**
25	504.5	0.232	0.793	0.012
			Total λ (selected modes)	0.196

Note. The λ obtained from mode summation (0.196 eV) closely reproduces the Marcus-fit value for the $S_{\pi^*_\perp}$ surface (0.185 eV) (< 5.0% deviation), indicating very good agreement.

Table S21: Selected low-frequency vibrational modes ($20 < \omega < 600\,\text{cm}^{-1}$) with significant vibronic coupling ($S_i > 0.01$) for (Fv)$Fe_2(CO)_4$. Listed are the mode indices, vibrational frequencies (ω_i), Huang–Rhys factors (S_i), Franck–Condon (FC) factors for $\upsilon = 0$, and mode-specific reorganization energies (λ_i).

Mode (i)	ω_i (cm^{-1})	S_i	FC($\upsilon = 0$)	λ_i (eV)
2	50.1	0.119	0.888	0.001
4	97.1	0.045	0.956	0.001
7	106.2	0.463	0.630	0.006
11	179.4	0.166	0.847	0.004
14	315.8	0.049	0.952	0.002
18	431.7	0.072	0.930	0.004
26	516.8	0.113	0.893	0.007
		Total λ (selected modes)		0.026

Note. Good consistency between calculated (0.026 eV) and fitted (0.036 eV) λ values for the $S_{\pi^*_\perp}$ surface confirms the physical relevance of selected modes.

Table S22: Selected low-frequency vibrational modes ($20 < \omega < 600$ cm^{-1}) with significant vibronic coupling ($S_i > 0.05$) for (Fv)$Os_2(CO)_4$. Listed are the mode indices, vibrational frequencies (ω_i), Huang–Rhys factors (S_i), Franck–Condon (FC) factors for $\upsilon = 0$, and mode-specific reorganization energies (λ_i).

Mode (i)	ω_i (cm^{-1})	S_i	FC($\upsilon = 0$)	λ_i (eV)
1	21.3	0.366	0.694	0.001
2	53.2	0.510	0.601	0.003
**5**	**92.1**	**3.475**	**0.031**	**0.040**
8	99.6	0.296	0.744	0.004
**10**	**148.7**	**2.586**	**0.075**	**0.048**
13	268.1	0.209	0.831	0.007
16	371.1	0.067	0.937	0.002
18	429.3	0.077	0.925	0.003
**22**	**560.6**	**0.094**	**0.912**	**0.007**
		Total λ (selected modes)		0.069

Note. Excellent agreement between the sum of mode-specific λ (0.069 eV) and Marcus-fitted λ (0.070 eV) values for the $S_{\pi^*_\perp}$ surface (< 2% deviation) confirms the dominant role of low-frequency Os–Os stretching modes.

ISC rates and lifetimes in (Fv)Ru$_2$(CO)$_4$, (Fv)Fe$_2$(CO)$_4$, and (Fv)Os$_2$(CO)$_4$: The vibrational modes that most strongly facilitate ISC lie predominantly below approximately 600 cm^{-1}, corresponding to energies ($\hbar\omega$) that are much smaller than the thermal energy scale ($k_B T$) at room temperature and moderately elevated temperatures. Because these low-frequency modes are thermally populated, they behave effectively as a classical vibrational bath. Conversely, high-frequency modes above 600 cm^{-1} exhibit negligible vibronic coupling and contribute minimally to nuclear reorganization, further supporting the classical approximation.

To rigorously test the influence of quantum vibrational effects, ISC rates were also calculated using the MLJ formalism, which explicitly incorporates quantized vibrational modes and is particularly important for high-frequency vibrations. However, MLJ corrections led to only minor changes in ISC rate constants throughout the (Fv)M$_2$(CO)$_4$ series, demonstrating that quantum vibrational contributions are negligible for these systems. The ISC kinetics of (Fv)Ru$_2$(CO)$_4$ and (Fv)Os$_2$(CO)$_4$ differ markedly from (Fv)Fe$_2$(CO)$_4$, and given in Table S23.

Table S23: Computed intersystem crossing (ISC) rate constants, k_{ISC} (in s^{-1}), and corresponding lifetimes, $\tau = 1/k$, shown in parentheses with appropriate time units, for the $S_{\pi^*_\perp} \rightarrow T_\sigma$ transition in (Fv)Ru$_2$(CO)$_4$, (Fv)Fe$_2$(CO)$_4$, and (Fv)Os$_2$(CO)$_4$. Rates are calculated using semiclassical Marcus theory as well as single- and multimode Marcus–Jortner–Levich (MLJ) approaches.

**Method**	**(Fv)Ru$_2$(CO)$_4$**	**(Fv)Fe$_2$(CO)$_4$**	**(Fv)Os$_2$(CO)$_4$**
Marcus (semiclassical)	1.4×10^{14} (7 fs)	1.4×10^{-11} (2460 yr)	4.4×10^{13} (23 fs)
MLJ (Single-mode)	2.0×10^{13} (49 fs)	5.2×10^{-48} (6×10^{39} yr)	1.4×10^{14} (7 fs)
MLJ (Multimode)	3.2×10^{13} (32 fs)	9.4×10^{4} (11 μs)	1.0×10^{14} (10 fs)

In (Fv)Ru$_2$(CO)$_4$ and (Fv)Os$_2$(CO)$_4$, ISC occurs on a fs time scale, driven primarily by strong coupling to thermally populated metal–metal stretching modes below 200 cm^{-1}. Given that these low-frequency modes dominate reorganization and behave classically at room temperature, semiclassical Marcus theory accurately reproduces ISC rates with only minor refinements from quantum corrections.

In contrast, (Fv)Fe$_2$(CO)$_4$ resides deep within the Marcus inverted regime, characterized by weak vibronic coupling and a small total reorganization energy. This leads to severely suppressed ISC rates, with semiclassical Marcus theory predicting lifetimes on the order of thou-

sands of years. Single-mode MLJ results yield unphysically slow rates, while multimode MLJ increases rates to microsecond lifetimes—still orders of magnitude slower than the Ru and Os analogues.

The large discrepancy between multimode MLJ and the semiclassical prediction for (Fv)$Fe_2(CO)_4$ can be attributed to the MLJ assumptions in systems with weakly coupled, anharmonic modes that lack well-defined vibrational progressions, as is the case here. This is further complicated by the numerical truncation of extremely weakly coupled modes, which undermine the accuracy of ISC rate predictions in this deeply inverted regime. We thus conclude that the the MLJ theory is not effective for this complex.

In conclusion, we claim that Marcus theory is able to capture ISC mediated by low-frequency, thermally populated M–M modes in (Fv)$Ru_2(CO)_4$ and (Fv)$Os_2(CO)_4$, where the vibronic coupling is strong and vibrational modes behave classically. Conversely, quantum vibrational corrections are negligible in systems like (Fv)$Fe_2(CO)_4$, where high-frequency promoting modes are absent or only weakly coupled.

S12 Evolution of spin-orbit coupling between T_1 and S_0 states from Franck-Condon (FC) to *syn*-T_1 geometry in (Fv)$Ru_2(CO)_4$

SOC between T_1 and S_0 states along the interpolated reaction coordinate Q was calculated for the (Fv)$Ru_2(CO)_4$. The SOC was treated perturbatively using the state-interaction (RASSI) method within the OpenMOLCAS[S7], with XMS-CASPT210e,12o) wavefunctions. The SOCs were computed across spin components (M_s) of the spin-free eigenstates (SFS) and are reported in cm^{-1} in Figure S17. The magnitude of the SOC between two spin-free states, Ψ_i and Ψ_j, was evaluated using the expression: $\langle\Psi_i|H_{SO}|\Psi_j\rangle = \sqrt{\sum_{M_s}\sum_{M_s'}|\langle\Psi_{i,M_s}|H_{SO}|\Psi_{j,M_s'}\rangle|^2}$ where M_s and $M_s{}'$ represent the spin projections of the respective spin-free eigenstates, and the summation accounts for all spin projections, reflecting the total contribution of SOC.

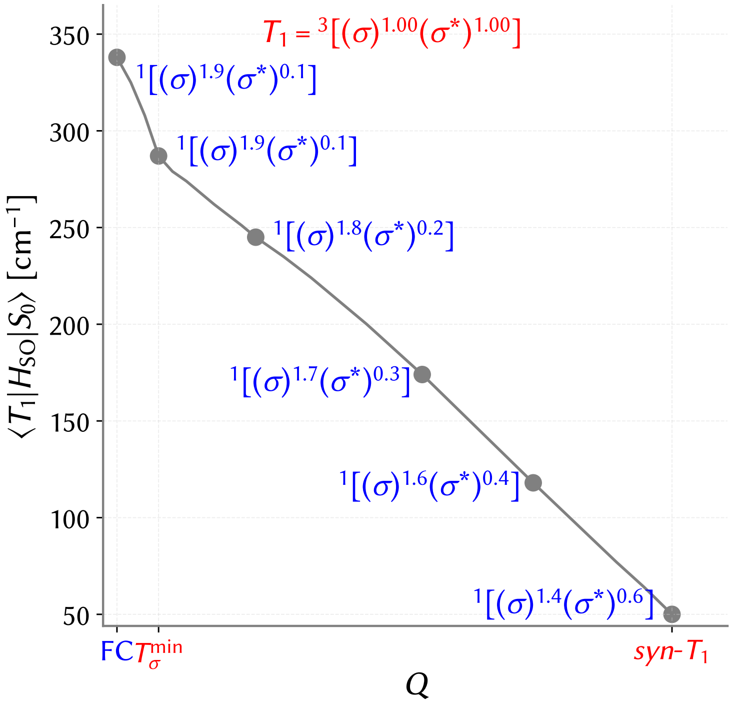

Figure S17: The gradual decrease in the spin-orbit coupling (SOC) matrix element between T_1 and S_0 along the interpolated reaction coordinate Q, from the Franck-Condon (FC) to the syn-T_1 geometries, is shown. The SOC decreases from approximately 350 cm^{-1} near the FC geometry to 50 cm^{-1} near the syn-T_1 geometry. This decline occurs as the S_0 configuration transitions from a closed-shell to a more open-shell character, driven by bond breaking, and increasingly resembles the triplet state, by El-Sayed's rule[S16]. The singlet configuration at selected points along Q is shown in blue, with σ and σ^* orbital occupations indicated. The triplet configuration, remaining constant throughout, is denoted in red at the top of the plot as $^3[(\sigma)^1(\sigma^*)^1]$.

S13 Final rearrangement in the Ru complex: High spin-orbit coupling drives triplet-to-singlet reverse intersystem crossing (rISC) and singlet photoproduct formation

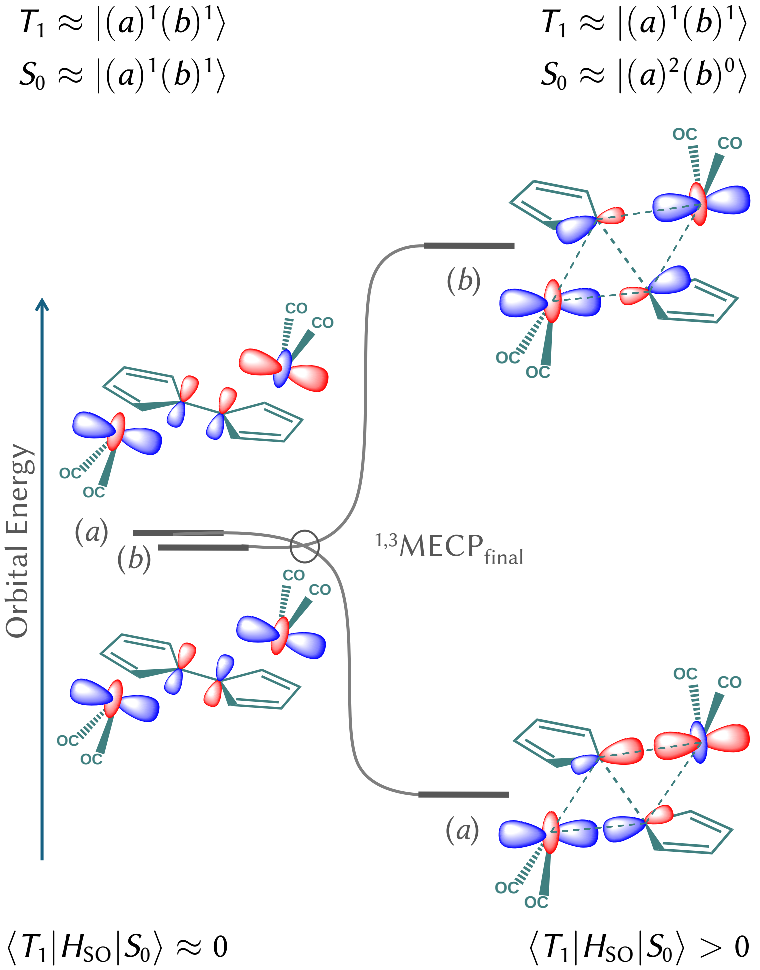

Figure S18: Schematic Orbital Correlation Diagram for the Final Rearrangement in (Fv)$Ru_2(CO)_4$ *via* ^{1,3}MECP$_{final}$. Before C−C bond cleavage and Ru−C bond formation, the singlet (S_0) and triplet (T_1) states are quasi-degenerate, both described by similar orbital configurations, $^1[(a)^1(b)^1]$ and $^3[(a)^1(b)^1]$, where a and b denote two likely molecular orbitals involving the C−C bond and Ru centers. Due to this similarity, the SOC between these states is negligible, as expected from El-Sayed's rule[S16]. After C−C bond breaking and Ru−C bond formation, the singlet state stabilizes into a closed-shell configuration, $^1[(a)^2(b)^0]$, while the triplet remains open-shell, $^3[(a)^1(b)^1]$. At the ^{1,3}MECP$_{final}$ geometry, this stabilization leads to strong SOC between S_0 and T_1, thereby promoting efficient reverse intersystem crossing (rISC) and facilitating singlet photoproduct formation.

S14 Triplet-state relaxation in $(Fv)Fe_2(CO)_4$: *syn*-to-*anti* isomerization

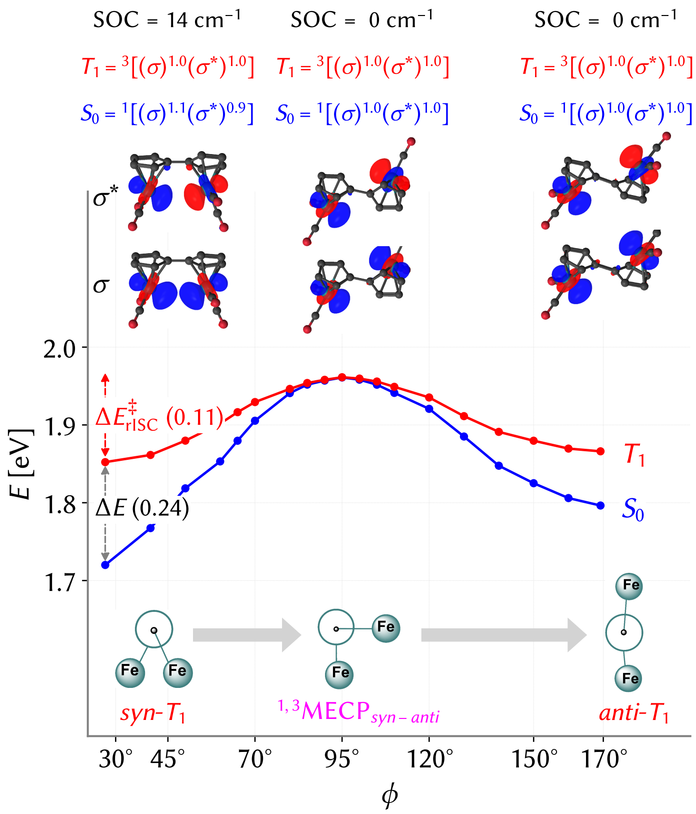

Figure S19: Stability of the triplet biradical intermediate in $(Fv)Fe_2(CO)_4$ along the *syn*-to-*anti* dihedral (ϕ) coordinate. Adiabatic PESs for the ground-state singlet (S_0, blue) and lowest triplet state (T_1, red) connect *syn*-T_1 and *anti*-T_1geometries *via* the minimum energy crossing point. Molecular orbitals (σ and σ^*) and their occupations are shown for these conformations, highlighting electronic rearrangements along the ϕ coordinate. The figure also shows the values of SOC matrix elements at critical points.

To evaluate the feasibility of photoisomerization in $(Fv)Fe_2(CO)_4$, we mapped the *syn*-to-*anti* torsional rotation pathway on the triplet surface, starting from the *syn*-T_1 biradical geometry. Despite the low barrier for reverse intersystem crossing (rISC, 0.11 eV) back to the S_0 state, the SOC decreases sharply from 230 cm^{-1} at the FC point to 14 cm^{-1} at the *syn*-T_1 minimum, and ultimately vanishes near the $^{1,3}MECP_{syn–anti}$ (SOC $= 0.00$ cm^{-1}, $\Delta E = 0.00$ eV; see Figure S19). This significant reduction in SOC suppresses deactivation via El-Sayed-forbidden, transitions[S16] owing to the similar character of the singlet and triplet states, thereby enabling unhindered *syn*-to-*anti* torsional motion on the triplet potential energy surface (Figure S19).

At the *anti*-T_1 geometry, SOC remains negligible (0.00 cm^{-1}), allowing the system to efficiently complete the torsional rotation (barrier: 0.11 eV, XMS-CASPT2), followed by a con-

certed rearrangement involving C–C bond cleavage and Fe–C bond formation (barrier: 0.34 eV, DFT). This leads to a closed-shell product where SOC is restored, thereby facilitating intersystem crossing at a second MECP. Notably, these energetic features closely resemble those observed in the Ru analogue, where the corresponding torsional and rearrangement barriers are 0.06 and 0.19 eV, respectively[S17].

These findings indicate that once the triplet biradical is accessed, both Fe and Ru systems can proceed through energetically feasible downstream photoisomerization pathways under ambient conditions. The key mechanistic distinction lies in the efficiency of the initial intersystem crossing: while (Fv)$Ru_2(CO)_4$ (and (Fv)$Os_2(CO)_4$) benefit from ISC in the Marcus boundary regime, (Fv)$Fe_2(CO)_4$ undergoes ISC in the Marcus inverted regime, resulting in a high kinetic barrier that constitutes the sole bottleneck in the photochemical cycle. Overall, the potential energy and SOC profiles (Figure S19) emphasize the critical role of spin–orbit interactions in governing the photoreactivity of diiron complexes and underscore the importance of ISC facilitation for enabling efficient light-induced rearrangements.

References

(S1) Mai, S.; Plasser, F.; Dorn, J.; Fumanal, M.; Daniel, C.; González, L. Quantitative Wave Function Analysis for Excited States of Transition Metal Complexes. *Coord. Chem. Rev.* **2018**, *361*, 74–97.

(S2) Plasser, F. TheoDORE: A Toolbox for a Detailed and Automated Analysis of Electronic Excited State Computations. *J. Chem. Phys.* **2020**, *152*, 84108.

(S3) Harpham, M. R.; Nguyen, S. C.; Hou, Z.; Grossman, J. C.; Harris, C. B.; Mara, M. W.; Stickrath, A. B.; Kanai, Y.; Kolpak, A. M.; Lee, D.; Liu, D.-J.; Lomont, J. P.; Moth-Poulsen, K.; Vinokurov, N.; Chen, L. X.; Vollhardt, K. P. C. X-ray Transient Absorption and Picosecond IR Spectroscopy of Fulvalene(tetracarbonyl)diruthenium on Photoexcitation. *Angew. Chem. Int. Ed.* **2012**, *51*, 7692–7696.

(S4) Hou, Z.; Nguyen, S. C.; Lomont, J. P.; Harris, C. B.; Vinokurov, N.; Vollhardt, K. P. C. Switching from Ru to Fe: Picosecond IR Spectroscopic Investigation of the Potential of the (Fulvalene) Tetracarbonyldiiron Frame for Molecular Solar-Thermal Storage. *Phys. Chem. Chem. Phys.* **2013**, *15*, 7466–7469.

(S5) Lennartson, A.; Lundin, A.; Börjesson, K.; Gray, V.; Moth-Poulsen, K. Tuning the Photochemical Properties of the Fulvalene-tetracarbonyl-diruthenium System. *Dalton Trans.* **2016**, *45*, 8740–8744.

(S6) Zobel, J. P.; Nogueira, J. J.; González, L. The IPEA Dilemma in CASPT2. *Chem. Sci.* **2017**, *8*, 1482–1499.

(S7) Li Manni, G.; Fdez. Galván, I.; Alavi, A.; Aleotti, F.; Aquilante, F.; Autschbach, J.; Avagliano, D.; Baiardi, A.; Bao, J. J.; Battaglia, S.; others The OpenMolcas Web: A Community-Driven Approach to Advancing Computational Chemistry. *J. Chem. Theory Comput.* **2023**, *19*, 6933–6991.

(S8) Zhu, B.; Miljanić, O. Š.; Vollhardt, K. P. C.; West, M. J. Synthesis of 2,2',3,3'-tetramethyl- and 2,2',3,3'-tetra-tert-butylfulvalene: Attractive platforms for dinuclear transition metal fragments, as exemplified by (η^5: η^5-2,2',3,3'-t-$Bu_4C_{10}H_4$)$M_2(CO)_n$ (M = Fe, Ru, Os, Mo) and first X-ray crystal structures of fulvalene diiron and diosmium complexes. *Synthesis* **2005**, *2005*, 3373–3379.

(S9) Börjesson, K.; Ćoso, D.; Gray, V.; Grossman, J. C.; Guan, J.; Harris, C. B.; Hertkorn, N.; Hou, Z.; Kanai, Y.; Lee, D.; others Exploring the Potential of Fulvalene Dimetals as Platforms for Molecular Solar Thermal Energy Storage: Computations, Syntheses, Structures, Kinetics, and Catalysis. *Chem. Eur. J.* **2014**, *20*, 15587–15604.

(S10) Kahn, A. P.; Boese, R.; Blümel, J.; Vollhardt, K. P. C. Synthesis and chemistry of heterobimetallic fulvalene complexes containing W, MO, and Rh. *J. Organomet. Chem.* **1994**,

472, 149–162.

(S11) Marcus, R. A. On the Theory of Oxidation-Reduction Reactions Involving Electron Transfer. I. *J. Chem. Phys.* **1956**, *24*, 966–978.

(S12) Marcus, R. A. Chemical and Electrochemical Electron-Transfer Theory. *Annu. Rev. Phys. Chem.* **1964**, *15*, 155–196.

(S13) Ulstrup, J.; Jortner, J. The effect of intramolecular quantum modes on free energy relationships for electron transfer reactions. *J. Chem. Phys.* **1975**, *63*, 4358–4368.

(S14) Yang, X.; Keane, T.; Delor, M.; Meijer, A. J.; Weinstein, J.; Bittner, E. R. Identifying electron transfer coordinates in donor-bridge-acceptor systems using mode projection analysis. *Nat. Commun.* **2017**, *8*, 14554.

(S15) Chaudhuri, S.; Hedström, S.; Méndez-Hernández, D. D.; Hendrickson, H. P.; Jung, K. A.; Ho, J.; Batista, V. S. Electron Transfer Assisted by Vibronic Coupling from Multiple Modes. *J. Chem. Theory Comput.* **2017**, *13*, 6000–6009.

(S16) El-Sayed, M. A. Triplet State. Its Radiative and Nonradiative Properties. *Acc. Chem. Res.* **1968**, *1*, 8–16.

(S17) Kanai, Y.; Srinivasan, V.; Meier, S. K.; Vollhardt, K. P. C.; Grossman, J. C. Mechanism of Thermal Reversal of the (Fulvalene)tetracarbonyldiruthenium Photoisomerization: Toward Molecular Solar–Thermal Energy Storage. *Angew. Chem. Int. Ed.* **2010**, *49*, 8926–8929.
